# Synthesis of the Soft Lewis Superacid Tris(4‐bromo‐2,3,5,6‐tetrafluorophenyl)borane B(C_6_F_4_Br)_3_ via C‒Ag to C‒B Transmetalation

**DOI:** 10.1002/chem.202502240

**Published:** 2025-09-21

**Authors:** Amina L. Moshtaha, Erika M. Peter, Robin Sievers, Tim‐Niclas Streit, Moritz Malischewski

**Affiliations:** ^1^ Institute of Chemistry and Biochemistry – Inorganic Chemistry Freie Universität Berlin Fabeckstr. 34/36 14195 Berlin Germany

**Keywords:** boranes, bromine, density functional calculations, fluorine, Lewis acids

## Abstract

The Lewis acid tris(4‐bromo‐2,3,5,6‐tetrafluorophenyl)borane B(C_6_F_4_Br)_3_ was synthesized via transmetalation from BBr_3_ and AgC_6_F_4_Br. The latter was prepared from 1,4‐C_6_F_4_BrSiMe_3_ with AgF and crystallized as a toluene solvate [2(AgC_6_F_4_Br)_4_•7.46(toluene)]. The solid‐state structure of free B(C_6_F_4_Br)_3_ and its Lewis base adducts with EtCN, H_2_O, and POEt_3_ was determined by single‐crystal X‐ray diffraction. DFT calculations show that B(C_6_F_4_Br)_3_ has approximately 1% higher fluoride and hydride ion affinities (FIA and HIA) than tris(pentafluorophenyl)borane B(C_6_F_5_)_3_. Similarly, the Gutmann‐Beckett method indicates an increase in Lewis acidity of 2%, whereas IR spectroscopy of the CD_3_CN adducts reveals a slightly lower ṽ(CN) vibration for B(C_6_F_4_Br)_3_. The presence of bromine atoms is expected to facilitate structural analysis of Lewis acid‐base adducts of B(C_6_F_4_Br)_3_ by single‐crystal X‐ray diffraction in the future.

## Graphical abstract

The soft Lewis acid B(C_6_F_4_Br)_3_ was synthesized via transmetalation from AgC_6_F_4_Br, and its Lewis acidity was assessed via DFT calculations and experimental methods, indicating an increased acidity compared to B(C_6_F_5_)_3_. The presence of bromine atoms is expected to facilitate structural analysis of Lewis acid‐base adducts of B(C_6_F_4_Br)_3_ by single‐crystal X‐ray diffraction in the future.

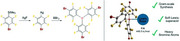

## 1 Introduction

The development and application of strong Lewis acids is a broad field of interest, with halogenated triarylboranes BR_3_ standing out due to their unique stability, acidity, and accessibility.^[^ ^1^ ^]^ Since the synthesis of the strong Lewis acid tris(pentafluorophenylborane) B(C_6_F_5_)_3_ in 1964,^[^ ^2^ ^]^ it has been widely used as a catalyst or stoichiometric reagent in organic reactions.^[^ ^3^, ^4^ ^]^ In inorganic chemistry, it is used as an abstraction reagent of anionic groups, (e.g., halides, H^‒^ or CH_3_^‒^),^[^ ^5^ ^]^ for the formation of (frustrated) Lewis acid‐base pairs^[^ ^6^, ^7^ ^]^ as well as for the synthesis of weakly coordinating anions, for example [B(C_6_F_5_)_4_]^‒^.^[^ ^8^, ^9^ ^]^ When coordinated to redox‐active molecules with Lewis basic donor sites, B(C_6_F_5_)_3_ strongly influences the redox properties of the corresponding species^[^ ^10^, ^11^ ^]^ which has proven useful in the context of doping organic polymers.^[^ ^12^, ^13^, ^14^, ^15^ ^]^

Over the last few decades, the search has been on for Lewis acids with even higher acidities.^[^ ^16^ ^]^ For borane Lewis acids, it was envisaged that different substituents could be introduced to phenyl boranes or that new electron‐withdrawing ligand motifs other than phenyl groups could be found. The Hammett constants describing the electron‐withdrawing character of different substituents bound to a phenyl group predict that chlorine or bromine (both *σ*_P _= 0.23) are more electron‐withdrawing than fluorine (*σ*_P _= 0.06) in the *para*‐position, while CF_3_ (*σ*_P _= 0.54) is even more so.^[^ ^17^, ^18^ ^]^ Based on this concept, Mitzel et al. synthesized the *para*‐substituted arylborane Lewis acids B(C_6_F_4_Cl)_3_^[^ ^19^ ^]^ and B(C_6_F_4_CF_3_)_3_.^[^ ^20^ ^]^ According to the Gutmann‐Beckett method, B(C_6_F_4_Cl)_3_ was found to be only slightly more acidic than B(C_6_F_5_)_3_ by 1%. However, the same method proved that B(C_6_F_4_CF_3_)_3_ was a much stronger Lewis acid, with an acidity that was 9% higher.^[^ ^19^, ^20^ ^]^ As Greb^[^ ^21^ ^]^ et al. considered B(C_6_F_5_)_3_ as the threshold for superacidity of soft Lewis acids, both Lewis acids B(C_6_F_4_Cl)_3_ and B(C_6_F_4_CF_3_)_3_ can be considered as soft Lewis superacids.

While most triarylboranes can be easily accessed by treating boron halides with phenyllithium^[^ ^2^ ^]^ or ‐magnesium^[^ ^22^ ^]^ compounds, some examples in the literature could only be synthesized via more sophisticated pathways.^[^ ^20^, ^23^ ^]^ For example, only the transmetalation of arylcopper reagents such as [CuC_6_F_4_CF_3_] with BBr_3_ was successful for B(C_6_F_4_CF_3_)_3_.^[^ ^20^ ^]^ Also, other routes toward borane Lewis acids have been described, such as the transmetalation from tin to boron to access tris(5,6,7,8‐tetrafluoronaphthalen‐2‐yl)borane.^[^ ^23^ ^]^

As a result of the chlorine substituent in the *para*‐position, B(C_6_F_4_Cl)_3_ is more Lewis acidic than B(C_6_F_5_)_3_. Our goal was to synthesize the bromine analogue B(C_6_F_4_Br)_3_ to enhance the acidity and, most importantly, to create a highly acidic, perhalogenated borane Lewis acid containing heavy bromine atoms. This is especially important when multiple BR_3_ moieties are to be coordinated to a central building block, leading to very large molecules consisting of only light atoms, making the characterization via single‐crystal X‐ray diffraction (scXRD) challenging.^[^ ^8^, ^10^, ^11^ ^]^ Furthermore, *para*‐brominated triarylborane Lewis acids B(C_6_Me_2_H_2_Br)_3_^[^ ^24^ ^]^ and tris(bromoduryl)borane^[^ ^25^ ^]^ B(C_6_Me_4_Br)_3_ are used as valuable building blocks to generate large, Lewis acidic, boron‐containing π‐systems, used as metal organic frameworks^[^ ^26^ ^]^ or fluorescent materials.^[^ ^27^ ^]^ The aimed B(C_6_F_4_Br)_3_ could therefore constitute a novel perfluorinated building block due to the reactivity of bromine substituents toward cross‐coupling reactions.

## 2 Results and Discussion

**Scheme 1:**
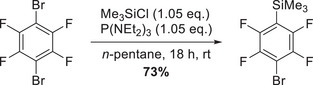
Synthesis of 1,4‐C_6_F_4_BrSiMe_3_ starting from 1,4‐C_6_F_4_Br_2_.

The synthesis of the Lewis acid B(C_6_F_4_Br)_3_ was initially approached via lithiation or magnesation of the starting material 1,4‐C_6_F_4_Br_2_. However, all attempts were unsuccessful, yielding complex product mixtures and only traces of the respective Lewis acid. Alternative approaches were therefore considered, including pathways involving the silylated compound 1,4‐C_6_F_4_BrSiMe_3_. This compound was obtained in 73% yield by silylation of 1,4‐C_6_F_4_Br_2_ according to a protocol of Bardin et al., using Me_3_SiCl and P(NEt_2_)_3_, followed by purification via distillation (Scheme 1).^[^ ^28^, ^29^ ^]^

Attempts to directly transmetalate from silicon to boron by reacting 1,4‐C_6_F_4_BrSiMe_3_ with boron trihalides showed no conversion. This led to the consideration of a so far unknown synthetic access to triarylboranes, starting from an organosilver compound. Perfluorinated AgC_6_F_5_ had been known for decades, and a more recent synthetic route allows for straightforward transmetalation from silicon to silver using C_6_F_5_SiMe_3_ and AgF.^[^ ^30^, ^31^ ^]^ Arylsilver compounds are used in general for transmetalation of aryl groups to various other metals. However, transmetalation from silver to boron, generating boron‐carbon bonds, is so far only known for silver cyanide,^[^ ^31^, ^32^ ^]^ not for the synthesis of arylboranes. We therefore aimed to investigate this synthetical access and followed a similar protocol to that described by Tyrra et al. to obtain AgC_6_F_4_Br by transmetalation from silicon to silver.^[^ ^30^ ^]^

**Scheme 2:**
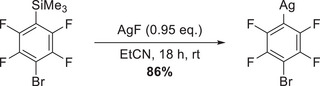
Synthesis of AgC_6_F_4_Br from 1,4‐C_6_F_4_BrSiMe_3_.

The reaction was carried out by treating a suspension of silver fluoride AgF in propionitrile (EtCN) with 1,4‐C_6_F_4_BrSiMe_3_, leaving the mixture stirring overnight in the dark, followed by filtration and evaporation of the solvent at elevated temperatures under reduced pressure (Scheme 2). The resulting greyish solid was washed with *n*‐pentane to remove any remaining starting material, and the crude product was dried for 48 hours to remove all traces of propionitrile.

After drying, unsolvated AgC_6_F_4_Br was obtained as a brownish powder that was analyzed by NMR and IR spectroscopy, as well as scXRD. Single crystals of [2(AgC_6_F_4_Br)_4_•7.46(toluene)] were obtained by heating a saturated solution of AgC_6_F_4_Br in toluene at 80 °C for 30 minutes, then slowly cooling it down to ‒70 °C after reaching ambient temperature.

**Figure 1:**
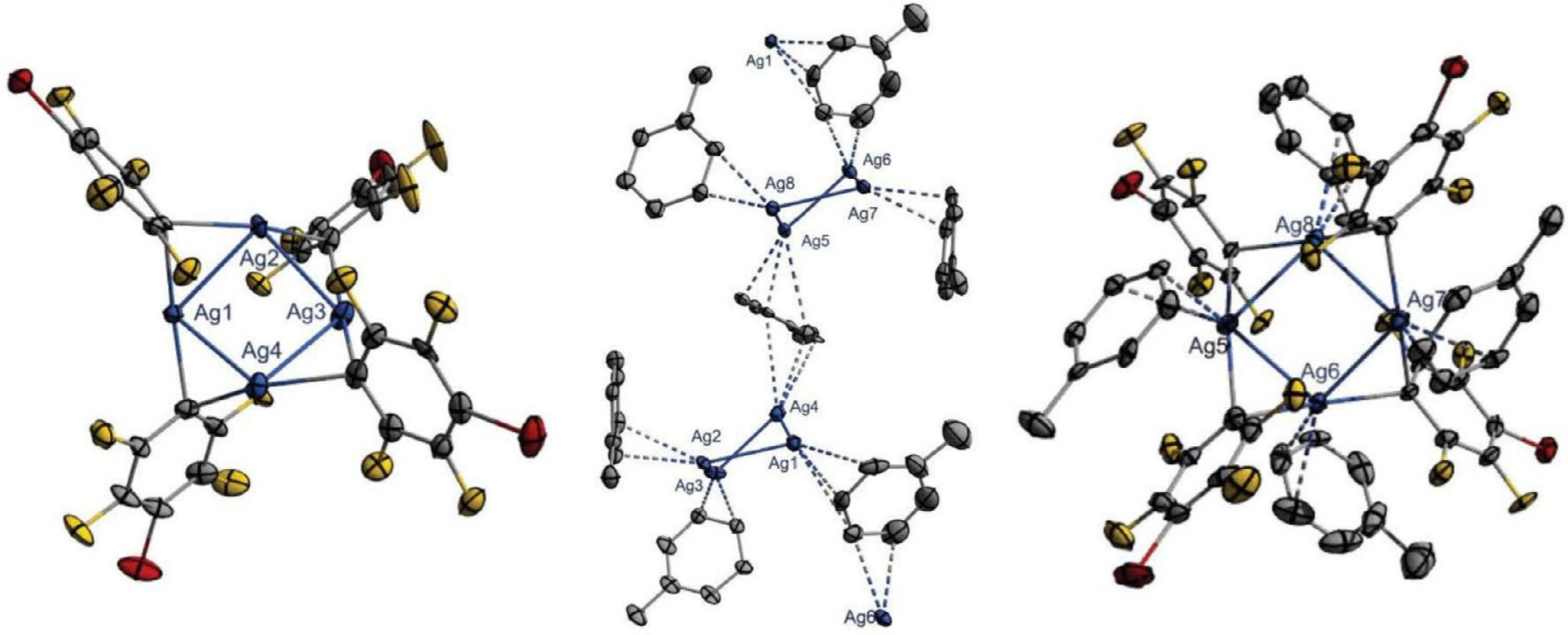
[AgC_6_F_4_Br]_4_ tetramer A (left, toluene molecules are omitted), both bridged tetramers A and B coordinated by toluene (middle, all other non‐coordinating toluenes, hydrogen atoms, and disordered toluene molecules are omitted for clarity), and tetramer B with coordinating toluene molecules (right, hydrogen atoms and disorder are omitted). Ellipsoids are depicted with a 50% probability level. Color code: blue ‐ silver, grey ‐ carbon, yellow ‐ fluorine, red ‐ bromine.

Several different crystal structures have been published of the related compound AgC_6_F_5_. Although the neat AgC_6_F_5_ has been shown to form a polymeric structure in the solid state, the [AgC_6_F_5_•*n*(arene)] solvates form tetrameric Ag_4_ units, each with a distinct Ag_4_ moiety.^[^ ^33^ ^]^ Eleven different [AgC_6_F_5_•*n*(arene)] structures have been described by Schulz et al., using ten differently substituted arene derivatives as solvates. Structures with *n *= 1, 2 or 4 coordinating arene molecules [AgC_6_F_5_•*n*(arene)] were obtained, where bridging arenes were found for the [AgC_6_F_5_•2(arene)] compounds with toluene and ethylbenzene. The exception of the compound [2(AgC_6_F_4_Br)_4_•7.46(toluene)] described herein is that it offers two distinct (AgC_6_F_4_Br)_4_ tetramer geometries and a total of 7.46 toluene molecules per asymmetric unit (Figure 1, Table 1). The average Ag‒Ag bond length of [2(AgC_6_F_4_Br)_4_•7.46(toluene)] is with *d_av_* = 2.765 Å in a similar range as the [AgC_6_F_5_•*n*(arene)] solvates in literature, where the smallest average bond length *d*_av_  =  2.761 Å is found for [AgC_6_F_5_•2(1,2,3,4,5‐C_6_Me_5_H)] and the largest for [AgC_6_F_5_•2(1,2,4‐C_6_Me_3_H_2_)] with *d*_av_  =  2.798 Å.^[^ ^33^ ^]^ The average bond length *d*_av_(Ag‒C_Phenyl_) of [2(AgC_6_F_4_Br)_4_•7.46(toluene)] with *d*_av _ = 2.248 Å is most similar to the literature compound [AgC_6_F_5_•4(1,2‐C_6_Me_2_H_4_)], solvated by four arene molecules, with *d*_av _= 2.247 Å.^[^ ^33^ ^]^ The Ag_4_‐ and the *ipso*‐C_4_‐units of the AgC_6_F_4_Br are both arranged in a puckered, sawhorse geometry. Several intermolecular interactions typical of arene‐solvated Ag_4_ tetramers are present in the solid‐state structure, including van der Waals interactions and hydrogen bonding.

**Table 1:** Selected structural data of [2(AgC_6_F_4_Br)_4_•7.46(toluene)] (*d* in Å).

	*d*	shortest ‒ longest
(Ag)_4_‐tetramers	Ag‒Ag	2.756(1) ‒ 2.773(1)
	C_Phenyl_‒Ag	2.209(9) ‒ 2.294(7)
π‐complexation	C_toluene_‒Ag	2.673(44) ‒ 3.355(39)
Halogen contacts	F‒F	2.619(11) ‒ 2.923(8)
	Br‒F	3.031(8) ‒ 3.289(8)

The most pronounced interaction is Ag‒C_toluene_ π‐complexation, with Ag‒C contacts present for all eight Ag atoms. Additionally, π‐stacking is observed in the structure, which can be found between the C_6_F_4_Br and silver‐coordinated toluene molecules, as well as between the different ligands themselves. Other intermolecular interactions include F‒H_toluene_ hydrogen bonding, halogen‐halogen contacts, and halogen‐carbon contacts (Table 1). A total of six toluene molecules is involved in Ag^+^ complexation for both ${\mathrm{Ag}}_{4^{-}}\text{tetramers}$, coordinating in an η^2^‐ or η^3^‐fashion (Figure 1, middle).

Although the hapticities and the bond lengths *d*(Ag‒C_toluene_) are similar to those of [AgC_6_F_5_•*n*(arene)] compounds reported in the literature, the coordination motifs differ. In the literature, the tetramers were coordinated by either one, two, or four arenes. Bridging arenes and therefore the formation of infinite Ag_4_ chains was only observed for some twofold‐coordinated Ag_4_ units [AgC_6_F_5_•2(arene)]. In the structure described herein, each [AgC_6_F_4_Br]_4_ tetramer is coordinated by a total of four toluene molecules. Two of these η^2^‐coordinate exclusively to one Ag atom of a distinct tetrameric unit (Ag3 and Ag2 for tetramer A, as well as Ag7 and Ag8 for tetramer B), while the other two arenes are placed between both tetrameric units, coordinating to one Ag atom of each tetramer via η^2^‐ or η^3^‐interactions in a bridging manner (Figure 1, middle). The tetramers are bridged via the toluene molecule T5 and the disordered molecules T1 and T2. Toluene T5 binds to Ag6 of tetramer B in an η^2^‐fashion and to Ag1 of tetramer A in an η^3^‐fashion via a total of four adjacent carbon atoms, one of which complexes both silver atoms, Ag1 and Ag6, directly. Both toluene molecules, T1 and T2, η^3^‐coordinate the silver atoms Ag4 and Ag5 of distinct tetrameric units via four adjacent carbon atoms, two of which complex both silver atoms directly. Due to their slightly different orientations, T1 interacts more strongly with Ag5, while T2 coordinates more strongly with Ag4 (T2 is omitted in Figure 1 for clarity). For other [AgC_6_F_5_•*n*(arene)] solvates, the bridged Ag atoms are described as being coordinated in an η^2^ or η^3^ manner via opposing carbon atoms of the bridging arene.^[^ ^33^ ^]^ Due to the two bridging toluenes T1/2 and T5, an infinite chain of Ag_4_ tetramers A and B is generated in the presented structure. This has been described in the literature, but only with one type of bridging arene in the asymmetric unit that bridges the same repeating Ag_4_‐unit.^[^ ^33^ ^]^

**Scheme 3:**
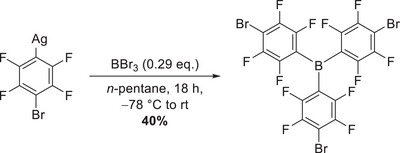
Synthesis of B(C_6_F_4_Br)_3_ by transmetalation from silver to boron.

The first attempts to synthesize B(C_6_F_4_Br)_3_ by transmetalation from silver to boron involved treating AgC_6_F_4_Br with boron halides BCl_3_ or BBr_3_ in CH_2_Cl_2_. Conversion of the starting material to the Lewis acid was observed; however, the reaction and purification were improved when using *n*‐pentane as a solvent, despite the low solubility of the starting material. The synthesis of B(C_6_F_4_Br)_3_ was therefore performed in *n*‐pentane by treating an excess of AgC_6_F_4_Br with BBr_3_ in *n*‐heptane at −78δ °C and leaving the suspension to stir overnight while warming up to room temperature (Scheme 3).

Reaction control was realized using ^19^F NMR spectroscopy in both *n*‐pentane and CH_2_Cl_2_ to ensure that the majority of the starting material had been consumed. The suspension was filtered to obtain a colorless solution. Evaporating the solvent gave a crude product containing several impurities, which are mainly borinic acid (C_6_F_4_Br)_2_BOH and 1,4‐C_6_F_4_BrH, most probably degradation products of the water adduct B(C_6_F_4_Br)_3_•H_2_O. While the benzene could be evaporated in a vacuum, the borinic acid appeared to have similar physical properties to the desired product. All attempts to purify the crude Lewis acid by sublimation remained unsuccessful, but the Lewis acid could finally be purified by two subsequent recrystallizations of the filtered reaction solution. The first recrystallization was carried out in a ‒24 °C freezer overnight, yielding B(C_6_F_4_Br)_3_ as a white crystalline solid with over 98% purity after drying in vacuum. The remaining *n‐*pentane filtrate was then used for the second recrystallization in a ‒70 °C freezer, yielding further B(C_6_F_4_Br)_3_ with approximately 5% borinic acid impurity. The first recrystallization at ‒24 °C yielded pure B(C_6_F_4_Br)_3_ in 30%, while the second recrystallization yielded additional an 10% of slightly impure B(C_6_F_4_Br)_3_.

**Figure 2:**
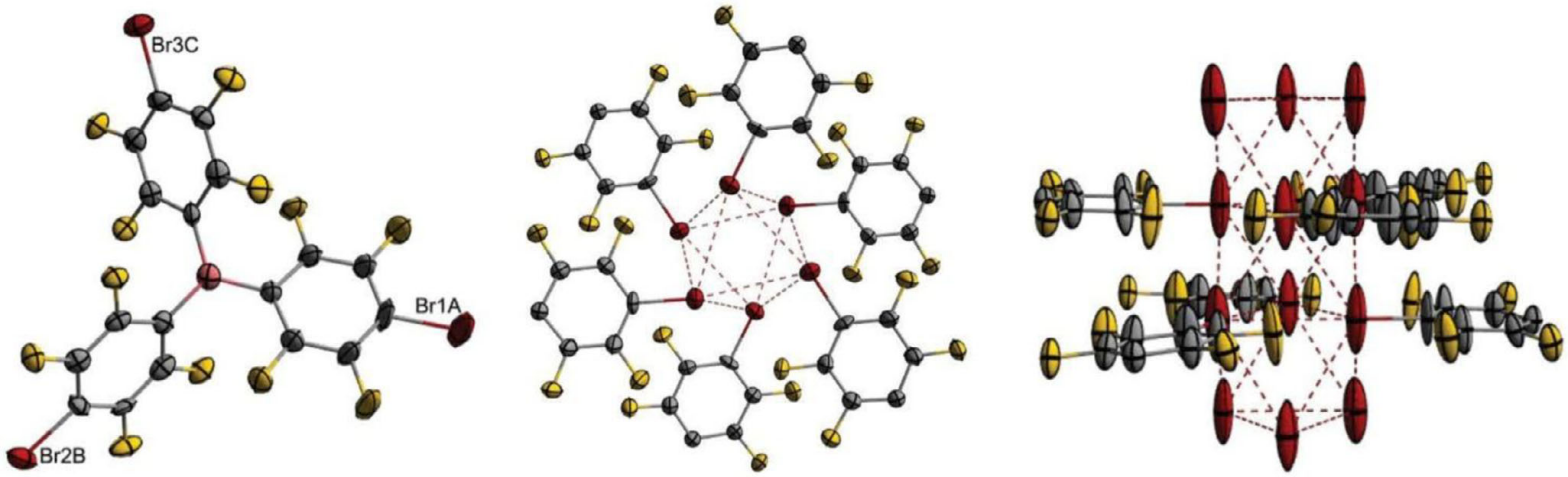
Molecular structure of B(C_6_F_4_Br)_3_ in the solid state (left) and intermolecular bromine‒bromine contacts between Br1A atoms (middle and right). Ellipsoids are depicted with a 50% probability level. Color code: pink ‐ boron, grey ‐ carbon, yellow ‐ fluorine, red ‐ bromine.

The structure identification of B(C_6_F_4_Br)_3_ was conducted using ^19^F, ^11^B, ^13^C, and 2D NMR spectroscopy, as well as IR spectroscopy, mass spectrometry, and scXRD analysis. The EI mass spectrum revealed the molecular mass [B(C_6_F_4_Br)_3_]^+^, and the ^11^B NMR analysis showed the characteristic broad signal at 59 ppm. Both fluorine signals were found in the ^19^F NMR spectrum, and all four carbon signals were visible in the measured ^13^C NMR spectrum (Table 2). All signals were assigned using 2D NMR methods (see Supporting Information) and all data are consistent with those of similar triarylboranes. Notable differences compared to B(C_6_F_5_)_3_ and B(C_6_F_4_Cl)_3_ are the low‐field‐shifted signal of the *meta*‐fluorine atoms and, therefore, a small chemical shift difference of Δ*δ*_F _ =  5 ppm in the ^19^F NMR spectrum, as well as the strongly high‐field‐shifted *para*‐carbon signal in the ^13^C NMR spectrum.^[^ ^19^, ^34^, ^35^ ^]^ These exceptions can both be explained by the heavy atom effect of the introduced bromine. The IR spectrum is in accordance with the calculated frequencies for B(C_6_F_4_Br)_3_ (see Supporting Information). By cooling a solution of B(C_6_F_4_Br)_3_ in *n‐*pentane, single crystals of the free Lewis acid were obtained and analyzed by scXRD. B(C_6_F_4_Br)_3_ crystallizes in the trigonal spacegroup $\;R\bar{3}$ with one molecule per asymmetric unit (Figure 2, left). The bond lengths *d*(B‒C) and *d*(C‒F) are similar to those of known triarylborane Lewis acids in the literature (Table 5).^[^ ^20^, ^34^, ^36^ ^]^ In the solid‐state structure, intermolecular interactions occur via C‒F, F‒F, Br‒F, and several Br‒Br contacts. Especially noteworthy are the interactions of atom Br1A, which forms not less than six Br‒Br contacts with other Br1A atoms (3.515(6)‐3.607(3) Å), as well as one Br‒F contact (Figure 2, middle and right). In each layer, three molecules are connected to each other and to the subsequent layer via bromine‐bromine contacts. The molecules in the second layer are rotated by 60° relative to the previous layer, resulting in the formation of a “bromine tunnel” due to these contacts. The other two bromine atoms, Br2B and Br3C, interact with each other (3.690(3) Å), and Br3C also exhibits two Br‒F contacts (3.284(9)‒3.291(8) Å). These halogen interactions are similar to those observed in other halogenated triarylboranes.^[^ ^36^, ^37^ ^]^

**Table 2:** Selected NMR chemical shifts of B(C_6_F_4_Br)_3_ in CD_2_Cl_2_ given in ppm.

	δ[^19^F NMR]			δ[^13^C NMR]			*δ*[^11^B NMR]
	F*_ortho_*	F*_meta_*	C*_ipso_*	C_ortho_	C*_meta_*	C*_para_*	
B(C_6_F_4_Br)_3_	−127.65	−132.65	118.0	148.1	145.5	107.6	59

The average torsion angle of B(C_6_F_4_Br)_3_ is 40.5°, which is substantially higher than the average torsion angle of 37.5° found for B(C_6_F_4_CF_3_)_3_.^[^ ^20^ ^]^ However, one of the C_6_F_4_Br‐groups, ring A, has a much smaller relative torsion angle of 30.0(2)° compared to the other two groups, which have angles of 45.2(1)° and 46.4(2)°. Remarkably, the bromine substituent Br1A of the least rotated ring A is the bromine atom with the greatest number of Br‒Br contacts. These favored interactions therefore seem to be a plausible explanation for the phenyl ring being less tilted in the solid state than the other two rings. Consequently, the observed torsion angles are more likely to be a packing effect. Comparing the torsion angles of the calculated structures of B(C_6_F_5_)_3_, B(C_6_F_4_Br)_3_, and B(C_6_F_4_CF_3_)_3_ in the gas phase shows that the average torsion angle increases as follows: B(C_6_F_5_)_3_ (38.7°), B(C_6_F_4_Br)_3_ (38.9°), and B(C_6_F_4_CF_3_)_3_ (39.7°). This trend aligns with the hypothesis that increasing torsion angles are associated with decreased π‐backbonding.^[^ ^20^ ^]^

**Figure 3:**
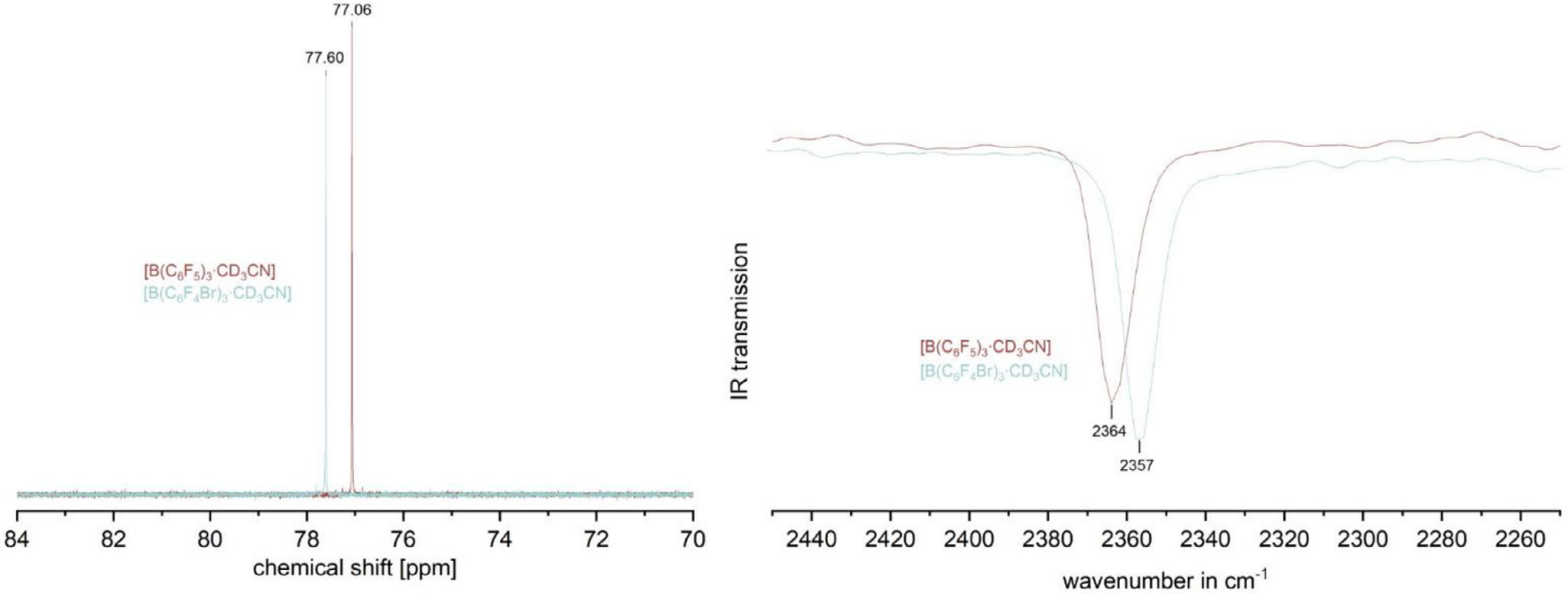
^31^P{^1^H} NMR spectra of B(C_6_F_4_Br)_3_•OPEt_3_ and B(C_6_F_5_)_3_•OPEt_3_ (left) and IR CN‐stretching bands of B(C_6_F_4_Br)_3_•CD_3_CN and B(C_6_F_5_)_3_•CD_3_CN.

The Lewis acidity of B(C_6_F_4_Br)_3_ was first evaluated theoretically and compared to analogous compounds with different *para*‐substituents B(C_6_F_4_H)_3_, B(C_6_F_5_)_3_, B(C_6_F_4_Cl)_3_, B(C_6_F_4_I)_3_, and B(C_6_F_4_CF_3_)_3_. Therefore, DFT calculations of the fluoride‐ and hydride ion affinities (FIA and HIA) were performed at the (*B3LYP‐D3BJ*/def2‐TZVPP) level of theory according to the method established by Greb et al. (Table 3).^[^ ^38^ ^]^ The resulting energies demonstrate that *para*‐substitution with heavier halides does indeed slightly increase the FIA and HIA compared to the well‐known Lewis acid B(C_6_F_5_)_3_, which can be attributed to the reduced π‐donation of heavier halides. This effect is most pronounced with bromine in the *para*‐position, resulting in an increased FIA of 5 kJ/mol and an increased HIA of 7 kJ/mol for B(C_6_F_4_Br)_3_ compared to the perfluorinated Lewis acid. These results are consistent with Hammett substituent constants for benzenes, in which *para*‐bromine has the highest *σ*_P_ value of 0.232, which is slightly higher than that for *para*‐Cl of 0.227.^[^ ^17^, ^18^ ^]^ The higher FIA of B(C_6_F_4_Br)_3_ compared to B(C_6_F_5_)_3_ classifies it as a soft Lewis superacid according to the definition stated by Greb et al.^[^ ^21^ ^]^ Furthermore, the calculations confirm B(C_6_F_4_CF_3_)_3_ as the strongest Lewis acid of this series, whereas B(C_6_F_4_H) is not a Lewis super acid. With the pure Lewis acid in hand, its acidity was further evaluated experimentally, using the Gutmann‐Beckett method, employing ^31^P NMR spectroscopy, as well as infrared analysis of the B(C_6_F_4_Br)_3_•CD_3_CN nitrile stretching bands *ṽ*(CN).^[^ ^21^, ^39^, ^40^, ^41^ ^]^ The ^31^P{^1^H} NMR shift of B(C_6_F_4_Br)_3_•OPEt_3_ was found to be slightly low‐field shifted compared to B(C_6_F_5_)_3_•OPEt_3_ by 0.54 ppm, which results in an acceptor number of AN = 80.9 (Figure 3, left, Table 4).

**Table 3:** Calculated FIAs and HIAs in kJ/mol (*B3LYP‐D3BJ*/def2‐TZVPP) of several *para*‐substituted halogenated triarylborane Lewis acids.

Lewis acid	FIA [kJ/mol]	HIA [kJ/mol]
B(C_6_F_4_H)_3_	422	453
B(C_6_F_5_)_3_	443.5	475
B(C_6_F_4_Cl)_3_	447	480
B(C_6_F_4_Br)_3_	448.5	482
B(C_6_F_4_I)_3_	445	479
B(C_6_F_4_CF_3_)_3_	492.5	529

**Table 4:** Acceptor number and ^31^P NMR shifts of B(C_6_F_4_Br)_3_, B(C_6_F_5_)_3_, and B(C_6_F_4_Cl)_3_ adducts with POEt_3_ in CH_2_Cl_2_ and infrared nitrile stretching bands *ṽ*(CN) of the adducts with CD_3_CN.

	B(C_6_F_4_Br)_3_•OPEt_3_	B(C_6_F_5_)_3_•OPEt_3_	B(C_6_F_4_Cl)_3_•OPEt_3_^[^ ^19^ ^]^
*δ*(^31^P)^[^ ^a^ ^]^	77.60	77.06	77.50
Δ*δ*(^31^P)^[^ ^b^ ^]^	36.6	36.06	36.5
AN^[^ ^c^ ^]^	80.9	79.7	80.7
Relative	102%	100%	101%
acidity	B(C_6_F_4_Br)_3_•CD_3_CN	B(C_6_F_5_)_3_•CD_3_CN	
*ṽ*(CN)^[^ ^d^ ^]^	2357	2364	−

^[a]^ *δ* in ppm.

^[b]^ Δ*δ* = [*δ*(Et_3_PO•LA) − *δ*(Et_3_PO)_hexane_].

^[c]^ AN = 2.21 · [*δ*(Et_3_PO•LA) − *δ*(Et_3_PO)_hexane_].

^[d]^ wavenumber in cm^−1^.

This indicates slightly increased Lewis acidity for the synthesized B(C_6_F_4_Br)_3_ compared to B(C_6_F_5_)_3_ and B(C_6_F_4_Cl)_3_, although the difference to the latter is negligible (Table 4). The measured infrared spectra of B(C_6_F_4_Br)_3_•CD_3_CN and B(C_6_F_5_)_3_•CD_3_CN reveal that the wavenumber of the nitrile group *ṽ*(CN) in the perfluorinated analogue is 7 cm^−1^ more blue‐shifted (Figure 3, right, Table 4). Therefore, this method suggests that the commercially available B(C_6_F_5_)_3_ has slightly higher Lewis acidity. According to Greb et al., distinct methods of scaling Lewis acidity often produce different rankings for Lewis acids of comparable strength.^[^ ^42^ ^]^

**Figure 4:**
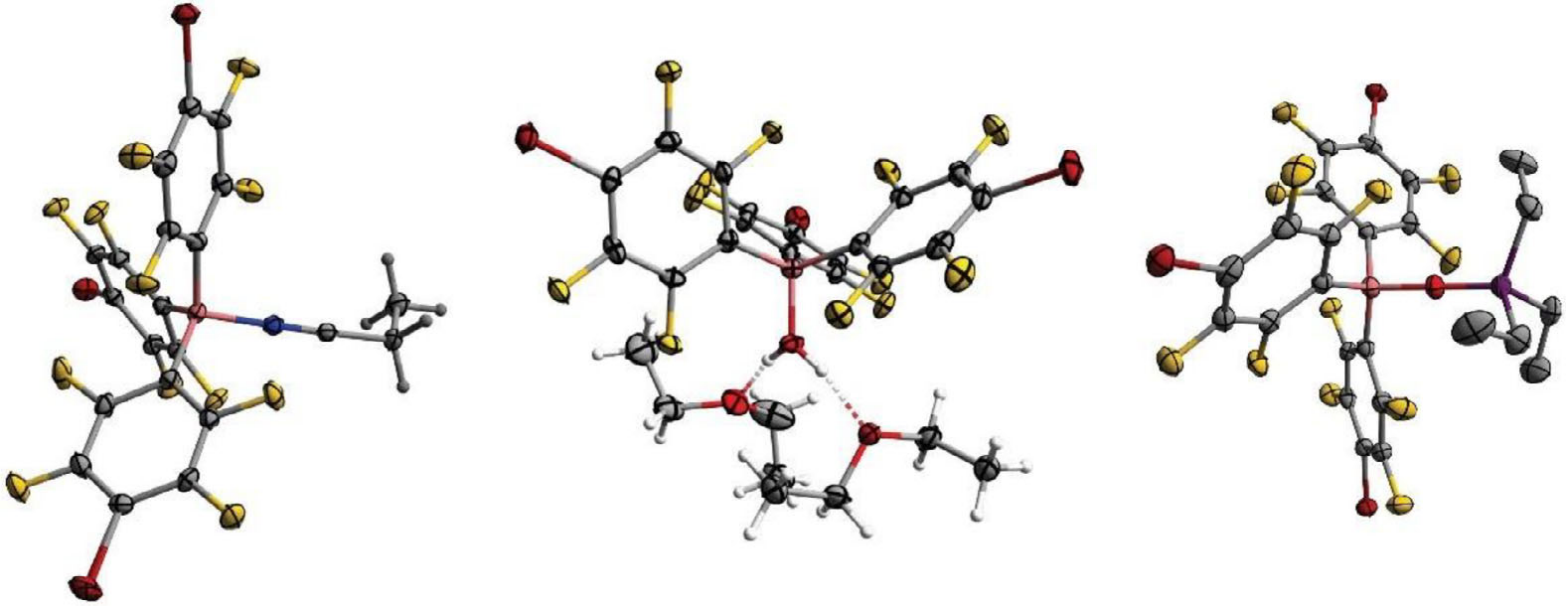
Molecular structure of [B(C_6_F_4_Br)_3_•EtCN], [B(C_6_F_4_Br)_3_•OH_2_•2 (Et_2_O)] and [B(C_6_F_4_Br)_3_•OPEt_3_] in the solid state. Ellipsoids are depicted with a 50% probability level, and hydrogen atoms were omitted for clarity [B(C_6_F_4_Br)_3_•OPEt_3_] (right). Color code: pink ‐ boron, grey ‐ carbon, yellow ‐ fluorine, red ‐bromine, blue ‐ nitrogen, red ‐ oxygen, violet ‒ phosphorus.

Further single crystals of three B(C_6_F_4_Br)_3_ adducts were obtained and analyzed using scXRD. Traces of EtCN in the starting material AgC_6_F_4_Br caused the formation and hence crystallization of the propionitrile‐adduct B(C_6_F_4_Br)_3_•EtCN, along with the free Lewis acid, from an *n*‐pentane solution by slowly cooling to −70 °C (Figure 4, left). Additionally, the water‐adduct B(C_6_F_4_Br)_3_•H_2_O was crystallized from an Et_2_O solution, and single crystals of the phosphine oxide‐adduct B(C_6_F_4_Br)_3_•OPEt_3_ were obtained from a CH_2_Cl_2_ solution, both by slow cooling to −70 °C (Figure 4, middle and right). All three Lewis acid‐base adducts have similar bond lengths *d*(B‒C) that are slightly longer than those of the free Lewis acid (Table 5). This is consistent with the bond lengths of similar adducts with the respective bases described in the literature.^[^ ^19^, ^43^, ^44^ ^]^ Also, the distances *d*(B‒L) between the Lewis acid and the base, as well as the nitrile and phosphine oxide bond lengths *d*(CN) and *d*(PO), are identical to those of literature‐known adducts (Table 5).^[^ ^19^, ^43^, ^44^ ^]^ The C‒O‒P unit is perfectly linear with an angle of 180°, while the B‒N‒C unit is almost linear with a slightly decreased angle of 177°. The intermolecular halogen contacts observed in the solid‐state structure of the free Lewis acid are also evident in these adducts. The EtCN adduct exhibits all types of Br‒F, Br‒Br, F‒F, C‒F, as well as halogen‐hydrogen contacts. In contrast, the H_2_O adduct offers no Br‒Br contacts but instead exhibits Br‒F and F‒F halogen interactions, as well as carbon Br‒C and F‒C contacts and F‒H hydrogen bonding.

**Table 5:** Selected structural data of B(C_6_F_4_Br)_3_ and (BrF_4_C_6_)_3_B•L (L = EtCN, H_2_O, OPEt_3_) (*d* in Å).

*d*	B(C_6_F_4_Br)_3_	B(C_6_F_4_Br)_3_•EtCN	B(C_6_F_4_Br)_3_•H_2_O	B(C_6_F_4_Br)_3_•OPEt_3_
B‒C	1.553(24)‒1.565(27)	1.626(3)‒1.640(3)	1.635(5)‒1.643(4)	1.637(3)
B‒L	‒	1.596(3)	1.539(4)	1.529(5)
PO/CN	‒	1.136(3)		1.502(3)
C‒F	1.322(15)‒1.372(17)	1.342(3)‒1.359(3)	1.344(4)‒1.360(3)	1.345(3)–1.354(3)
C‒Br	1.871(18)‒1.889(18)	1.850(5)‒1.878(2)	1.866(3)‒1.875(3)	1.854(5)

The POEt_3_ adduct is limited to Br‒Br and C‒F interactions, together with halogen‐hydrogen contacts. In comparison, the literature‐known compound B(C_6_F_4_Cl)_3_•POEt_3_ does not exhibit any intermolecular halogen but only halogen‐hydrogen contacts.^[^ ^19^ ^]^

## 3 Conclusion

We synthesized the novel aryl silver compound AgC_6_F_4_Br, and analyzed its tetrameric toluene solvate [2(AgC_6_F_4_Br)_4_•7.46(toluene)] using scXRD. The novel Lewis acid B(C_6_F_4_Br)_3_ was synthesized via transmetalation from silver to boron, a previously unreported approach for triarylboranes, with a yield of 40%. The Lewis acid was characterized using NMR and IR spectroscopy, and the crystal structures of the free B(C_6_F_4_Br)_3_, as well as three further adducts, were analyzed using scXRD. The Lewis acidity was computationally determined by calculating FIA and HIA and experimentally according to the Gutmann‐Beckett method, as well as by comparing the IR stretching of the nitrile adducts. An increased acidity of 2% compared to the acidity of the commercially available B(C_6_F_5_)_3_ was revealed, which characterizes B(C_6_F_4_Br)_3_ as a soft Lewis superacid. With the Lewis acid synthesized herein, the series of *para*‐substituted fluorinated triarylborane Lewis acids B(C_6_F_4_R)_3_ has been expanded, giving an even wider variety of strong Lewis (super‐)acids, with the acidity increasing in the order R = H, F, (I), Cl, Br, CF_3_. Due to the strong Lewis acidity and the presence of heavy bromine atoms, B(C_6_F_4_Br)_3_ may have advantages over B(C_6_F_5_)_3_ in the analysis of Lewis acid‐base adducts using scXRD.

## Supporting Information

The authors have cited additional references within the Supporting Information.^[^ ^45^, ^46^, ^47^, ^48^, ^49^, ^50^, ^51^, ^52^, ^53^, ^54^, ^55^, ^56^, ^57^, ^58^ ^]^

Deposition Number(s) 2469940 (for [2(AgC_6_F_4_Br)_4_•7.46(toluene)]), 2 469 941 (for B(C_6_F_4_Br)_3_), 2 469 942 (for [B(C_6_F_4_Br)_3_•(EtCN)]), 2 469 943 (for [B(C_6_F_4_Br)_3_•(H_2_O)•(Et_2_O)_2_]) and 2 469 944 (for [B(C_6_F_4_Br)_3_•OPEt_3_])</url > contain(s) the supplementary crystallographic data for this paper. These data are provided free of charge by the joint Cambridge Crystallographic Data Centre and Fachinformationszentrum Karlsruhe Access Structures service.

## Conflict of Interest

The authors declare no conflict of interest.

## Supporting information



Supporting Information

Supporting Information

Supporting Information

Supporting Information

Supporting Information

Supporting Information

## Data Availability

Data sharing is not applicable to this article as no new data were created or analyzed in this study.
