# Supplementary material for: Synthesis of the Soft Lewis Superacid Tris(4‐bromo‐2,3,5,6‐tetrafluorophenyl)borane B(C6F4Br)3 via C‒Ag to C‒B Transmetalation
Source: Chemistry. 2025 Sep 21;31(60):e02240. doi: 10.1002/chem.202502240 (PMC12559468; doi:10.1002/chem.202502240)
Supplement: Supplementary file 1 — Supporting Information [file CHEM-31-e02240-s002.pdf]

Supporting Information

Synthesis of the Soft Lewis Superacid Tris(4-bromo-2,3,5,6-tetrafluorophenyl)borane $B(C_6F_4Br)_3$ via C–Ag to C–B Transmetalation

Amina L. Moshtaha,[a] Erika M. Peter,[a] Robin Sievers,[a] Tim-Niclas Streit,[a] and Moritz Malischewski*[a]

---

[a] A. L. Moshtaha, E. M. Peter, R. Sievers, T.-N. Streit, Dr. M. Malischewski
Institute of Chemistry and Biochemistry – Inorganic Chemistry
Freie Universität Berlin
Fabeckstr. 34/36 14195, Berlin, Germany
E-mail: moritz.malischewski@fu-berlin.de

Content

General Information 2

Synthetic Procedures 4

Determination of Lewis acidity 8

NMR Spectra 10

IR Spectra 26

Mass Spectra 30

Quantum Chemical Calculations 32

Crystallographic Data 54

References 69

General Information

All Reactions and workups (except the workup of $C_6F_4BrSiMe_3$) were performed in previously heated glassware under an atmosphere of argon using standard Schlenk techniques and an oil pump vacuum of 10^{-3} mbar. Room temperature (rt) refers to 20 °C. The addition of liquid reagents and solvents was done by using threefold argon-flushed disposable syringes and septa, while solids were added in a glovebox with an atmosphere of argon. Low temperature reactions were performed in a cooled ethanol-bath. Glassware was cleaned by storing in a potassium hydroxide bath for several days, rinsed with diluted hydrochloric acid and doubly deionized water and dried at 150 °C.

Solvents and reagents

Anhydrous CH_2Cl_2 and *n*-pentane were obtained from the solvent system FMBRAUN MB SPS-800 and stored over activated 3 Å molecular sieves. Anhydrous EtCN was obtained by drying the purchased solvent with Sicapent®. The drying agent was added and the suspension was heated to reflux for 1 h. The solvent was then distilled and afterwards stored over activated 3 Å molecular sieves. Deuterated solvents CD_2Cl_2 and $CDCl_3$ were used as purchased and stored over activated 3 Å molecular sieves. All other solvents and commercially available reagents were used without further purification. Both, AgC_6F_4Br and $B(C_6F_4Br)_3$ were stored in a –30 °C freezer in a glovebox and AgC_6F_4Br was handled under exclusion of light.

Nuclear magnetic resonance (NMR) spectroscopy

^{1}H, ^{13}C, ^{19}F, ^{11}B and ^{31}P NMR spectra were recorded on a Bruker AVANCE III 700 (700 MHz) or JEOL ECX 400 (400 MHz) spectrometer by using 6 mm NMR glass tubes in deuterated solvents $CDCl_3$, CD_2Cl_2 and CD_3CN. All given chemical shifts in ^{1}H NMR spectra are calibrated on the resonance signals of $CDHCl_2$ contained in CD_2Cl_2 (δ = 5.32 ppm) or $CHCl_3$ contained in $CDCl_3$ (δ = 7.26 ppm).[45] The ^{13}C NMR spectra are calibrated on the respective resonance signals of CD_2Cl_2 (δ = 53.84 ppm) or CD_3CN (δ = 53.84 ppm).[45] The ^{19}F, ^{11}B and ^{31}P NMR spectra are device-internally calibrated relative to the resonance signal of $CFCl_3$, $BF_3 \cdot OEt_2$ and H_3PO_4 according to the unified chemical shift scale.[46] All chemical shifts (δ) are given in parts per million (ppm), the coupling constants J in Hz and the signals are specified according to the multiplicity (s = singlet, d = doublet, t = triplet, q = quartet, m = multiplet, br = broad). The program MestReNova Version 14.3.0 was used to evaluate and plot the data.[47]

Infrared (IR) spectroscopy

Infrared spectra were measured using a Thermo-Scientific Nicolet iS10 FTIR spectrometer with DuraSamplIR accessory in attenuated total reflection at room temperature or on a Bruker ALPHA FT-IR spectrometer inside a glovebox equipped with a diamond ATR attachment. The software Origin 2024 was used to plot the data.[48]

Mass spectrometry (MS)

Mass spectra were recorded using a VARIAN MAT 711 spectrometer by electron impact ionization (EI) at the department of mass spectroscopy at the Freie Universität Berlin. A detailed listing of fragmentation is dispensed, instead the molecular ion peak or a characteristic fragment peak is stated.

X-Ray diffraction (XRD)

X-Ray data was collected on a BRUKER D8 Venture system. Data was collected at 100(2) K using graphite-monochromated Mo K_{α} (λ_{α} = 0.71073 Å) or Cu K_{α} radiation (λ_{α} = 1.54178 Å). The strategy for the data collection was evaluated by using the Smart software. The data were collected by the standard "*ψ*-*ω* scan techniques" and were scaled and reduced using Saint+software. The structures were solved by using Olex2[49] with the XT[50] structure solution program using Intrinsic Phasing and refined with the XL refinement package[51,52] using Least Squares minimization. Bond lengths and angles were measured with Diamond Crystal and Molecular Structure Visualization Version 5.0.2. [53] Drawings were generated with POV-Ray. [54] The crystal structures discussed in the manuscript have been reported as CSD Communications (deposition numbers CCDC 2469940-2469944). These data are provided free of charge by the joint Cambridge Crystallographic Data Centre and Fachinformationszentrum Karlsruhe Access Structures service www.ccdc.cam.ac.uk/structures.

Quantum chemical calculations

DFT calculations were performed with Gaussian 16.[55] Structure optimizations and frequency calculations were done using *B3LYP-D3BJ*/def2-TZVPP. Solutions were processed using Avogadro[56] and Chemcraft 1.8.[57] Calculated frequencies were multiplied with the scaling factor 0.9657.[58]

Synthetic Procedures

(4-Bromo-2,3,5,6-tetrafluorophenyl)silane[28,29]

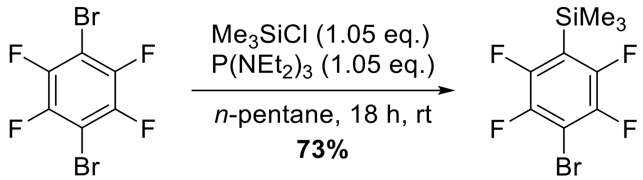

In a 500 mL 2-neck Schlenk flask, equipped with a dropping funnel, 1,4-$C_6F_4Br_2$ (19.0 g, 61.7 mmol, 1.0 eq.) was dissolved in dry *n*-pentane (160 mL) and trimethylsilyl chloride Me_3SiCl (7.04 g, 64.8 mmol, 8.19 mL, 1.05 eq.) was slowly added to the solution. Dry *n*-pentane (30 mL) was added to the dropping funnel, in which $P(NEt_2)_3$ (16.0 g, 64.8 mmol, 17.8 mL, 1.05 eq.) was dissolved. The $P(NEt_2)_3$-solution was slowly added to the flask over the course of 15 min, while a suspension from first red to yellowish/white was obtained. The reaction mixture was stirred overnight (18 h) and the conversion was controlled *via* ^{19}F NMR spectroscopy in *n*-pentane. The white suspension was filtered and the flask, as well as the remaining solid were washed with *n*-pentane (ca. 150 mL). The obtained solution was evaporated under reduced pressure (100 mbar), resulting in new precipitation of a white solid. The suspension was again filtered and the flask was again washed with a small amount of *n*-pentane. After evaporation of the solvent, a brown oil (18.05 g) was obtained as the crude product, which also contained remaining starting material 1,4-$C_6F_4Br_2$ and disilylated product 1,4-$C_6F_4(SiMe_3)_2$. The crude product was distilled (17 mbar, 120 °C), for purification from remaining $P(NEt_2)_3$ byproducts. Two fractions of colourless oil were obtained, both containing mainly the desired product, but also some amount of the starting material 1,4-$C_6F_4Br_2$ and disilylated product 1,4-$C_6F_4(SiMe_3)_2$. The first fraction (2.72 g) contained 85.5% of the product while the second fraction (11.89 g) consisted of 94% product, which yields 1,4-$C_6F_4BrSiMe_3$ (13.61 g, 45.2 mmol) in total in approximately 73% yield.

^{19}F NMR (CD_2Cl_2, rt, 375.79 MHz): δ = –126.80 - –126.92 (m, 2 F, *ortho*-F), –134.59 - –134.72 (m, 2 F, *meta*-F) ppm. **^{1}H NMR** (CD_2Cl_2, rt, 400 MHz): δ = 0.41 - 0.39 (m, 9 H, TMS) ppm. The analytical data are consistent with those reported in literature. [28,29]

The reaction proceeded more selective in smaller scales by using less than 10 g of the starting material 1,4-$C_6F_4Br_2$. Here, no disilylated product and only traces of unreacted starting material were present in the crude product. If there was starting material and disilylated product 1,4-$C_6F_4(SiMe_3)_2$ present in the crude product, there was no selective purification method (recrystallisation, column chromatography or distillation) to obtain pure 1,4-$C_6F_4BrSiMe_3$.

In general, these impurities do not interfere in the subsequent reaction with AgF and the following product AgC_6F_4Br is easier purified from those compounds by washing with *n*-pentane. In either case, regardless of how clean the conversion was, it is highly recommended to distil the crude product, otherwise there are remaining traces of impurities visible in the ^{1}H NMR spectrum. These impurities can lead to impurities of the Lewis acid in the following reactions.

(4-Bromo-2,3,5,6-tetrafluorophenyl)silver

The reaction and work up was conducted under exclusion of light.

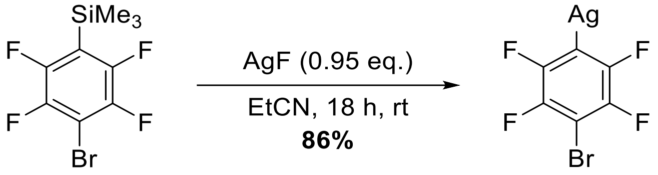

AgF (3.202 g, 25.24 mmol, 0.95 eq.) was added to a 250 mL Schlenk flask and was suspended in dry EtCN (100 mL). While stirring, $C_6F_4Br(SiMe_3)$ (8.00 g, 26.57 mmol, 1.0 mmol) was slowly added to the suspension. The reaction was allowed to stir over night (18 h), after which reaction control was realised *via* ^{19}F NMR spectroscopy in EtCN, showing only traces of remaining starting material**.** The brown suspension was filtered over a frit (porosity: 4) and the obtained solution was evaporated under reduced pressure (10^{-3} mbar) at 60 °C, resulting in a crystalline white/greyish solid. The solid was washed with dry *n*-pentane (2 x 20 mL). The product was then dried at 80 °C under reduced pressure (10^{-3} mbar) for almost 48 h to yield AgC_6F_4Br (7.25 g, 21.6 mmol, 86%) as a light brownish powder. Single crystals of $[2(AgC_6F_4Br)_4•7.46(toluene)]$ for scXRD analysis were obtained from a saturated solution of AgC_6F_4Br in toluene that was heated to 80 °C for 30 minutes, allowed to reach room temperature and then slowly cooled down in a –70 °C freezer.

^{19}F NMR (CD_2Cl_2, rt, 375.79 MHz): δ = –100.64 - –100.80 (m, 2 F, *ortho*-F), –131.09 - –131.18 (m, 2 F, *meta*-F) ppm. **^{19}F NMR** (CD_3CN, rt, 375.79 MHz): δ = –105.84 - –106.03 (m, 2 F, *ortho*-F), –136.17 - –136.36 (m, 2 F, *meta*-F) ppm. **^{13}C NMR** (CD_3CN, rt, 100.42 MHz): δ = 150.7 (*ortho*-CF), 144.4 (*meta*-CF), 130.5 (C–Ag), 96.6 (C–Br) ppm. **FT-IR** (ATR) $\tilde{\nu}$ = 1611, 1573, 1531, 1438, 1421, 1375, 1271, 1031, 979, 932, 873, 846, 739, 709, 559 cm^{-1}. **IR** (calculated) $\tilde{\nu}$ = 1422, 1398, 1327, 1179, 921, 856, 727, 561 cm^{-1}.

The long drying process is crucial to obtain the non-coordinated, solvent free AgC_6F_4Br with no traces of EtCN left. Remaining EtCN is not obviously recognizable, as the drying process did not show a visible difference in the infrared spectra, and only traces of EtCN were visible in the ^{1}H NMR spectrum, not anticipating an amount interfering later on. In fact, without drying for more than 24 h, substantial amounts of solvent adducts with EtCN will be obtained during the synthesis of the Lewis acid, underlined by the accidentally obtained crystal structure of $B(C_6F_4Br)_3•EtCN$.

The arylsilver is poorly soluble in non-coordinating solvents like dichloromethane, but sufficiently to measure a ^{19}F NMR spectrum of the free AgC_6F_4Br. For the measurement of a ^{13}C NMR spectrum, a donor solvent with higher polarity, CD_3CN was used. The signals in the ^{19}F and ^{13}C NMR spectra were assigned using 2D NMR methods HMQC and HMBC.

Tris(4-bromo-2,3,5,6-tetrafluorophenyl)borane

The reaction was conducted under exclusion of light.

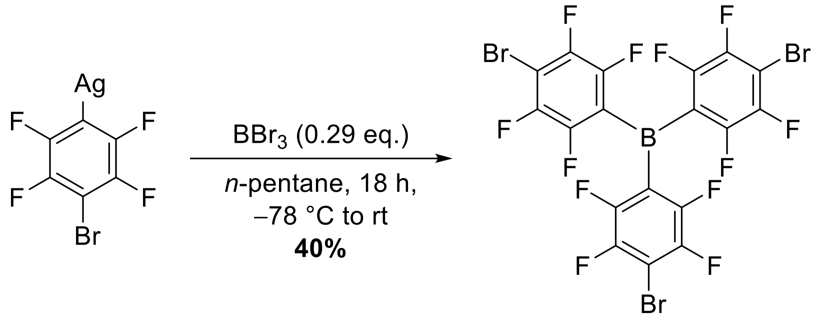

AgC_6F_4Br (7.00 g, 20.84 mmol, 1.0 eq.) was placed in a 500 mL Schlenk flask and was suspended in dry *n*-pentane (130 mL). The suspension was cooled to –78 °C, and under stirring the BBr_3-solution (1 M in *n*-heptane, 6.00 mmol, 6.00 mL, 0.29 eq.) was added dropwise. The reaction was stirred and allowed to warm up to room temperature overnight (18 h). Reaction control was realised *via* ^{19}F and ^{11}B NMR spectroscopy in *n*-pentane and CH_2Cl_2, showing complete conversion. The white suspension was filtered over a frit and the obtained solution was cooled down in a –24 °C freezer for recrystallization. The solid left from the filtration was not washed with *n*-pentane. There was some product left behind, but further washing brought also more impurities. After 24 h, the solution was transferred to another Schlenk flask and the precipitated solid was dried under reduced pressure. The product $B(C_6F_4Br)_3$ was obtained as a white solid (1.240 g, 1.785 mmol, 30%) and was stored in a –30 °C freezer in a glove box. The remaining solution from the first recrystallization was cooled down for a second recrystallization in a –73°C freezer. The product (400 mg, 0.576 mmol, 10%) obtained from the second recrystallization showed ca. 5% of the borinic acid byproduct $(C_6F_4Br)_2BOH$.

Single crystals for scXRD analysis of $B(C_6F_4Br)_3$ and $(C_6F_4Br)_3B$•EtCN were obtained from a solution in *n*-pentane by slowly cooling down to –73 °C.

Single crystals for scXRD analysis of $(C_6F_4Br)_3B$•H_2O were obtained from a solution in Et_2O by slowly cooling down to –73 °C, while single crystals of $(C_6F_4Br)_3B$•$OPEt_3$ were obtained from a solution in CH_2Cl_2.

^{19}F NMR (CD_2Cl_2, rt, 375.79 MHz): δ = –127.64 - –127.67 (m, 6 F, *ortho*-F), –132.61 - –132.70 (m, 6 F, *meta*-F) ppm. **^{13}C NMR** (CD_2Cl_2, rt, 100.42 MHz): δ = 148.1 (*ortho*-C_6F_4Br), 145.5 (*meta*-C_6F_4Br), 118.0 (**C**–B), 107.6 (**C**–Br) ppm. **^{11}B NMR** (CD_2Cl_2, rt, 128.15 MHz): δ = 59 (broad) ppm. **FT-IR** (ATR) $\tilde{\nu}$ = 1628 (m), 1581 (w), 1443 (s), 1370 (m), 1294 (m), 1092 (m), 949 (s), 843 (m), 755 (m), 657 (m) cm^{-1}. **IR** (calculated) $\tilde{\nu}$ = 1596, 1547, 1435, 1346, 1265, 1075, 947, 825, 747, 648, 590 cm^{-1}. **Mass** (EI, positive) *m/z* for $[B(C_6F_4Br)_3]^+$ calculated: 693.7431; measured: 693.7499.

^{19}F NMR signals were assigned via $^{19}F/^{13}C$ HMBC and HMQC 2D NMR experiments. ^{19}F NMR signals of the *ortho*-fluorine showed coupling to the boron bound carbon C-B, while the *meta*-fluorine atoms showed coupling to the bromine-bound carbon C-Br.

Bis(4-bromo-2,3,5,6-tetrafluorophenyl)(hydroxy)borane

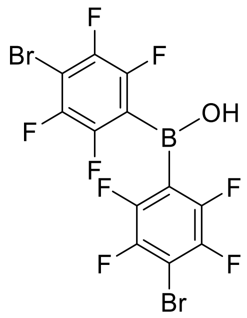

The borinic acid $(C_6F_4Br)_2BOH$ is always formed in small amounts during the synthesis of the Lewis acid $B(C_6F_4Br)_3$ which was not possible to suppress so far. After the first recrystallization of the crude product, at –24 °C, the Lewis acid is obtained pure, while the second recrystallization conducted at –73 °C yields the desired product with 5% of borinic acid $(C_6F_4Br)_2BOH$ as an impurity. The borinic acid was assigned based on the coupling of the ^{1}H NMR signal at 7.44 ppm to the *ortho*-fluorine atom at –132.16 ppm in the ^{1}H,^{19}F HETCORE NMR experiment. Furthermore, it seems that there are stoichiometric amounts of water bound to the borinic acid, which can be seen in the ^{1}H NMR spectrum at 6.83 ppm. This signal does not couple to any carbon or fluorine in 2D NMR experiments, while the signal couples to the borinic acid proton at 7.44 ppm in the NOESY NMR experiment. Furthermore, the water seems to slowly react, most probably with the Lewis acid, yielding protonated benzene C_6F_4BrH and more of the borinic acid. Also, the borinic acid seems to degrade with time by condensing to the corresponding anhydride $(C_6F_4Br)_2BOB(C_6F_4Br)_2$, leading to a new set of ^{19}F NMR signals.

^{1}H NMR (CD_2Cl_2, rt, 400 MHz): δ = 7.44 (s, 1 H, R_2BOH), 6.83 (s, 2 H, H_2O) ppm. **^{19}F NMR** (CD_2Cl_2, rt, 375.79 MHz): δ = –132.11 - –132.21 (m, 2 F, *ortho*-F), –133.17 - –133.26 (m, 2 F, *meta*-F) ppm. **^{11}B NMR** (CD_2Cl_2, rt, 128.14 MHz): δ = 40 ppm.

FT-IR (ATR) $\tilde{\nu}$ = 3639 (vw), 3527 (vw), 3474 (vw) cm^{-1}. **IR** (calculated) $\tilde{\nu}$ = 3769 ($R_2BOH \cdot H_2O$), 3704 (R_2BOH), 3369 ($R_2BOH \cdot H_2O$) cm^{-1}. **Mass** (EI, positive) *m/z* for $[HOB(C_6F_4Br)_2]^+$ calculated: 483.8339; measured: 483.8397.

Determination of Lewis acidity

Gutmann-Beckett

Triethylphosphine oxide $POEt_3$ (5.0 mg, 0.037 mmol, 1 eq.) and the corresponding Lewis acid $B(C_6F_4Br)_3$ (26 mg, 0.037 mmol, 1 eq.) or $B(C_6F_5)_3$ (19 mg, 0.037 mmol, 1 eq.) were placed in a Schlenk-tube and dissolved in 1 mL of CD_2Cl_2. After a few minutes the solutions were transferred to a Young NMR-tube and ^{11}B, ^{19}F, ^{1}H and $^{31}P\{^{1}H\}$ NMR-spectra were measured.

The acceptor number AN of the Lewis acid according to the Gutmann Beckett method was calculated by the formula AN = 2.21 · ($\delta_{LA•POEt3}$(^{31}P) – 41), where the ^{31}P NMR shift of uncoordinated $POEt_3$ equals δ = 41 ppm.[40]

$B(C_6F_4Br)_3$•$POEt_3$

^{19}F NMR (CD_2Cl_2, rt, 375.79 MHz): δ = –132.63 - –132.70 (m, 6 F, *ortho*-F), –136.85 - –136.93 (m, 6 F, *meta*-F) ppm. **^{31}P NMR{^{1}H}** (CD_2Cl_2, rt, 161.7 MHz): δ = 77.60 ppm. **^{11}B NMR** (CD_2Cl_2, rt, 128.14 MHz): δ = –2.4 ppm. **AN** = 80.9

$B(C_6F_5)_3$•$POEt_3$

^{19}F NMR (CD_2Cl_2, rt, 375.79 MHz): δ = –134.37 - –134.46 (m, 6 F, *ortho*-F), –158.98 - –159.09 (m, 3 F, *para*-F), –164.94 - –165.03 (m, 6 F, *meta*-F) ppm. **^{31}P NMR{^{1}H}** (CD_2Cl_2, rt, 161.7 MHz): δ = 77.06 ppm. **^{11}B NMR** (CD_2Cl_2, rt, 128.14 MHz): δ = –2.5 ppm. **AN** = 79.7

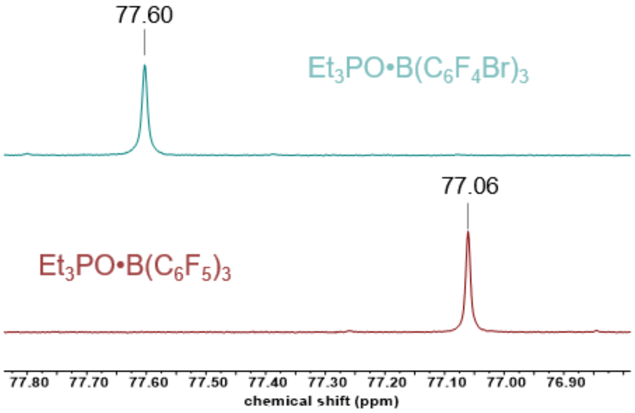

Figure S1. $^{31}P\{^{1}H\}$ NMR-spectra of $B(C_6F_4Br)_3$ (top) and $B(C_6F_5)_3$ (bottom) adducts with $POEt_3$.

Vibrational analysis of CD_3CN adduct

The respective Lewis acid (20 mg) was placed in a Schlenk-tube and 0.5 mL of CD_3CN were added. After stirring the solutions for a few minutes, the solvent was evaporated under reduced pressure and IR-spectra of the white solids were measured.

$[B(C_6F_4Br)_3{\bullet}CD_3CN]$ $\tilde{\nu}$ (IR, ATR powder) = 2357 cm^{-1}.
$[B(C_6F_5)_3{\bullet}CD_3CN]$ $\tilde{\nu}$ (IR, ATR powder) = 2364 cm^{-1}.

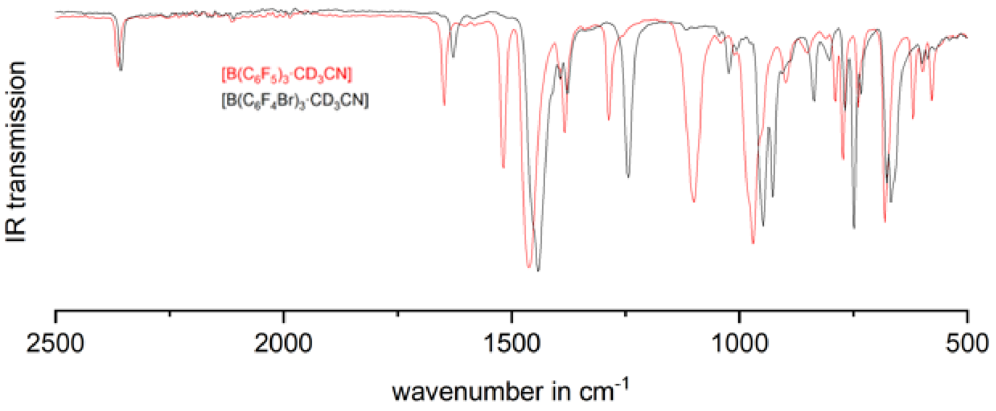

Figure S2. Layered IR (ATR) spectra of the CD_3CN adducts of the Lewis acids $B(C_6F_4Br)_3$ (black) and $B(C_6F_5)_3$ (red).

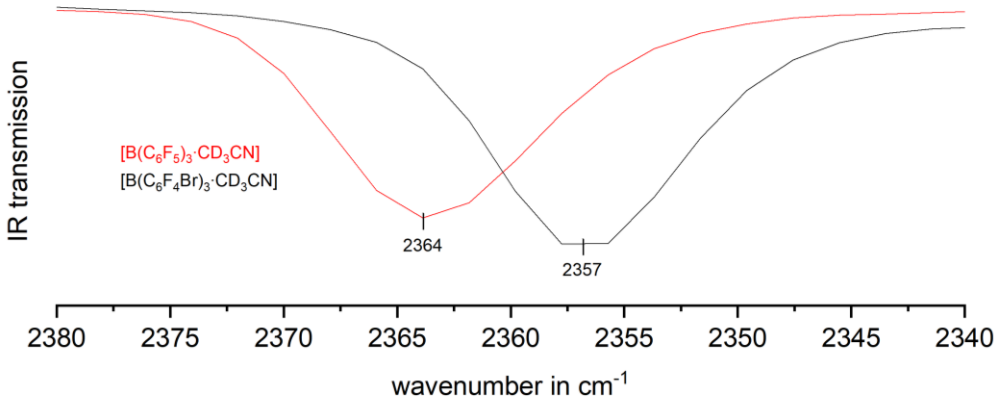

Figure S3. CN-stretching bands of the IR (ATR) spectra of CD_3CN adducts of the Lewis acids $B(C_6F_4Br)_3$ (black) and $B(C_6F_5)_3$ (red).

NMR Spectra

(4-Bromo-2,3,5,6-tetrafluorophenyl)silane

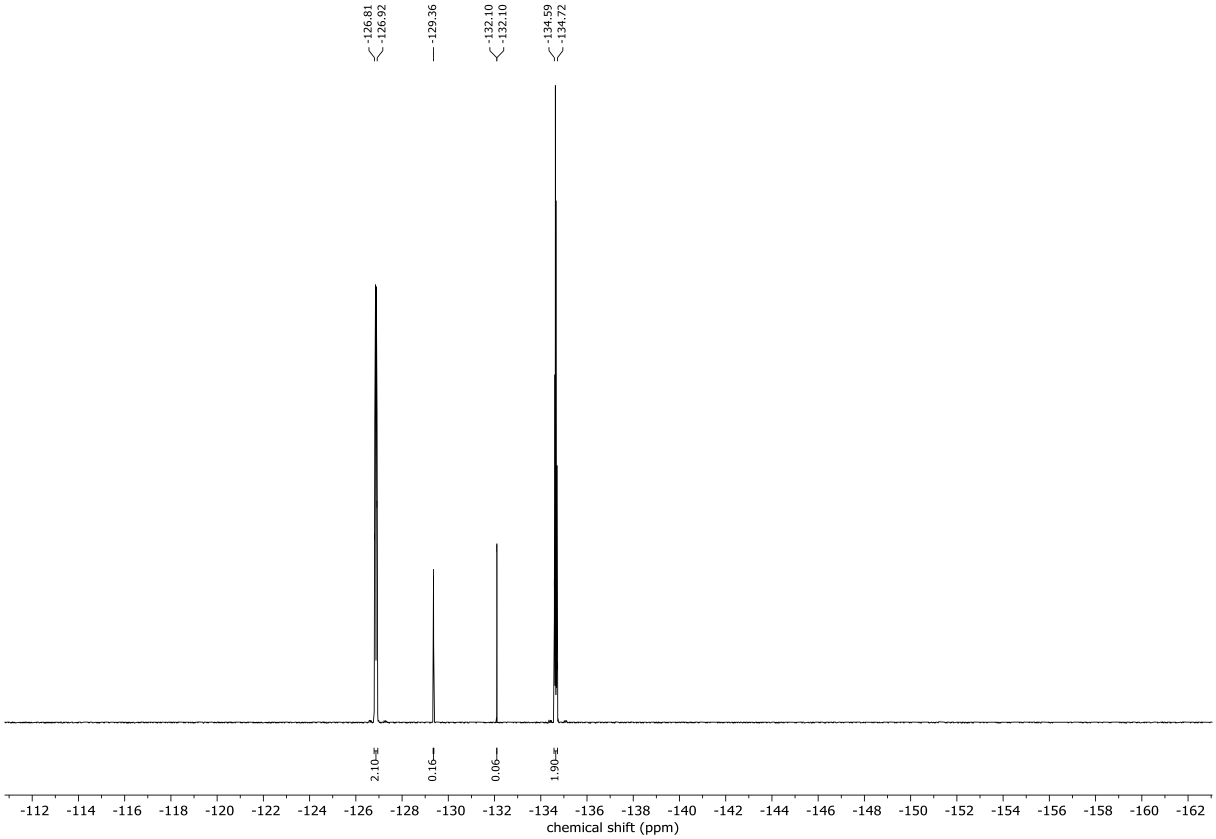

Figure S4. ^{19}F NMR (CD_2Cl_2, rt, 375.79 MHz) spectrum of $C_6F_4BrSiMe_3$.

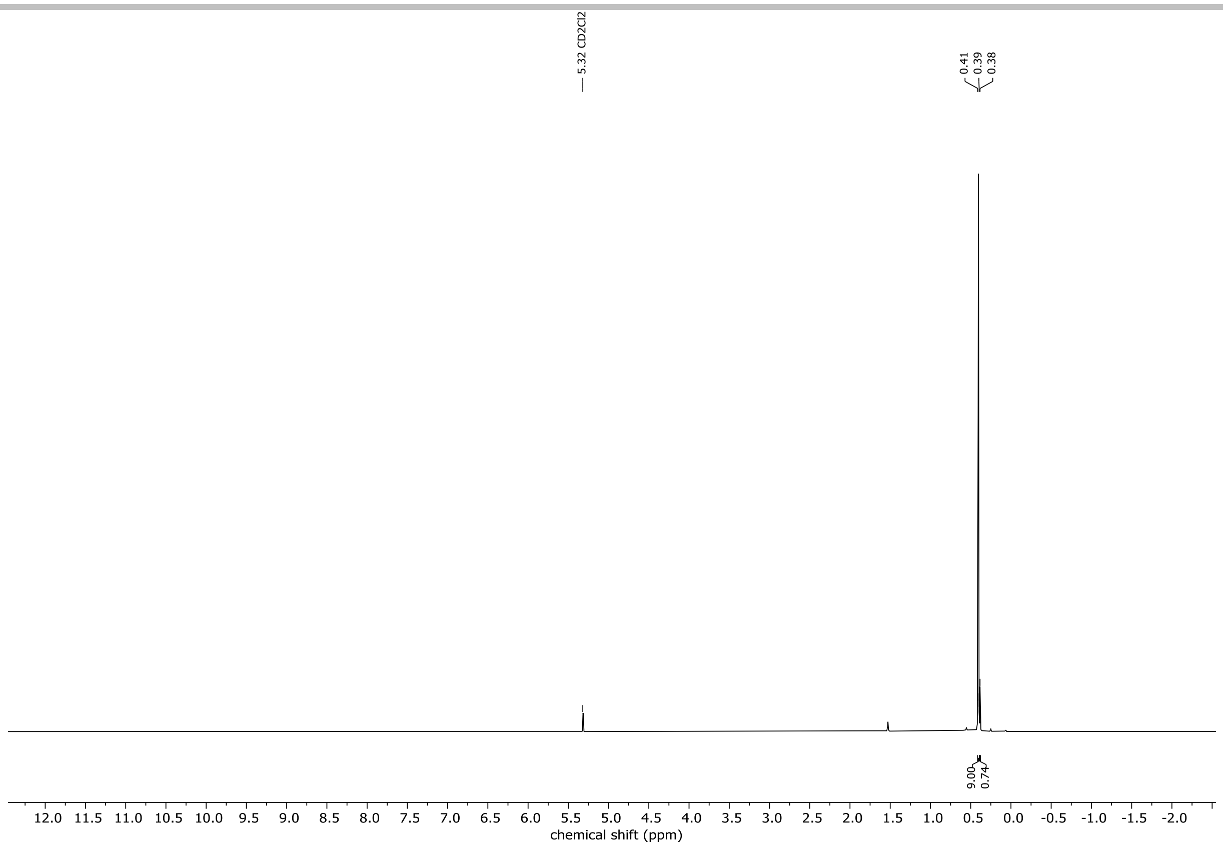

Figure S5. ^{1}H NMR (CD_2Cl_2, rt, 400 MHz) spectrum of $C_6F_4BrSiMe_3$.

(4-Bromo-2,3,5,6-tetrafluorophenyl)silver

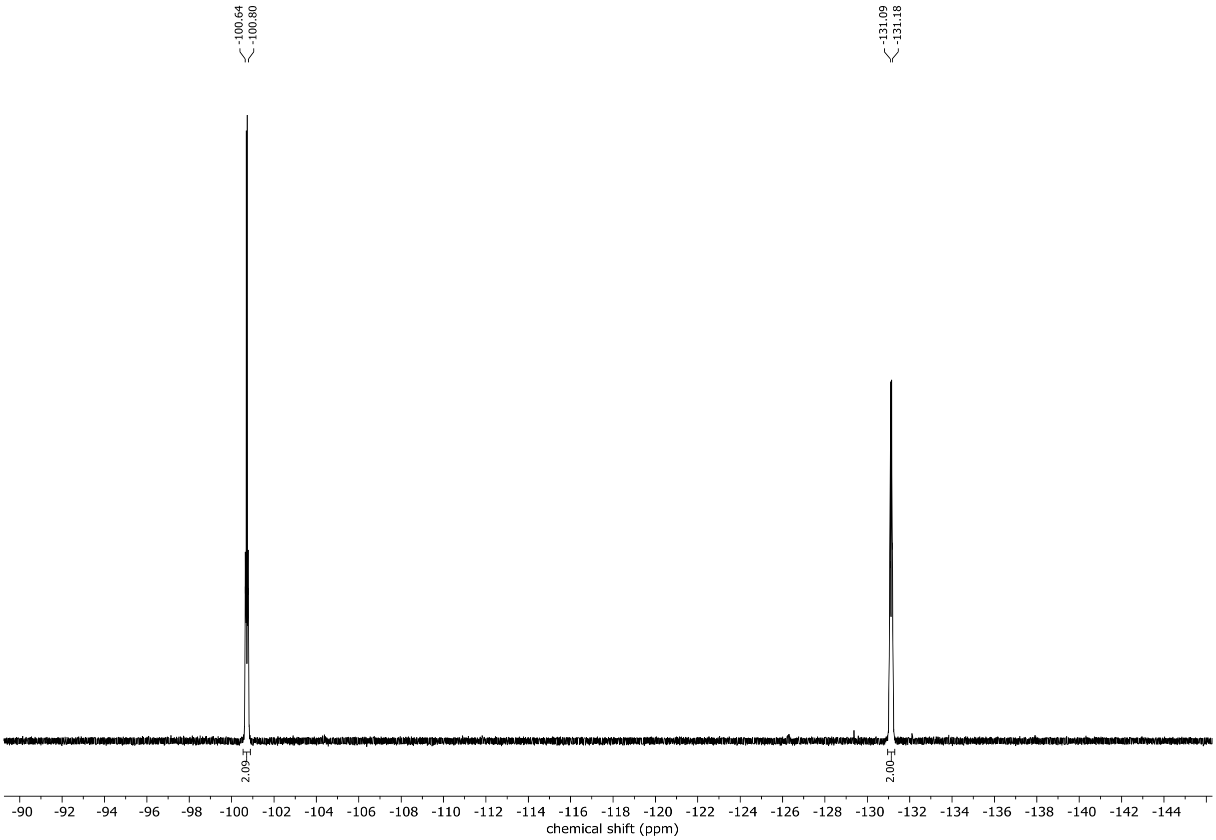

Figure S6. ^{19}F NMR (CD_2Cl_2, rt, 375.79 MHz) spectrum of AgC_6F_4Br.

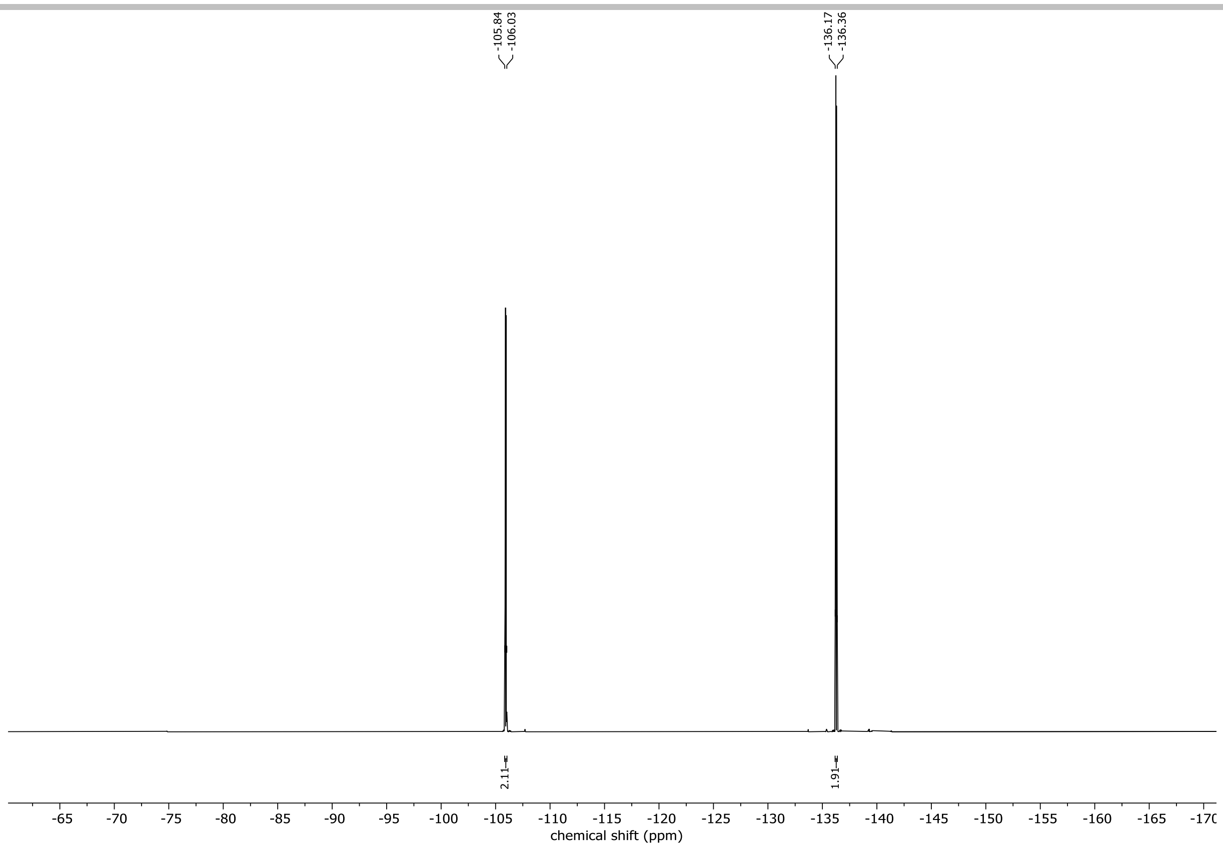

Figure S7. ^{19}F NMR (CD_3CN, rt, 375.79 MHz) spectrum of AgC_6F_4Br.

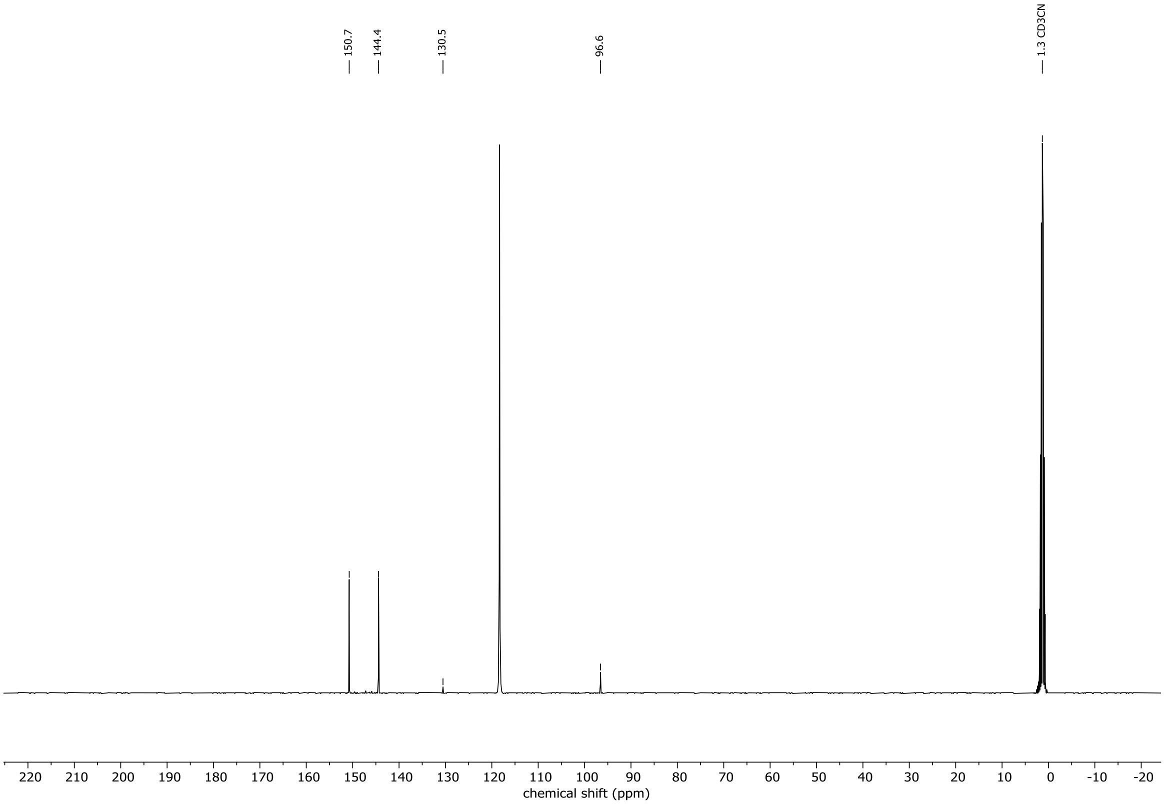

Figure S8. ^{13}C NMR (CD_3CN, rt, 100.42 MHz) spectrum of AgC_6F_4Br.

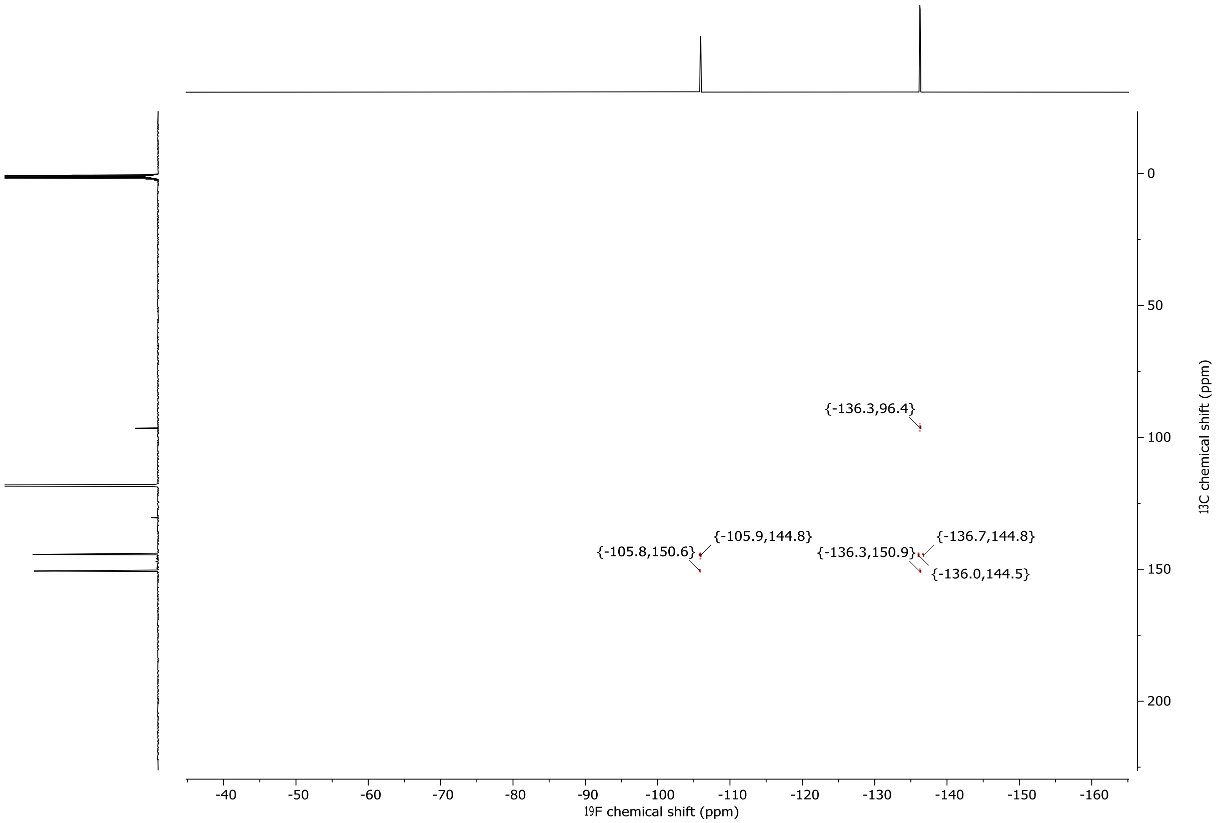

Figure S9. ^{13}C,^{19}F HMBC NMR (CD_3CN, rt, 100.42 MHz, 375.79 MHz) spectrum of AgC_6F_4Br.

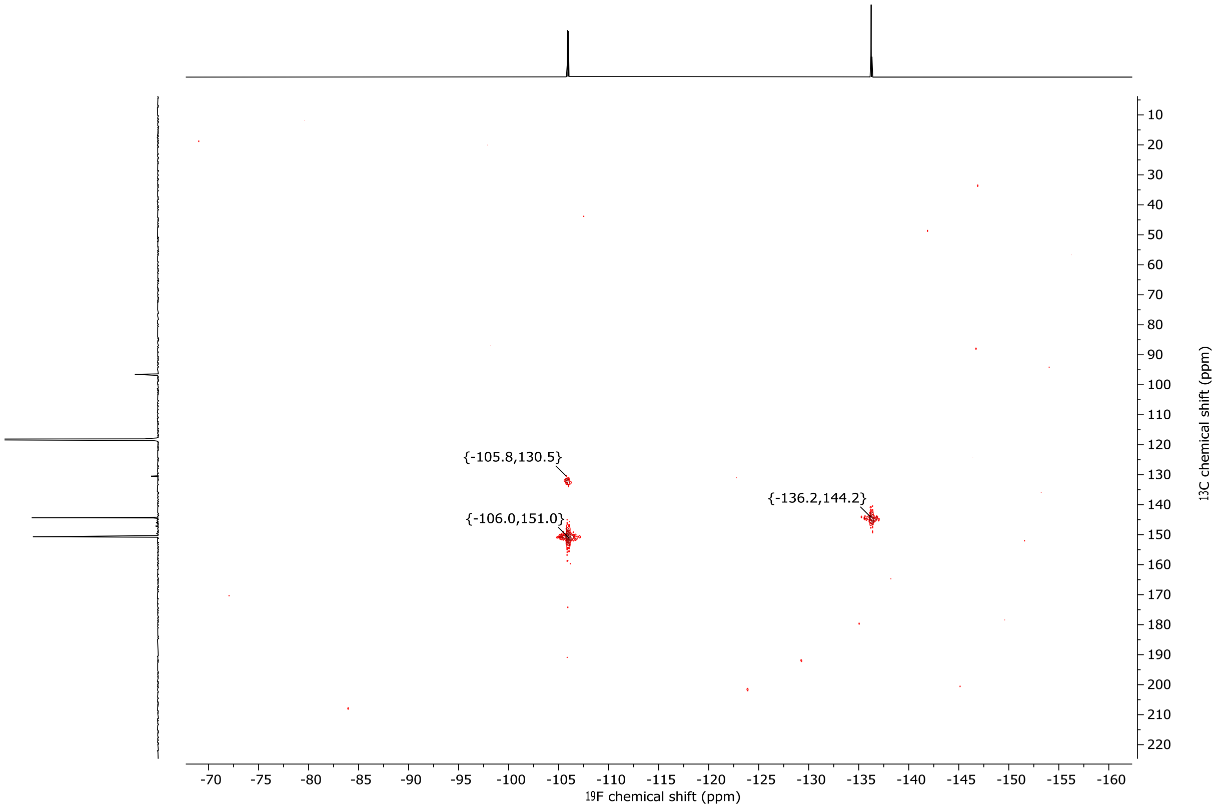

Figure S10. $^{13}C,^{19}F$ HMQC NMR (CD_3CN, rt, 100.42 MHz, 375.79 MHz) spectrum of AgC_6F_4Br.

Tris(4-bromo-2,3,5,6-tetrafluorophenyl)borane

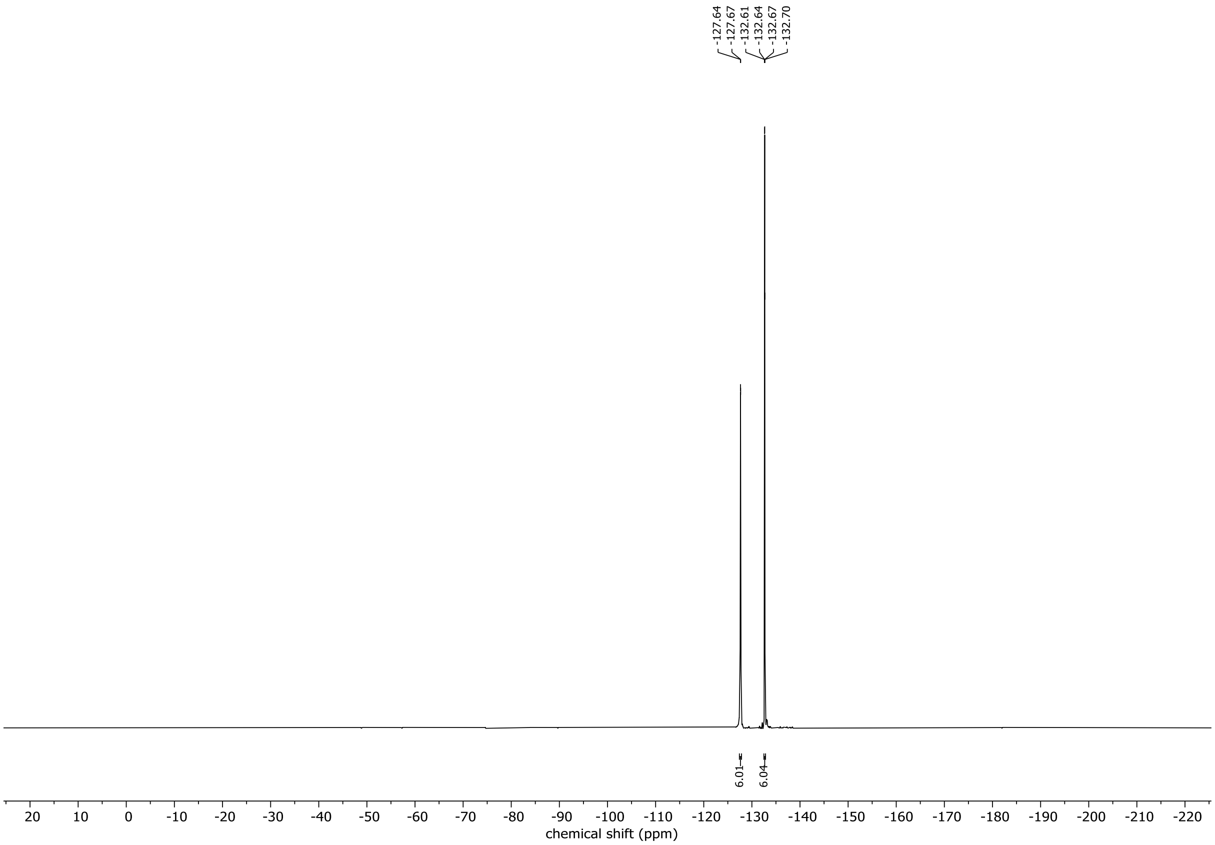

Figure S11. ^{19}F NMR (CD_2Cl_2, rt, 375.79 MHz) spectrum of $B(C_6F_4Br)_3$.

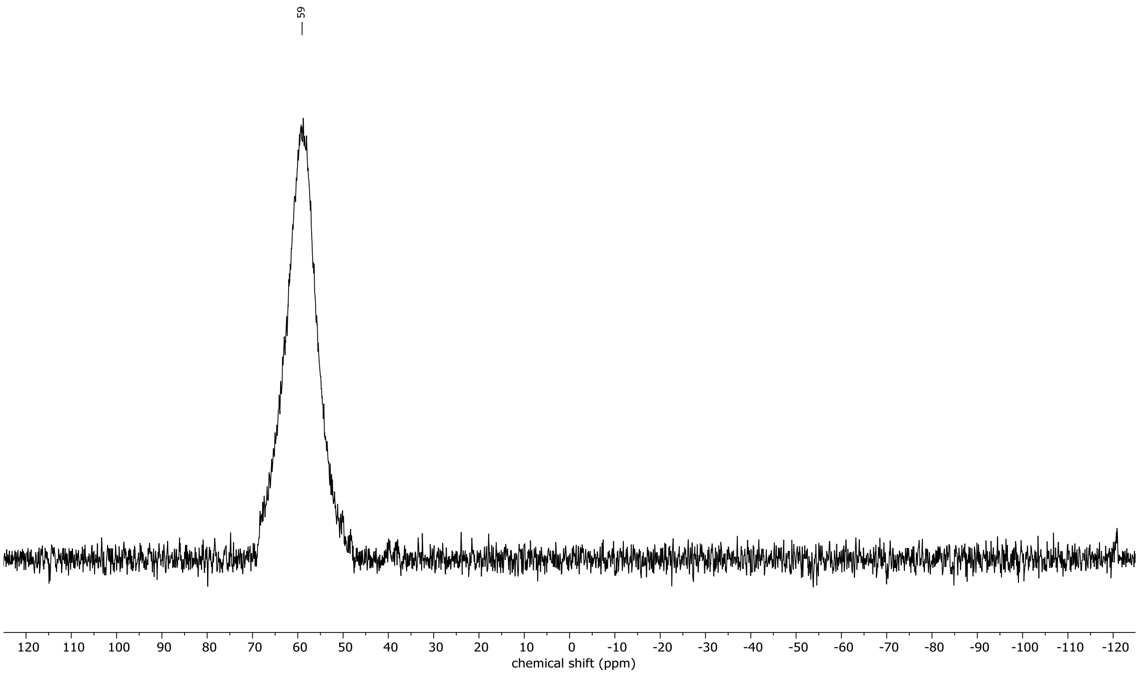

Figure S12. ^{11}B NMR (CD_2Cl_2, rt, 128.14 MHz) spectrum of $B(C_6F_4Br)_3$.

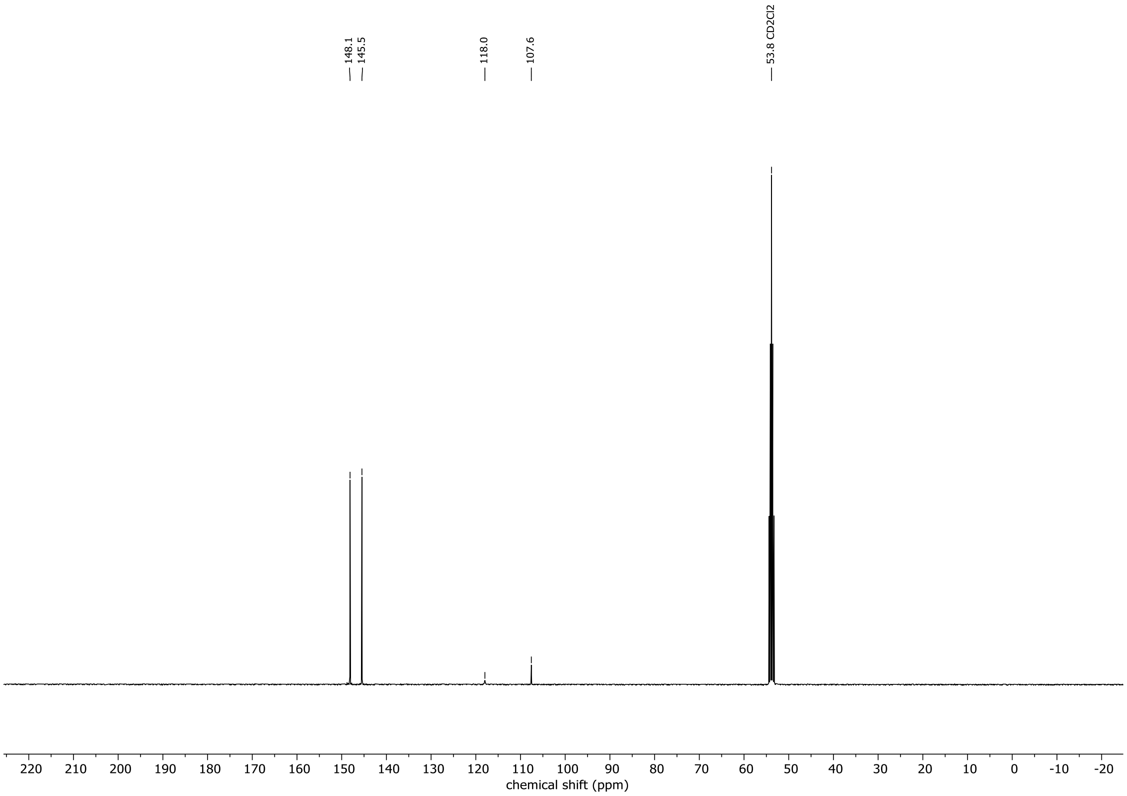

Figure S13. ^{13}C NMR (CD_2Cl_2, rt, 100.42 MHz) spectrum of $B(C_6F_4Br)_3$.

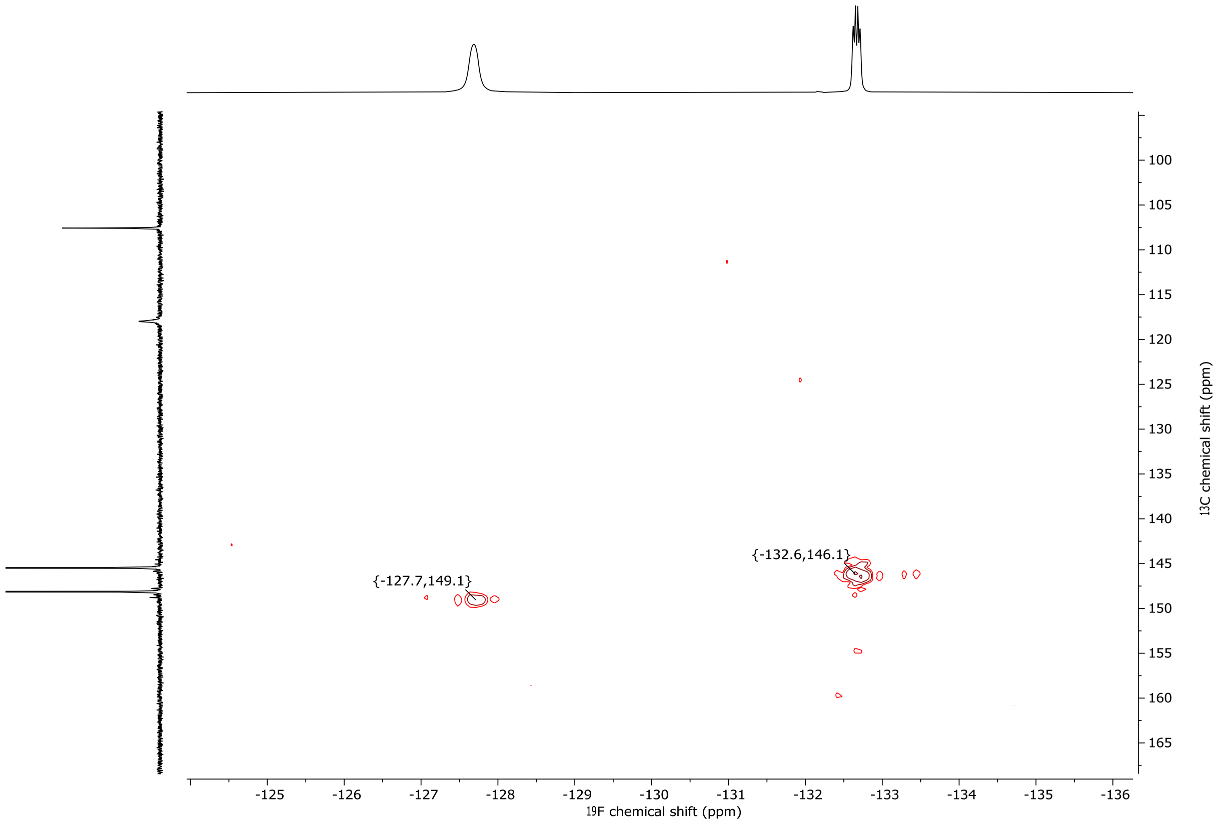

Figure S14. $^{13}C,^{19}F$ HMQC NMR (CD_2Cl_2, rt, 100.42 MHz, 375.79 MHz) spectrum of $B(C_6F_4Br)_3$.

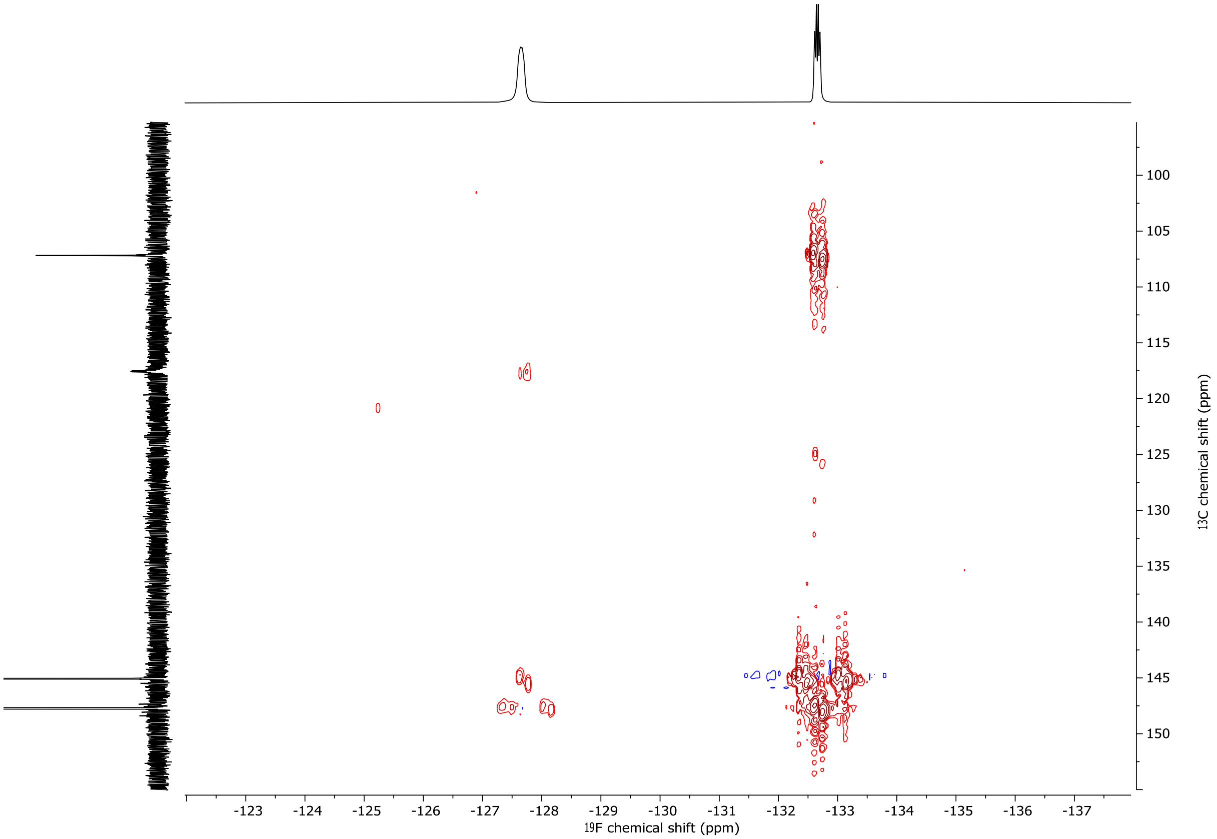

Figure S15. $^{13}C,^{19}F$ HMBC NMR (CD_2Cl_2, rt, 100.42 MHz, 375.79 MHz) spectrum of $B(C_6F_4Br)_3$.

Bis(4-bromo-2,3,5,6-tetrafluorophenyl)(hydroxy)borane

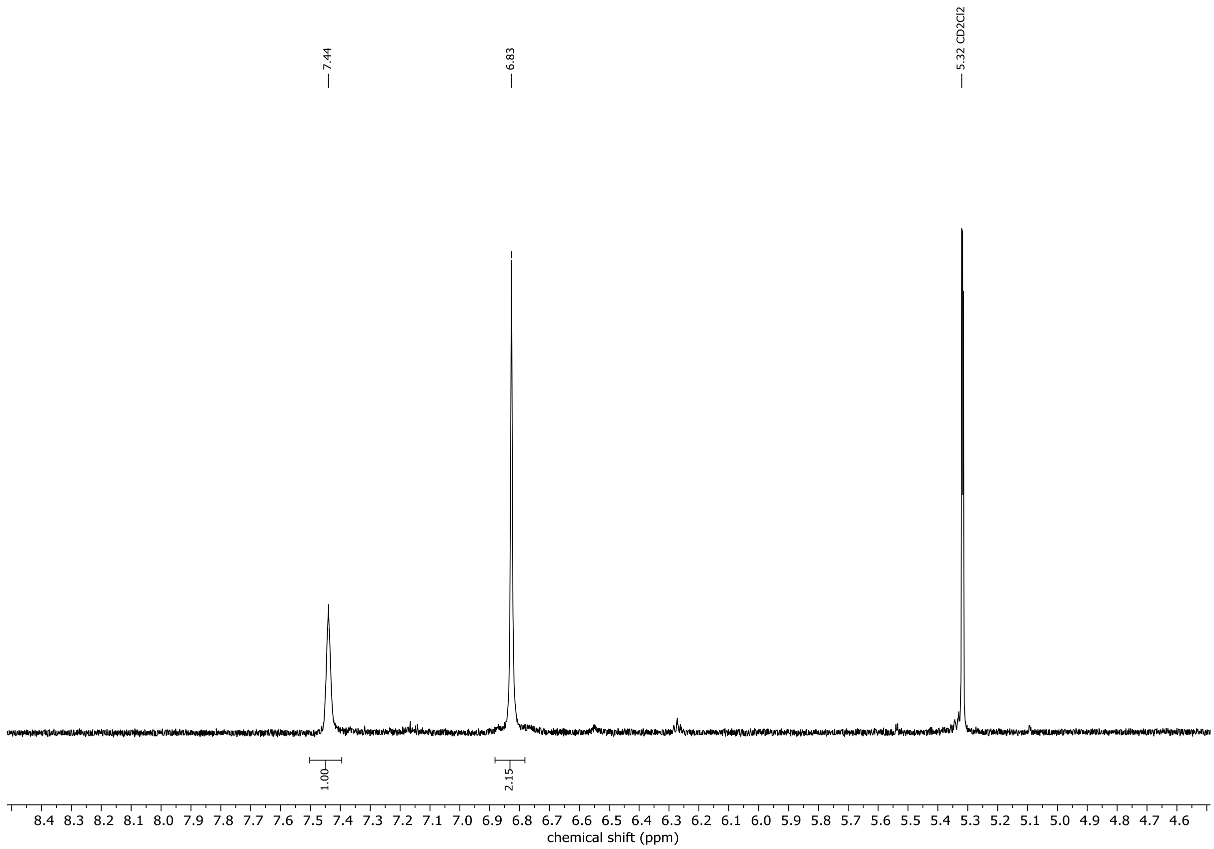

Figure S16. ^{1}H NMR (CD_2Cl_2, rt, 400 MHz) spectrum of a mixture of $B(C_6F_4Br)_3$,$(C_6F_4Br)_2BOH$ ($\cdot H_2O$), $(C_6F_4Br)_2BOB(C_6F_4Br)_2$.

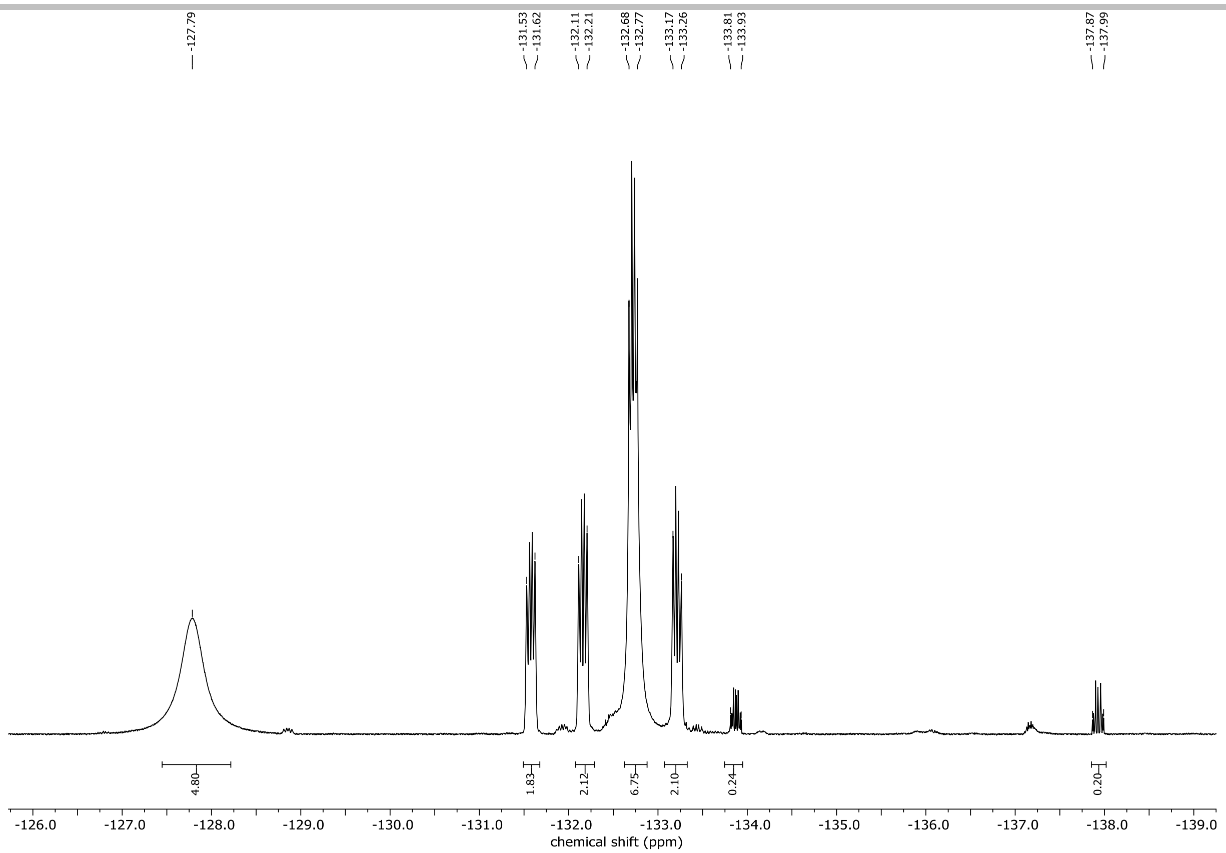

Figure S17. ^{19}F NMR (CD_2Cl_2, rt, 375.79 MHz) spectrum of a mixture of $B(C_6F_4Br)_3$, $(C_6F_4Br)_2BOH$, $(C_6F_4Br)_2BOB(C_6F_4Br)_2$ and C_6F_4BrH.

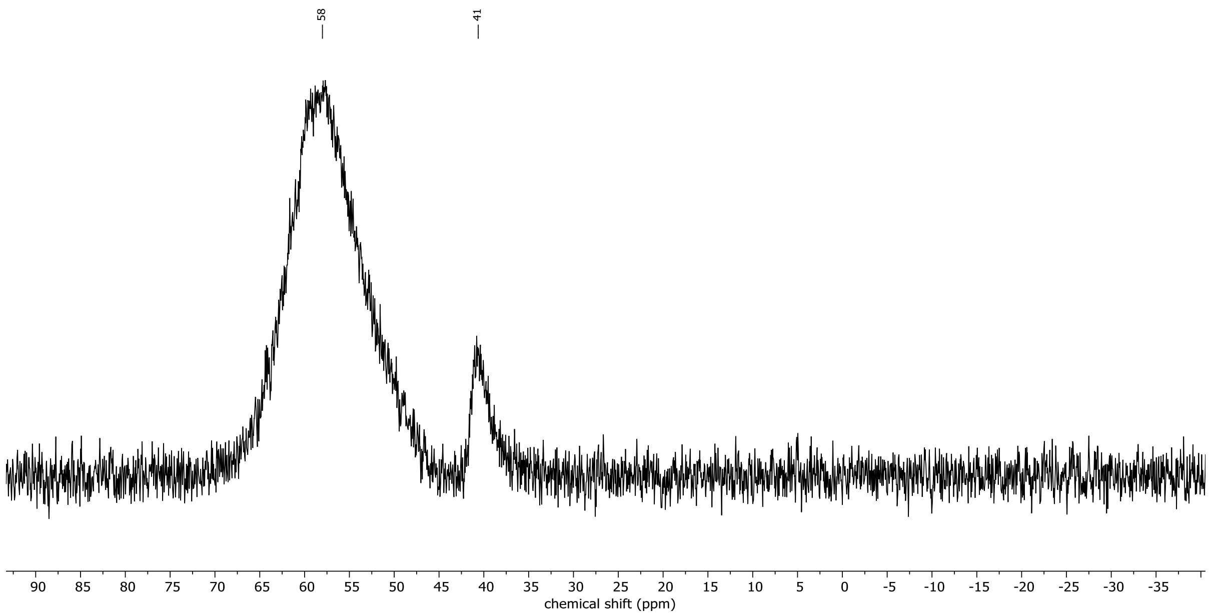

Figure S18. ^{11}B NMR (CD_2Cl_2, rt, 128.14 MHz) spectrum of a mixture of $B(C_6F_4Br)_3$ and $(C_6F_4Br)_2BOH$.

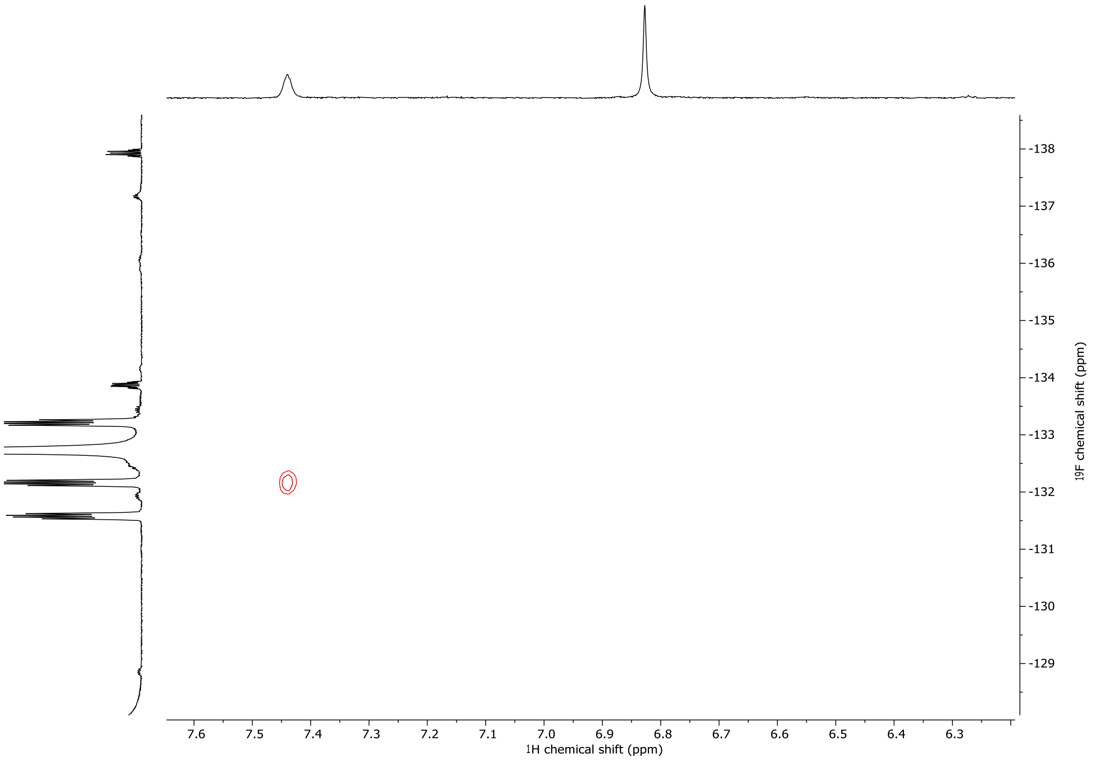

Figure S19. ^{1}H,^{19}F HETCOR NMR (CD_2Cl_2, rt, 400 MHz, 375.79 MHz) spectrum of a mixture of $(C_6F_4Br)_2BOH \cdot H_2O$, $B(C_6F_4Br)_3$ and $(C_6F_4Br)_2BOB(C_6F_4Br)_2$.

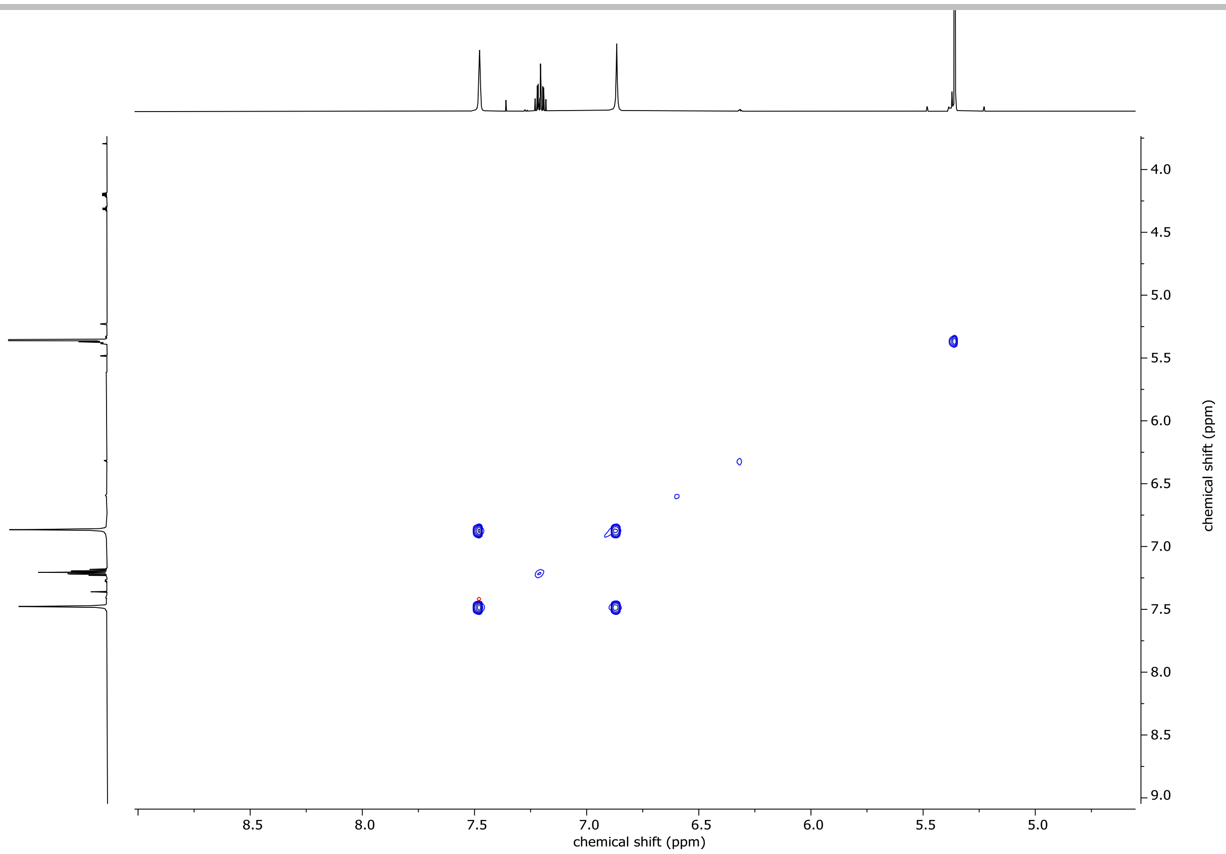

Figure S20. $^{1}H,^{1}H$ COSY NMR (CD_2Cl_2, rt, 700.23 MHz) spectrum of a mixture of $(C_6F_4Br)_2BOH \cdot H_2O$, $B(C_6F_4Br)_3$ and C_6F_4BrH.

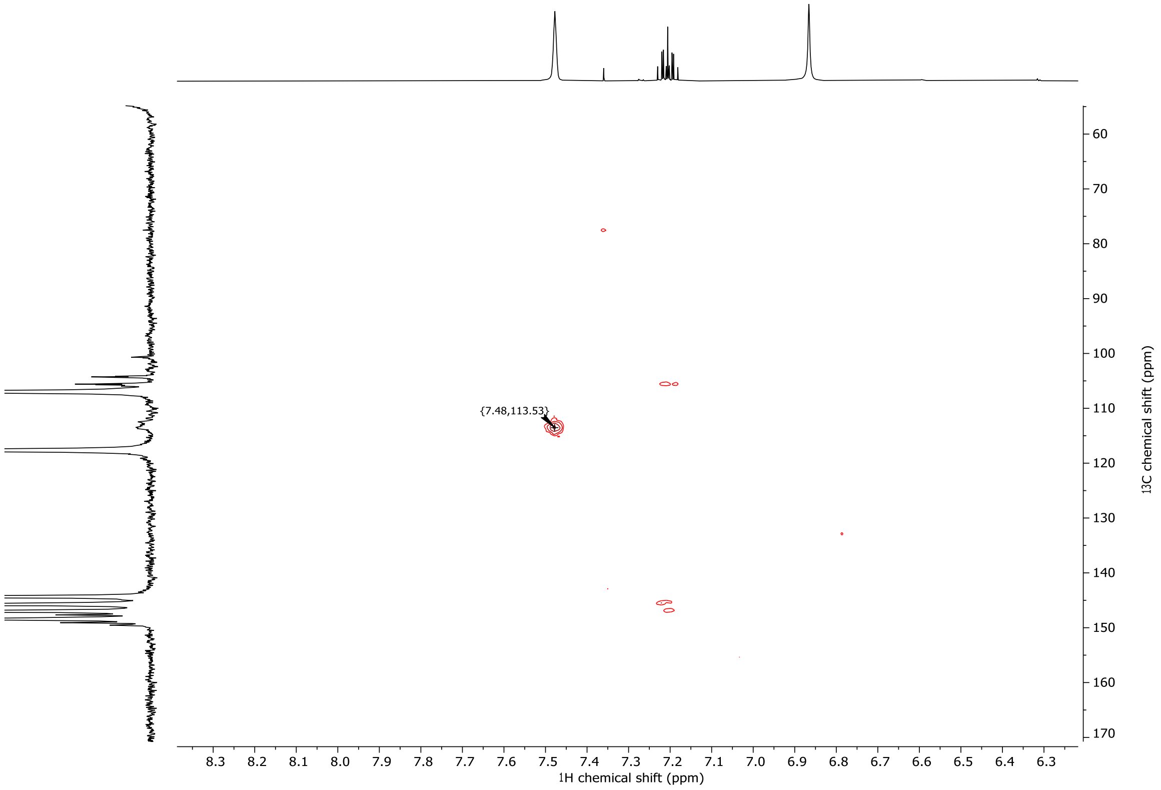

Figure S21. $^{1}H,^{13}C$ HMBC NMR (CD_2Cl_2, rt, 176 MHz, 700 MHz) spectrum of a mixture of $(C_6F_4Br)_2BOH \cdot H_2O$, $B(C_6F_4Br)_3$ and C_6F_4BrH.

Tris(4-bromo-2,3,5,6-tetrafluorophenyl)borane · triethylphosphine oxide

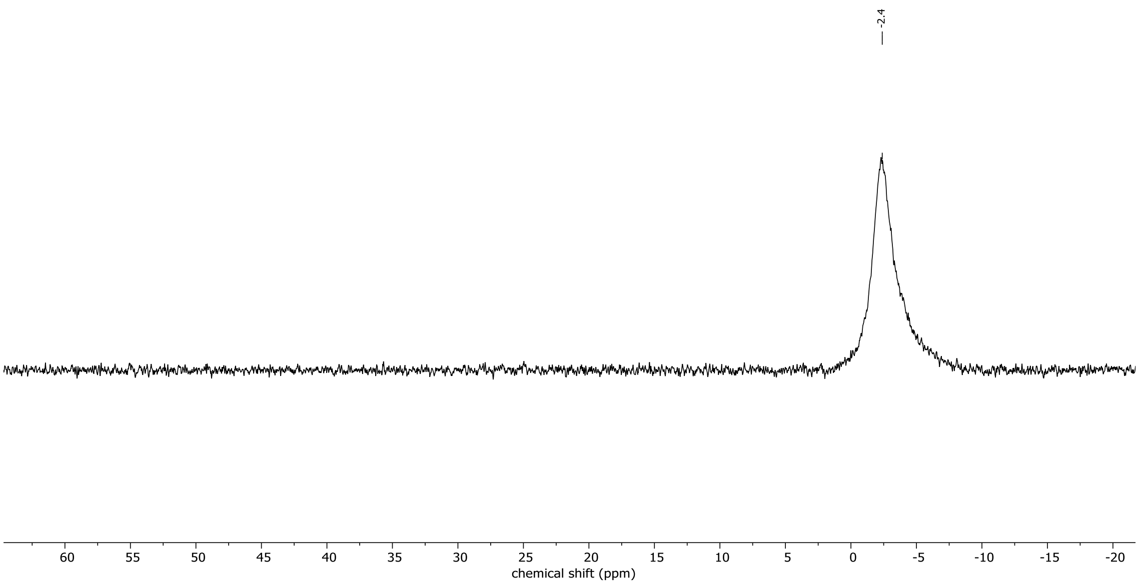

Figure S22. ^{11}B NMR (CD_2Cl_2, rt, 128.14 MHz) spectrum of the $B(C_6F_4Br)_3$•$POEt_3$ adduct.

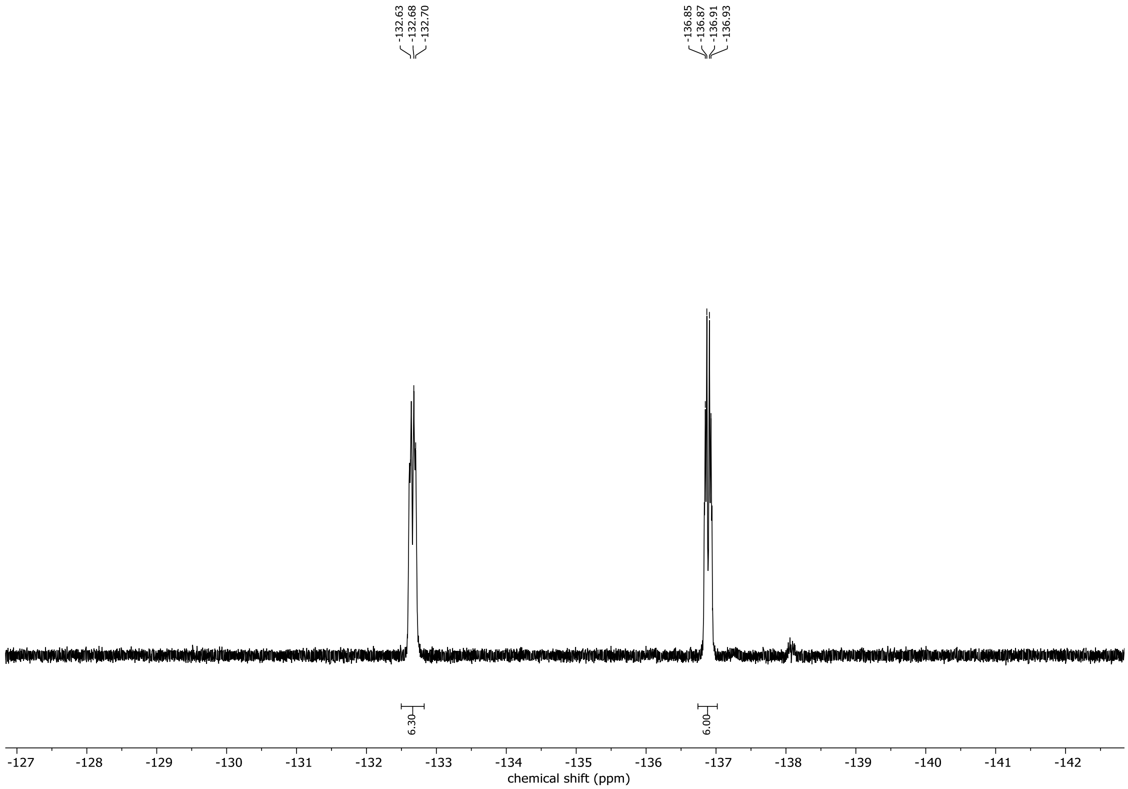

Figure S23. ^{19}F NMR (CD_2Cl_2, rt, 375.79 MHz) spectrum of the $B(C_6F_4Br)_3$•$POEt_3$ adduct.

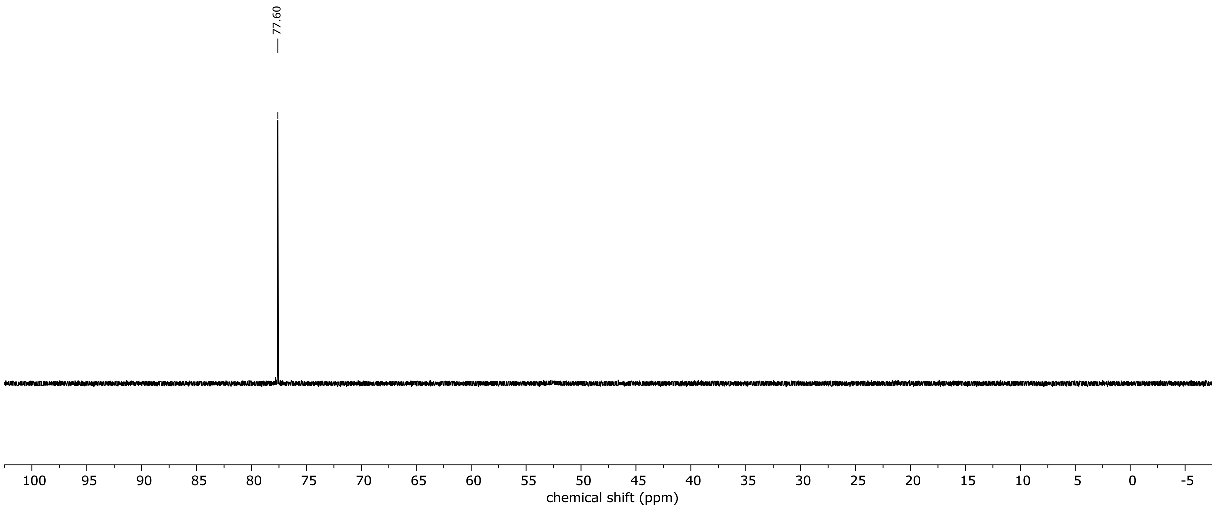

Figure S24. $^{31}P\{^1H\}$ NMR (CD_2Cl_2, rt, 161.7 MHz) spectrum of the $B(C_6F_4Br)_3$•$POEt_3$ adduct.

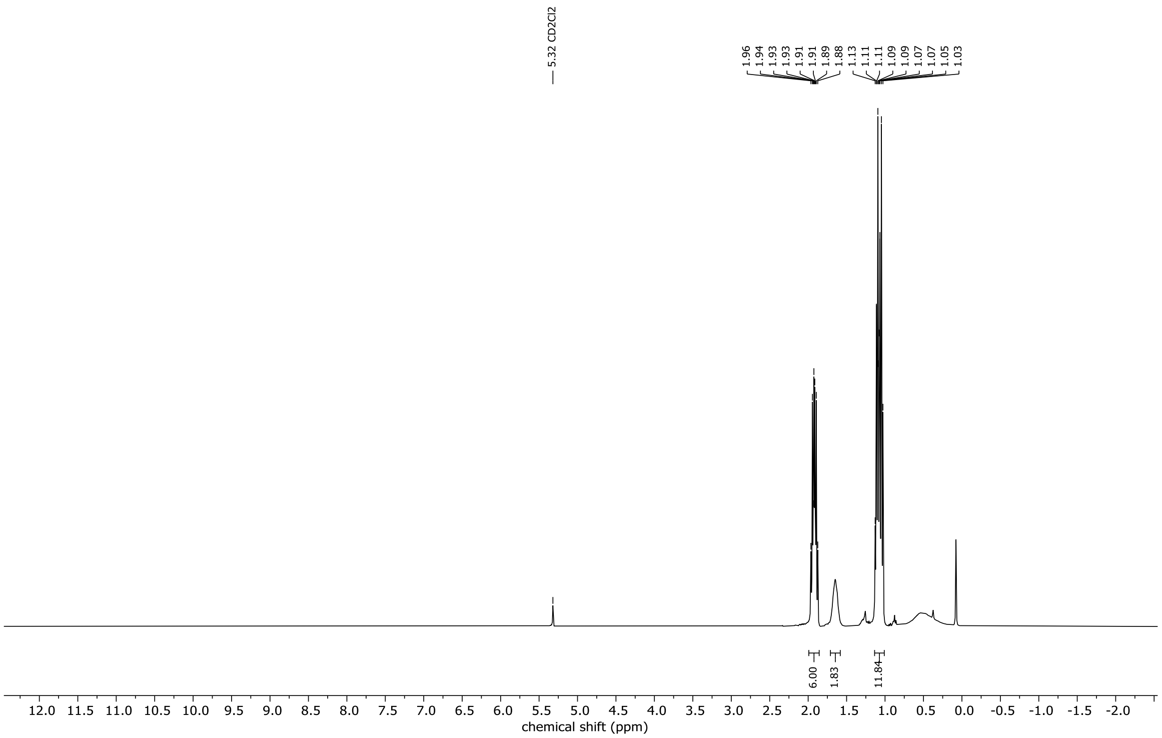

Figure S25. ^1H NMR (CD_2Cl_2, rt, 400 MHz) spectrum of the $B(C_6F_4Br)_3$•$POEt_3$ adduct.

IR Spectra

(4-Bromo-2,3,5,6-tetrafluorophenyl)silver

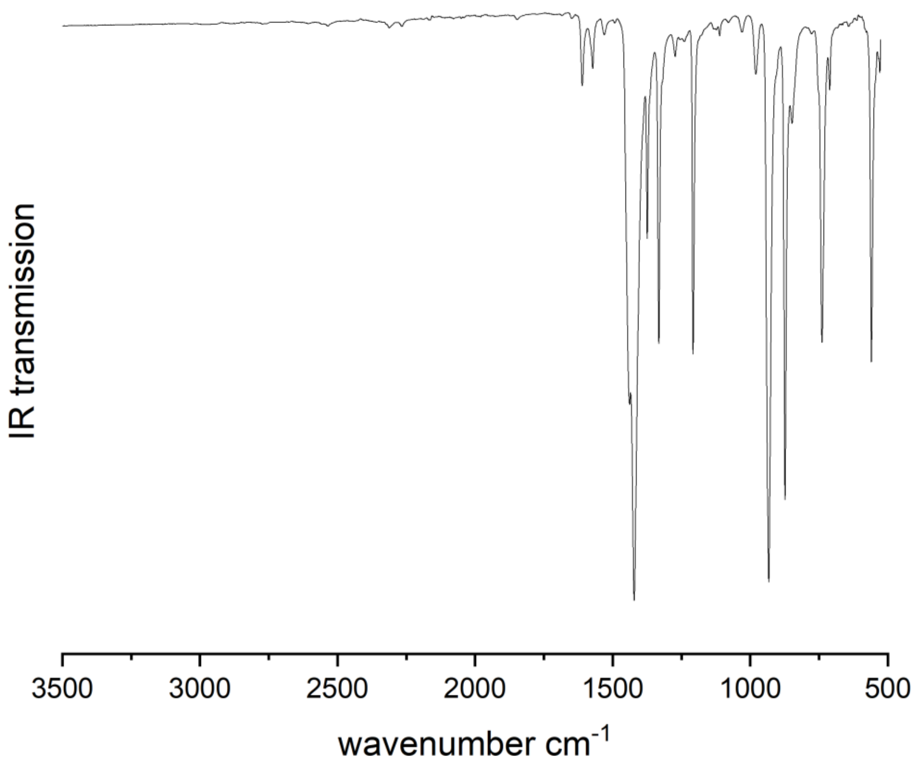

Figure S26. IR (ATR, rt) spectrum of AgC_6F_4Br.

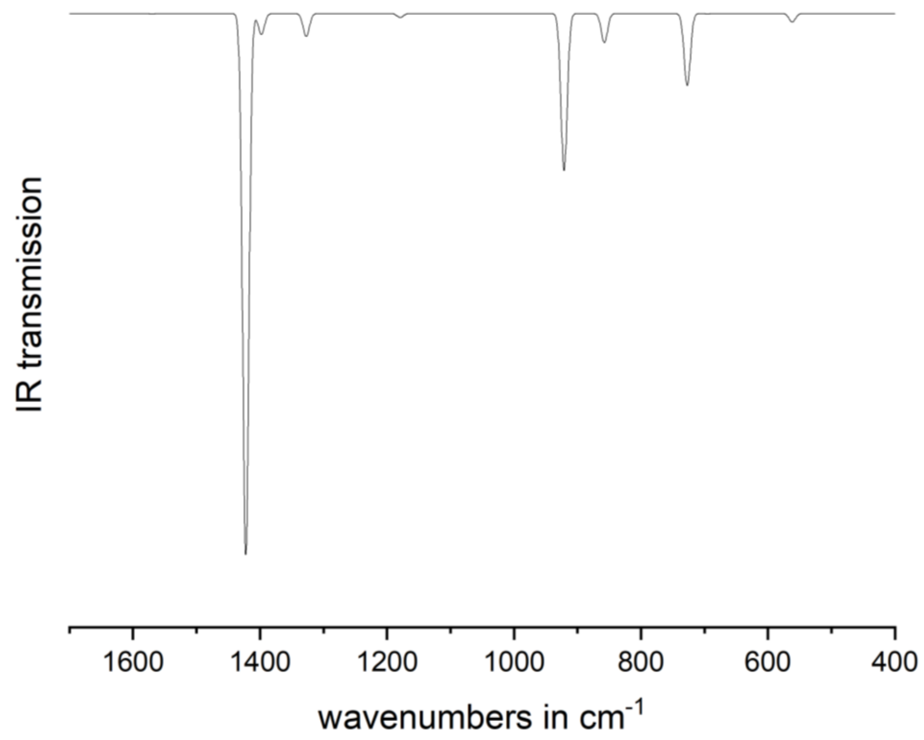

Figure S27. IR (calculated) spectrum of AgC_6F_4Br.

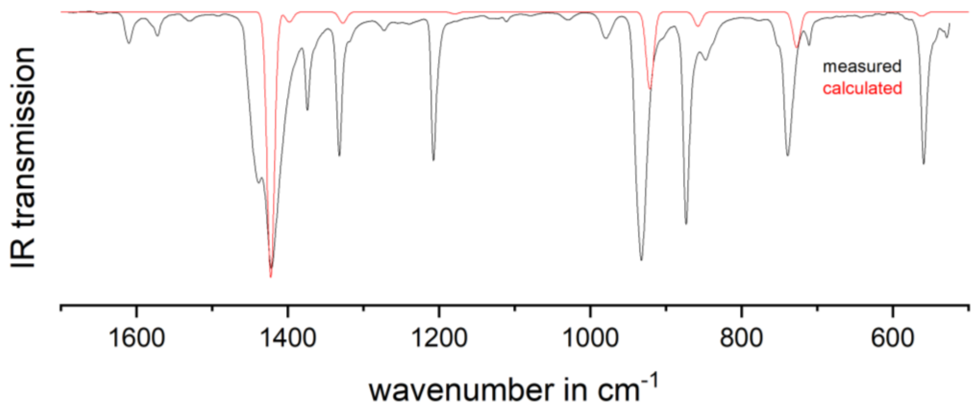

Figure S28. Stacked infrared spectra (calculated and measured) of AgC_6F_4Br.

Tris(4-bromo-2,3,5,6-tetrafluorophenyl)borane

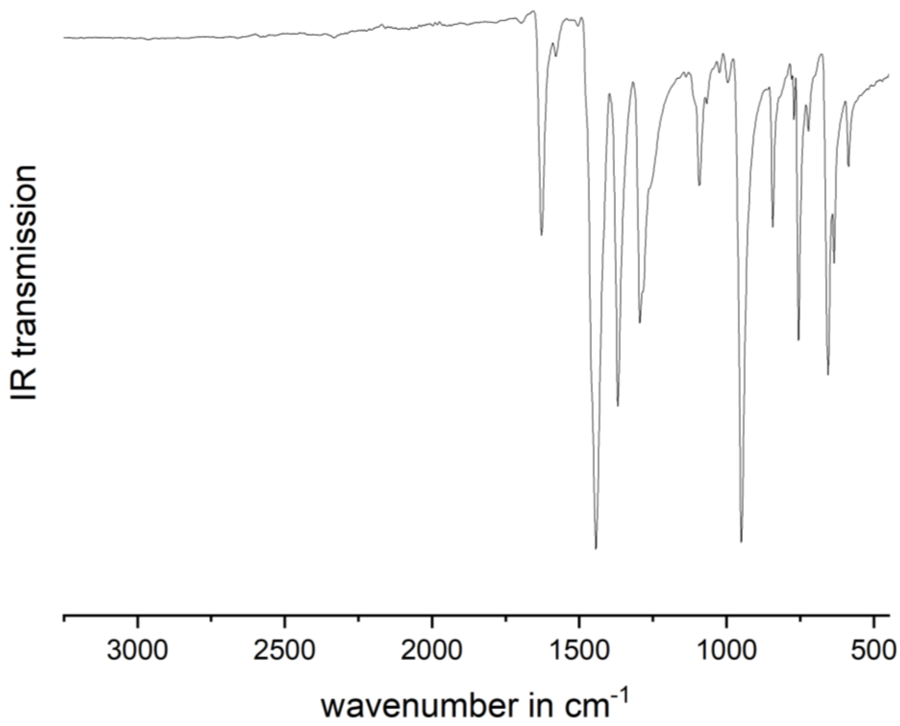

Figure S29. IR (ATR, rt) spectrum of $B(C_6F_4Br)_3$.

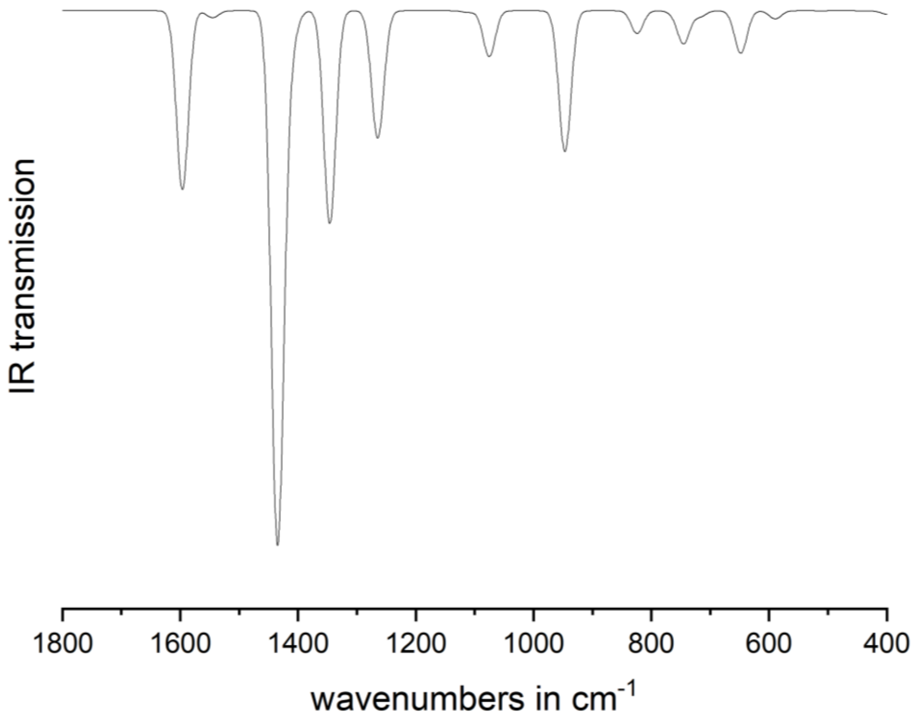

Figure S30. IR (calculated) spectrum of $B(C_6F_4Br)_3$.

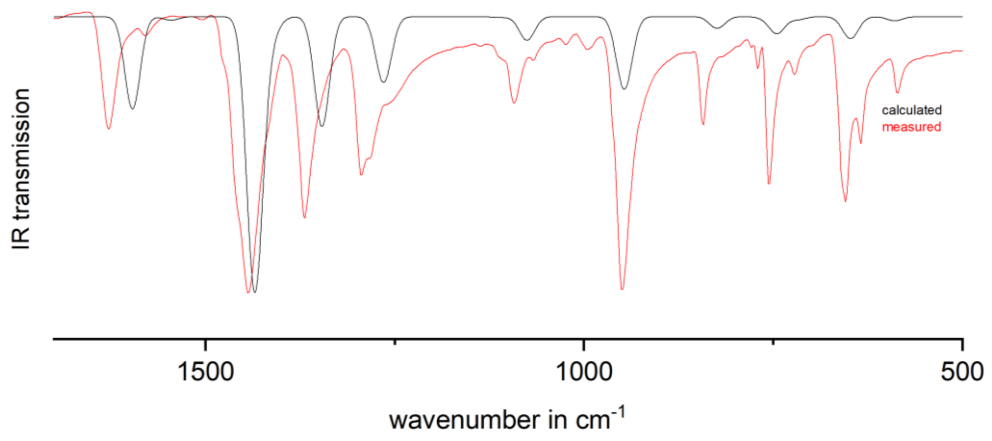

Figure S31. Stacked infrared spectra (calculated and measured) of $B(C_6F_4Br)_3$.

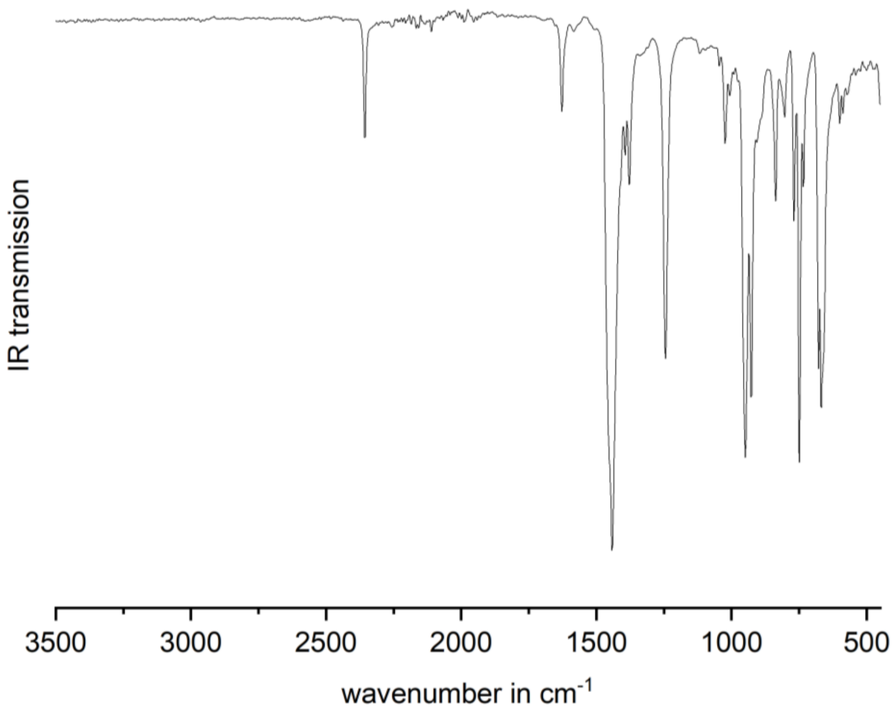

Figure S32. IR (ATR, rt) spectrum of $B(C_6F_4Br)_3$•CD_3CN.

Mass Spectra

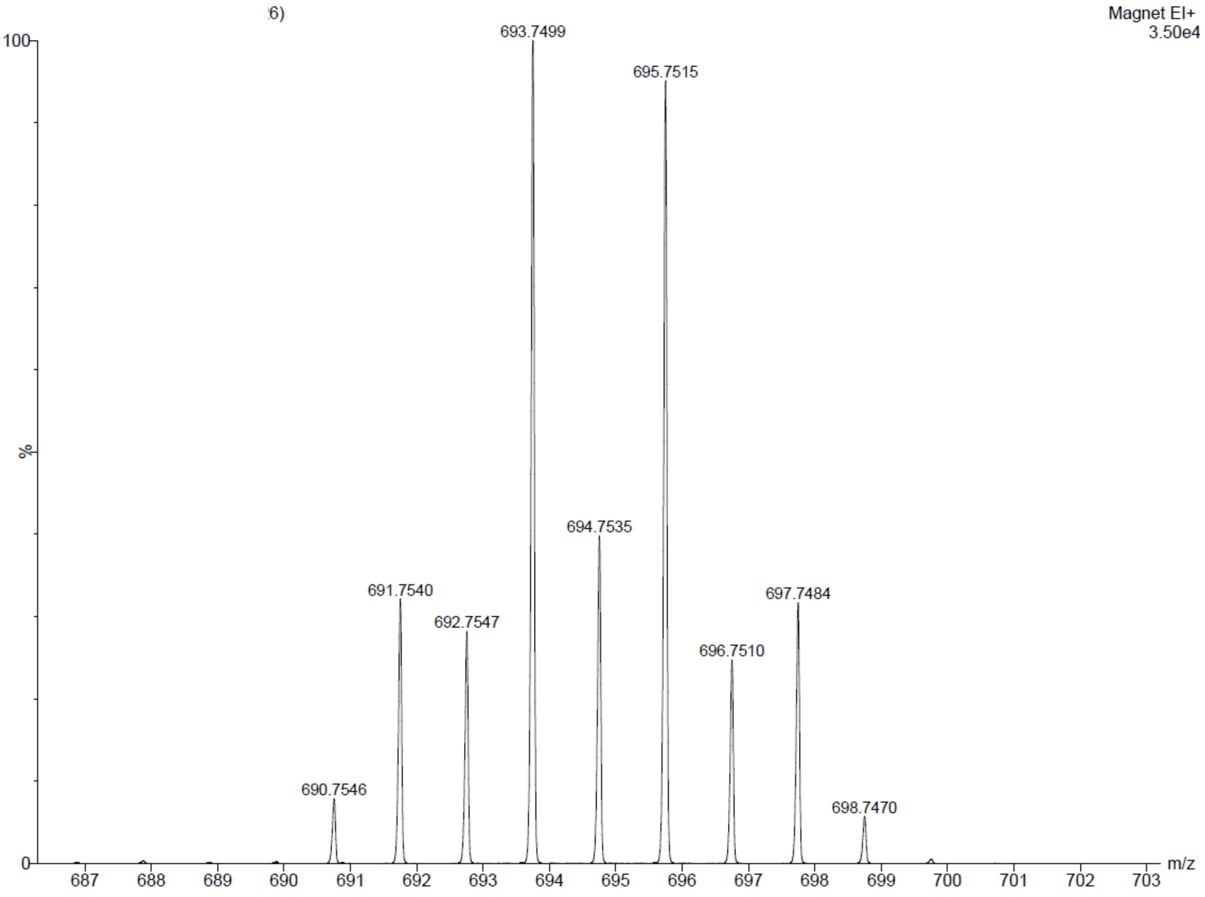

Figure S33. EI-MS (+) mass spectrum of $B(C_6F_4Br)_3$.

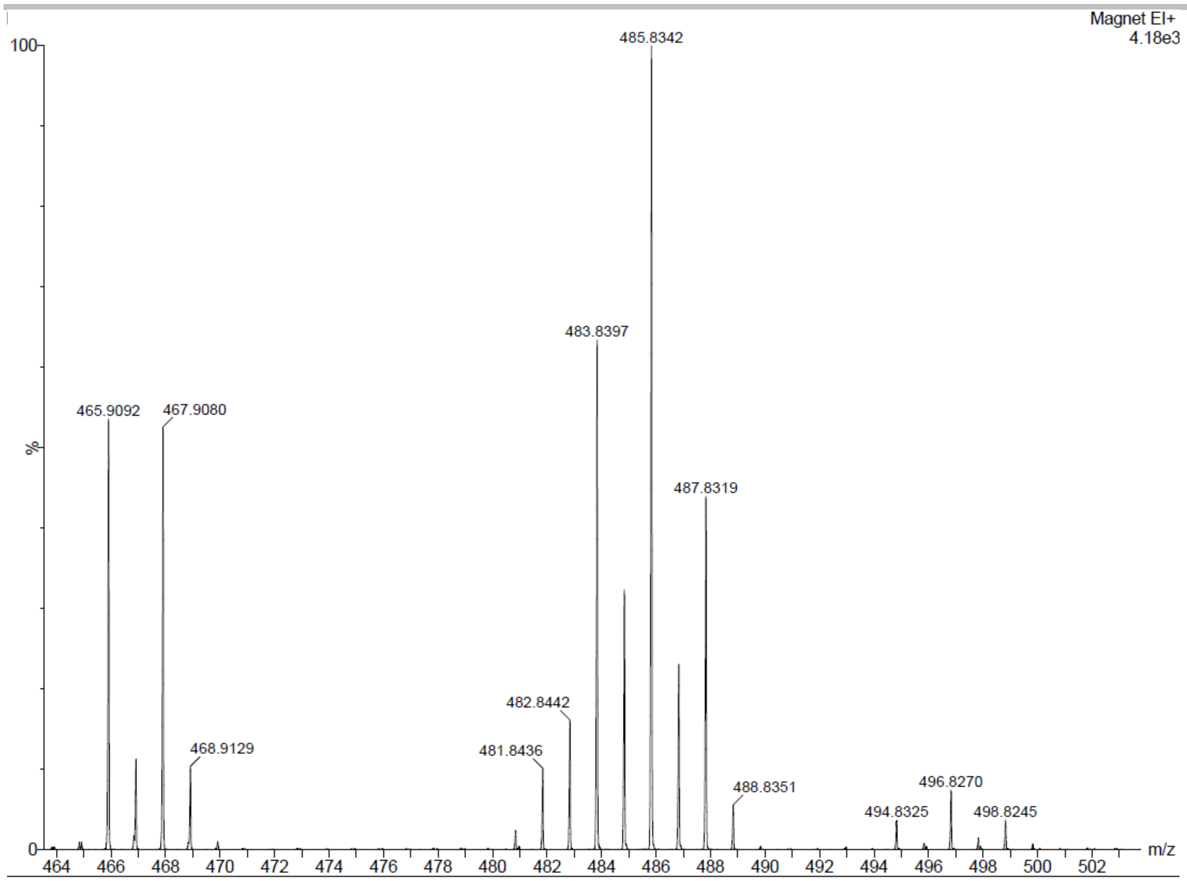

Figure S34. EI-MS (+) mass spectrum of $HOB(C_6F_4Br)_2$.

Quantum Chemical Calculations

Fluoride and hydride ion affinities (FIA and HIA) were calculated with Me_3SiF or Me_3SiH as a reference system as described in literature:[38]

$BR_3 + Me_3SiF \rightarrow BR_3F^- + Me_3Si^+$

$BR_3 + Me_3SiH \rightarrow BR_3H^- + Me_3Si^+$

Table S1. Calculated FIAs and HIAs in kJ/mol of several *para*-substituted perhalogenated phenylborane Lewis acids.

Lewis acid	FIA ($kJmol^{-1}$)	HIA ($kJmol^{-1}$)
$B(C_6F_4H)_3$	422	453
$B(C_6F_5)_3$	443.5	475
$B(C_6F_4Cl)_3$	447	480
$B(C_6F_4Br)_3$	448.5	482
$B(C_6F_4I)_3$	445	479
$B(C_6F_4CF_3)_3$	492.5	529

AgC_6F_4Br
neutral, diamagnetic, E = –3349.47477400 Hartree

Table S2. Structure optimized coordinates (x,y,z) for AgC_6F_4Br.

Ag1	0.0012190000	3.1789730000	0.0000000000
Br2	-0.0010430000	-3.6068370000	0.0000000000
F3	-2.3659020000	1.0141900000	0.0000000000
F4	-2.3660210000	-1.6627280000	0.0000000000
F5	2.3659240000	1.0129810000	0.0000000000
F6	2.3648510000	-1.6637230000	0.0000000000
C7	-1.1778890000	0.3733180000	0.0000000000
C8	0.0000000000	1.0916030000	0.0000000000
C9	1.1775390000	0.3727360000	0.0000000000
C10	-1.1969070000	-1.0140780000	0.0000000000
C11	1.1960770000	-1.0146520000	0.0000000000
C12	-0.0005660000	-1.7220750000	0.0000000000

Table S3. Calculated IR frequencies for AgC_6F_4Br.

Frequencies in cm^{-1}	Intensities
50.1411	0.5191
78.5966	0.678644
117.107	0.153055
118.589	3.77307e-05
154.118	0.012753
158.221	0.762199
193.981	0.0473144
250.184	0.439299
291.363	0.0211669
300.561	0.0548039
311.998	0.387287
396.525	0
414.743	0.0180542
431.981	0.0211858
491.752	0.0343161
561.768	1.57779
655.859	0
695.46	0.0701226
705.771	0.000792346
727.161	13.3108
857.613	5.35052
921.164	28.9913
1087.99	0.00281094
1179.22	0.720676
1268.49	0.0165072
1327.13	4.21066
1397.81	3.8166
1422.43	100
1559.26	0.00328257
1570.27	0.0779329

$B(C_6F_4Br)_3$

neutral, diamagnetic, sum of electronic and zero-point energies= -9632.070216 Hartree

Table S4. Structure optimized coordinates (x,y,z) for $B(C_6F_4Br)_3$.

Br1	-6.1907980000	1.0263320000	-0.0000160000
Br2	2.2067280000	-5.8735440000	0.0001350000
Br3	3.9848520000	4.8468650000	-0.0001490000
F4	-4.5705390000	-1.0860020000	1.5079920000
F5	-1.9568580000	-1.5293720000	1.5025950000
F6	-1.3587700000	2.0787810000	-1.5029570000
F7	-3.9755080000	2.5027320000	-1.5082170000
F8	3.2254810000	-3.4141150000	1.5082400000
F9	2.3024210000	-0.9289130000	1.5025660000
F10	-1.1209750000	-2.2155440000	-1.5032040000
F11	-0.1794300000	-4.6935720000	-1.5083340000
F12	-0.3453670000	2.4589450000	1.5037810000
F13	1.3460180000	4.5002130000	1.5089070000
F14	4.1544160000	2.1905170000	-1.5091120000
F15	2.4782860000	0.1367300000	-1.5035850000
C16	-4.3382000000	0.7193440000	0.0000980000
C17	-3.4877380000	1.5142760000	-0.7598850000
C18	-3.7897920000	-0.3075900000	0.7600080000
C19	-2.4237580000	-0.5294030000	0.7417130000
C20	-2.1232130000	1.2834650000	-0.7414330000
C21	1.5460460000	-4.1157410000	0.0002220000
C22	2.1610030000	-3.1272700000	0.7601900000
C23	0.4324550000	-3.7768290000	-0.7599040000
C24	-0.0501140000	-2.4797830000	-0.7414780000
C25	1.6699340000	-1.8334050000	0.7418200000
C26	0.5481240000	-1.4604040000	0.0003560000
C27	-1.5397050000	0.2555030000	0.0002900000
C28	2.1722200000	1.1964910000	-0.7419560000
C29	0.9903910000	1.2054170000	0.0000560000
C30	0.7534950000	2.3632640000	0.7420920000
C31	1.6289960000	3.4350490000	0.7603620000
C32	3.0547380000	2.2625000000	-0.7604530000
C33	2.7921890000	3.3963730000	-0.0000770000
B34	-0.0004730000	0.0002040000	0.0001780000

Table S5. Calculated IR frequencies for $B(C_6F_4Br)_3$.

Frequencies in cm^{-1}	Intensities
15.5693	0.0648588
15.7509	0.0655146
20.9452	0.0175053
21.0731	0.0181611
21.545	0.0017993
32.5844	1.68159e-05
79.2776	0.0041367
79.3803	0.00422078
81.9273	0.0199772

120.473	0
121.628	0.00168159
121.68	0.00221969
121.853	0.00255601
123.547	0.150048
123.599	0.150435
127.166	3.36317e-05
137.897	0.015437
137.966	0.0162777
156.323	0.0718878
162.159	0.050868
162.209	0.0501953
211.332	0.575052
211.426	0.534122
211.464	0.54517
245.974	0.0106108
245.995	0.0106781
253.631	0
301.988	0.0301004
302.038	0.0293269
303.182	5.04476e-05
306.697	0.112481
307.712	0.0246689
307.739	0.0235758
330.241	0.192945
355.062	2.31906
355.09	2.31549
394.048	0.503854
394.127	0.504038
395.197	0.00142935
401.722	0.510883
401.748	0.509924
431.258	0.0723586
432.868	0.0129146
432.903	0.0128978
434.702	0
488.899	0.00183293
488.94	0.00176566
494.301	0
512.976	0.0150838
589.553	2.03099
589.588	2.03433
640.549	1.77493
648.405	9.95055
648.464	9.94972
661.348	0.27652
661.383	0.279328
663.554	5.04476e-05
717.704	1.46932
717.725	1.47092
720.029	0.0185143

745.111	8.16364
745.145	8.17165
761.588	1.22537
772.676	0
824.125	5.73913
824.174	5.73569
924.693	1.68159e-05
944.033	28.8672
948.487	22.0662
948.498	22.0483
1075.25	11.5261
1075.27	11.5559
1107.77	0.312876
1111.62	0.286088
1111.64	0.287669
1235.03	1.68159e-05
1258.75	0.198578
1260.17	8.45111
1260.18	8.29627
1266.04	24.8454
1266.07	24.8312
1346.01	53.871
1346.03	53.7796
1360.23	5.04476e-05
1410.09	6.2855
1410.1	6.21813
1410.35	0.00418715
1428.74	90.0063
1437	99.9065
1437.04	100
1545.11	3.39537
1548.14	0.048581
1548.17	0.0471685
1596.27	45.2656
1596.27	45.2359
1597.52	0.000773529

$(C_6F_4Br)_2BOH$

neutral, diamagnetic, E = –6505.68772451 Hartree

Table S6. Structure optimized coordinates (x,y,z) for $(BrF_4C_6)_2BOH$.

Br	-5.5596330000	-1.1432470000	0.0738640000
Br	5.5186010000	-1.2780610000	-0.2599380000
F	-3.0649660000	-1.7693170000	1.7372550000
F	-0.6906130000	-0.5493600000	1.7492830000
F	-2.2236270000	2.4781340000	-1.5327220000
F	-4.5926830000	1.2610620000	-1.5552870000
F	2.5854670000	2.7714560000	1.0574450000
F	4.8942940000	1.4846550000	0.9072520000
F	2.7419090000	-2.1545090000	-1.2003770000
F	0.4311220000	-0.8886540000	-1.0498090000
O	-0.0452190000	3.0920910000	0.3301820000
C	-3.8875630000	-0.2816210000	0.0913910000
C	-2.8707160000	-0.7270790000	0.9283750000
C	-3.6452100000	0.8111410000	-0.7318850000
C	-2.4106510000	1.4425660000	-0.7037570000
C	-1.6449030000	-0.0841960000	0.9237370000
C	-1.3746220000	1.0141470000	0.1178380000
C	1.5043770000	-0.2743310000	-0.5392050000
C	1.3883010000	0.9916650000	0.0364790000
C	2.5837230000	1.5414490000	0.4994550000
C	3.8020690000	0.8902010000	0.4294760000
C	2.7112860000	-0.9503070000	-0.6329870000
C	3.8765900000	-0.3743260000	-0.1428000000
B	0.0134980000	1.7554270000	0.1610870000
H	0.8061630000	3.5348620000	0.3916270000

Table S7. Calculated IR frequencies for $(C_6F_4Br)_2BOH$.

Frequencies in cm^{-1}	Intensities
13.6126	0.0343554
21.4918	0.00141754
26.9906	0.0046508
55.0854	0.104691
78.8455	0.021948
105.552	0.124552
119.016	0.058581
122.68	0.0354385
123.667	0.00450746
128.412	0.0103687
135.949	0.00415705
156.588	0.0269173
162.655	0.0442145
201.409	0.488557
204.183	0.146166
231.222	0.153174
248.036	0.0194474
269.957	0.477711
295.157	0.0914392

302.876	0.0895757
307.538	0.155165
312.903	0.423669
317.847	0.137533
326.933	1.09358
387.161	0.437239
393.831	0.0531816
395.402	0.0137931
409.261	1.61826
432.111	0.0203393
433.895	0.058135
458.158	0.0303895
489.454	0.0412042
497.957	0.056383
517.12	0.220658
585.719	2.53864
619.502	5.29193
654.795	12.1544
659.636	0.438258
663.059	6.19546
665.527	7.91328
721.294	0.392244
728.361	4.59287
737.34	0.384567
754.457	2.0935
772.587	4.52149
808.716	10.286
918.292	2.10256
943.807	26.1342
947.799	27.3008
986.909	13.4023
1102.94	1.1802
1108.45	0.205065
1142.98	36.0413
1217.82	0.0322211
1259.01	7.57216
1262.36	1.9943
1267.54	50.8375
1330.64	18.049
1349.88	18.7893
1363.72	10.8644
1411.74	2.67955
1414.56	4.97211
1435.37	91.3083
1445.29	100
1550.22	0.701252
1555.87	0.560581
1597.6	22.4228
1602.07	19.387
3704.31	18.9603

$(C_6F_4Br)_2BOH \cdot H_2O$
neutral, diamagnetic, E = –6582.17053706 Hartree

Table S8. Structure optimized coordinates (x,y,z) for $(C_6F_4Br)_2BOH \cdot H_2O$.

Br	5.5853110000	-1.2964150000	-0.2063660000
Br	-5.2805710000	-1.6724800000	0.2152660000
F	2.9520630000	-1.9848660000	-1.6112350000
F	0.6126040000	-0.7260330000	-1.5247640000
F	2.4587430000	2.4922280000	1.4110250000
F	4.7924830000	1.2336270000	1.3212230000
F	-2.2947200000	2.1017400000	-1.7031870000
F	-4.5407170000	0.6847820000	-1.6002650000
F	-2.7269090000	-1.9468880000	1.8843180000
F	-0.4769210000	-0.5268180000	1.7808020000
O	-2.3890890000	4.2708970000	0.1082710000
O	0.0508100000	3.0180130000	-0.0019290000
C	3.9299640000	-0.4047760000	-0.1473320000
C	2.8407400000	-0.8858530000	-0.8632310000
C	3.7721920000	0.7476800000	0.6125120000
C	2.5495590000	1.4017680000	0.6423300000
C	1.6283400000	-0.2190250000	-0.8041900000
C	1.4382710000	0.9409320000	-0.0599600000
C	-1.4666100000	-0.2087160000	0.9314270000
C	-1.2985380000	0.8465810000	0.0429940000
C	-2.3762000000	1.1069480000	-0.7918430000
C	-3.5508700000	0.3752770000	-0.7601170000
C	-2.6318910000	-0.9564620000	0.9977800000
C	-3.6908680000	-0.6699140000	0.1446920000
B	0.0482720000	1.6815240000	-0.0075940000
H	-2.4244260000	5.2190250000	0.2615900000
H	-2.8121750000	4.1147510000	-0.7420660000
H	-0.8185780000	3.4620580000	0.0646240000

Table S9. Calculated IR frequencies for $(C_6F_4Br)_2BOH \cdot H_2O$.

Frequencies in cm^{-1}	Intensities
14.4415	0.0129586
19.5135	0.0253526
20.1241	0.0150818
35.1081	0.190393
62.3604	0.452319
74.4326	0.0727971
87.0217	0.129046
106.89	0.628026
118.42	0.135214
122.101	0.0909374
122.279	0.00723758
130.857	0.00465935
142.675	0.826003
147.093	0.212249
155.195	1.03214
160.053	0.379092

165.512	5.38659
203.484	0.146336
207.783	0.0602514
236.233	0.48812
245.989	0.131473
262.934	4.38113
295.525	0.60457
299.103	0.296623
302.033	3.55926
305.183	0.682616
309.252	1.959
312.657	2.1905
330.495	3.42217
352.907	1.24334
391.807	0.12619
393.691	0.024906
396.843	0.159218
417.368	0.261252
432.141	0.0200023
433.494	0.0289166
458.595	0.0966583
490.421	0.00263721
498.387	0.0827477
522.582	0.14899
604.487	0.33233
623.694	2.69033
659.231	0.0922265
661.154	0.0385302
671.055	8.50544
713.348	0.0743305
731.199	3.36647
737.745	0.187857
753.908	0.523928
770.893	2.5275
815.782	3.15335
897.047	5.10843
917.521	0.839896
941.243	13.2681
947.518	14.8149
1032.54	10.5888
1101.05	0.0168259
1107.47	0.108361
1196.26	12.2908
1219.2	1.4965
1259.18	5.45646
1262.59	0.631784
1269.21	24.6719
1354.27	11.2987
1357.97	1.33936
1367.28	7.32766
1412.64	1.49805

1413.19	1.4321
1435.54	56.1207
1442.77	49.6149
1553.22	0.660448
1554.13	0.113181
1566.57	7.95054
1597.97	10.2858
1600.9	7.60923
3369.06	100
3670.8	1.01431
3769.05	9.03679

Me_3Si^+
cationic, diamagnetic, sum of electronic and zero-point energies= -408.973937 Hartree

Table S10. Structure optimized coordinates (x,y,z) for Me_3Si^+.

Si	0.000000000	0.000000000	0.000005000
C	-0.641130000	1.710586000	-0.000002000
C	-1.160846000	-1.410528000	-0.000002000
C	1.801977000	-0.300058000	0.000001000
H	-0.254621000	2.244659000	0.874714000
H	-0.254569000	2.244674000	-0.874688000
H	-1.728346000	1.758452000	-0.000033000
H	-0.658695000	-2.376019000	-0.000001000
H	-1.816644000	-1.342817000	0.874698000
H	-1.816640000	-1.342815000	-0.874704000
H	2.071234000	-0.901950000	0.874631000
H	2.071238000	-0.901745000	-0.874771000
H	2.387038000	0.617566000	0.000101000

Me_3SiF
neutral, diamagnetic, sum of electronic and zero-point energies= -509.220634 Hartree

Table S11. Structure optimized coordinates (x,y,z) for Me_3SiF.

Si	0.000059000	-0.000140000	0.028750000
F	0.000174000	-0.000590000	1.648484000
C	-1.411074000	-1.085660000	-0.525033000
C	1.646063000	-0.678518000	-0.525167000
C	-0.235187000	1.764820000	-0.524377000
H	-2.365941000	-0.708237000	-0.155496000
H	-1.466527000	-1.124281000	-1.615031000
H	-1.289337000	-2.106963000	-0.160581000
H	2.468955000	-0.061132000	-0.161254000
H	1.798164000	-1.693782000	-0.155263000
H	1.706931000	-0.707506000	-1.615176000
H	0.568490000	2.403604000	-0.154502000
H	-0.241018000	1.832705000	-1.614337000
H	-1.180927000	2.169004000	-0.159750000

Me_3SiH
neutral, diamagnetic, sum of electronic and zero-point energies= -409.850856 Hartree

Table S12. Structure optimized coordinates (x,y,z) for Me_3SiH.

Si	0.000007000	-0.000036000	0.379148000
C	-1.497760000	-0.959742000	-0.222982000
C	1.580054000	-0.817199000	-0.222983000
C	-0.082302000	1.776956000	-0.222994000
H	-0.000025000	0.000111000	1.867612000
H	-2.425948000	-0.507463000	0.130361000
H	-1.532235000	-0.981514000	-1.314319000
H	-1.474600000	-1.992149000	0.129930000
H	2.462482000	-0.281027000	0.130264000
H	1.652452000	-1.847289000	0.130030000
H	1.616322000	-0.835798000	-1.314316000

H	0.772483000	2.355171000	0.131924000
H	-0.082093000	1.817933000	-1.314318000
H	-0.988889000	2.272436000	0.128515000

$(C_6F_4Br)_3BF^–$

anionic, diamagnetic, sum of electronic and zero-point energies= -9732.124657 Hartree

Table S13. Structure optimized coordinates (x,y,z) for $(C_6F_4Br)_3BF^–$

Br	2.929749000	-5.462500000	-0.568127000
Br	3.267589000	5.267295000	-0.568380000
Br	-6.196150000	0.195145000	-0.568251000
F	2.487982000	-1.016338000	2.039370000
F	3.692537000	-3.302256000	1.474214000
F	0.374372000	-4.250836000	-1.761037000
F	-0.839638000	-1.982291000	-1.201193000
F	1.016010000	4.848805000	1.474894000
F	-0.362583000	2.663510000	2.040189000
F	2.134195000	0.263874000	-1.202493000
F	3.493297000	2.448614000	-1.762225000
F	-4.707053000	-1.546677000	1.473432000
F	-2.125134000	-1.647050000	2.038657000
F	-1.297198000	1.718579000	-1.200850000
F	-3.868792000	1.802363000	-1.760488000
F	-0.000656000	0.000009000	2.405349000
C	2.054266000	-3.840321000	-0.146496000
C	2.559802000	-2.988969000	0.825566000
C	0.894356000	-3.465129000	-0.804764000
C	0.264761000	-2.271147000	-0.480140000
C	0.725625000	-1.402563000	0.497750000
C	1.902306000	-1.805308000	1.121447000
C	-1.578086000	0.072616000	0.497538000
C	0.851401000	1.329887000	0.497645000
C	1.833514000	1.364678000	-0.480787000
C	0.612962000	2.550314000	1.121737000
C	1.309965000	3.711148000	0.825812000
C	2.553415000	2.506477000	-0.805430000
C	2.299579000	3.698578000	-0.146698000
C	-2.515396000	-0.745054000	1.120957000
C	-2.099700000	0.906520000	-0.480010000
C	-3.448528000	0.958706000	-0.804559000
C	-3.869211000	-0.722263000	0.825091000
C	-4.353576000	0.141725000	-0.146620000
B	-0.000479000	-0.000005000	0.983588000

$(C_6F_4Br)_3BH^-$

anionic, diamagnetic, sum of electronic and zero-point energies= -9632.778722 Hartree

Table S14. Structure optimized coordinates (x,y,z) for $(C_6F_4Br)_3BH^-$

Br	-4.606264000	-4.162714000	-0.478625000
Br	5.912587000	-1.902692000	-0.476195000
Br	-1.310477000	6.068062000	-0.476825000
F	-0.488957000	-2.540802000	2.213297000
F	-2.415227000	-4.280393000	1.669425000
F	-3.951453000	-1.482096000	-1.823460000
F	-2.026023000	0.245970000	-1.299945000
F	4.916508000	0.068504000	1.657218000
F	2.445639000	0.863036000	2.199035000
F	0.801883000	-1.897382000	-1.286330000
F	3.263607000	-2.696739000	-1.808686000
F	-2.517859000	4.219489000	1.657038000
F	-1.968227000	1.682873000	2.199695000
F	1.244029000	1.641350000	-1.285828000
F	0.703821000	4.172471000	-1.808284000
C	-3.236427000	-2.919297000	-0.085206000
C	-2.331151000	-3.155851000	0.939757000
C	-3.105751000	-1.747749000	-0.814792000
C	-2.093673000	-0.847969000	-0.512317000
C	-1.176916000	-1.038860000	0.512808000
C	-1.337239000	-2.229842000	1.213036000
C	-0.308593000	1.538174000	0.512829000
C	1.489595000	-0.502728000	0.513324000
C	1.783363000	-1.399770000	-0.504804000
C	2.601194000	-0.035806000	1.206773000
C	3.900827000	-0.431293000	0.934211000
C	3.069677000	-1.824066000	-0.806703000
C	4.149577000	-1.341321000	-0.083554000
C	-1.268814000	2.267149000	1.206674000
C	0.321298000	2.241662000	-0.504856000
C	0.045070000	3.567746000	-0.806518000
C	-1.577242000	3.590006000	0.933798000
C	-0.913773000	4.260873000	-0.083926000
B	0.001596000	-0.001069000	0.955157000
H	0.001610000	-0.000517000	2.160037000

$B(C_6F_4I)_3$

neutral, diamagnetic, sum of electronic and zero-point energies= -2802.935253 Hartree

Table S15. Structure optimized coordinates (x,y,z) for $B(C_6F_4I)_3$.

I	0.503935000	-6.464454000	-0.000262000
I	5.347570000	3.668180000	-0.000387000
I	-5.851458000	2.795429000	-0.000229000
F	2.148150000	-4.172154000	1.513265000
F	1.950802000	-1.532251000	1.506481000
F	-1.689514000	-1.815742000	-1.506084000
F	-1.475542000	-4.454255000	-1.513325000
F	2.539744000	3.947686000	1.511645000
F	0.351898000	2.457372000	1.505088000
F	2.418037000	-0.556281000	-1.504412000
F	4.596497000	0.947803000	-1.511821000
F	-2.303207000	-0.923770000	1.505416000
F	-4.688327000	0.224633000	1.511931000
F	-3.120323000	3.506402000	-1.511711000
F	-0.728156000	2.372888000	-1.504492000
C	0.342009000	-4.387285000	0.000045000
C	-0.630241000	-3.748279000	-0.760051000
C	1.203543000	-3.605505000	0.760280000
C	1.093412000	-2.225715000	0.743475000
C	-0.735278000	-2.368116000	-0.742894000
C	3.629378000	2.489719000	-0.000065000
C	2.521329000	2.845801000	0.759404000
C	3.562164000	1.327679000	-0.759354000
C	2.419191000	0.546961000	-0.742160000
C	1.381262000	2.060845000	0.742659000
C	1.286521000	0.883055000	0.000357000
C	0.121162000	-1.555036000	0.000443000
C	-1.683780000	1.821918000	-0.742039000
C	-1.407898000	0.673172000	0.000581000
C	-2.475016000	0.165846000	0.742941000
C	-3.725134000	0.760091000	0.759671000
C	-2.931662000	2.420835000	-0.759185000
C	-3.971365000	1.897578000	0.000148000
B	-0.000125000	0.000490000	0.000629000

$(C_6F_4I)_3BF^-$

anionic, diamagnetic, sum of electronic and zero-point energies= -2902.988319 Hartree

Table S16. Structure optimized coordinates (x,y,z) for $(C_6F_4I)_3BF^-$

I	-6.0911840000	-1.9604740000	-0.5159490000
I	4.7461370000	-4.2906860000	-0.5156810000
I	1.3465740000	6.2531380000	-0.5164890000
F	-1.4458440000	-2.2694700000	2.1320660000
F	-3.9085420000	-3.0426720000	1.5698240000
F	-4.2481540000	0.4016250000	-1.6607580000
F	-1.8005390000	1.1839190000	-1.1010640000
F	4.5882340000	-1.8607870000	1.5715180000
F	2.6851970000	-0.1172550000	2.1339640000
F	-0.1243610000	-2.1519770000	-1.1019320000
F	1.7790720000	-3.8781740000	-1.6617120000
F	-0.6730830000	4.9044210000	1.5780300000
F	-1.2348490000	2.3855160000	2.1409910000
F	1.9160740000	0.9648900000	-1.1082900000
F	2.4625190000	3.4756520000	-1.6687670000
F	-0.0011190000	-0.0019120000	2.5023560000
C	-4.1513290000	-1.3316470000	-0.0500790000
C	-3.4012480000	-1.9818580000	0.9193160000
C	-3.5714200000	-0.2572230000	-0.7047410000
C	-2.2833300000	0.1480370000	-0.3817890000
C	-1.5107230000	-0.4634030000	0.5943100000
C	-2.1180860000	-1.5498720000	1.2162110000
C	0.3536730000	1.5379030000	0.5951760000
C	1.1552690000	-1.0769730000	0.5947130000
C	1.0136120000	-2.0514160000	-0.3819730000
C	2.3995170000	-1.0588340000	1.2172710000
C	3.4163380000	-1.9528160000	0.9203430000
C	2.0097670000	-2.9630290000	-0.7049940000
C	3.2298710000	-2.9270990000	-0.0497390000
C	-0.2795030000	2.6073190000	1.2208940000
C	1.2651650000	1.9011680000	-0.3850010000
C	1.5580630000	3.2192150000	-0.7084420000
C	-0.0120500000	3.9345240000	0.9236690000
C	0.9217160000	4.2588180000	-0.0499700000
B	-0.0008290000	-0.0011480000	1.0806520000

$(C_6F_4I)_3BH^-$

anionic, diamagnetic, sum of electronic and zero-point energies= -2803.642506 Hartree

Table S17. Structure optimized coordinates (x,y,z) for $(C_6F_4I)_3BH^-$

I	-6.148732000	-1.810815000	-0.433761000
I	4.646467000	-4.414296000	-0.433263000
I	1.504131000	6.227618000	-0.434410000
F	-1.534884000	-2.090727000	2.283301000
F	-4.021507000	-2.824234000	1.743990000
F	-4.211235000	0.395721000	-1.723835000
F	-1.729405000	1.116872000	-1.201060000
F	4.459286000	-2.060804000	1.739639000
F	2.577525000	-0.277533000	2.278758000
F	-0.105598000	-2.063651000	-1.196576000
F	1.762941000	-3.849193000	-1.718887000
F	-0.429366000	4.891597000	1.749916000
F	-1.036762000	2.371498000	2.289807000
F	1.823510000	0.936788000	-1.206914000
F	2.439629000	3.446854000	-1.730257000
C	-4.187302000	-1.227538000	0.000511000
C	-3.466810000	-1.838317000	1.017246000
C	-3.563280000	-0.221227000	-0.720558000
C	-2.260838000	0.152265000	-0.420610000
C	-1.512675000	-0.424635000	0.596864000
C	-2.169532000	-1.435628000	1.290822000
C	0.388125000	1.519486000	0.597283000
C	1.122214000	-1.098267000	0.596818000
C	0.996890000	-2.037243000	-0.418241000
C	2.327717000	-1.157359000	1.288561000
C	3.326742000	-2.077710000	1.015112000
C	1.973204000	-2.976858000	-0.717956000
C	3.158243000	-3.009740000	0.000799000
C	-0.155771000	2.593597000	1.294088000
C	1.257976000	1.879212000	-0.423371000
C	1.585318000	3.193897000	-0.723677000
C	0.143812000	3.918474000	1.020257000
C	1.029213000	4.237284000	0.000340000
B	-0.000953000	-0.001524000	1.038986000
H	-0.000816000	-0.002063000	2.244471000

$B(C_6F_4Cl)_3$

neutral, diamagnetic, sum of electronic and zero-point energies= -3290.200931 Hartree

Table S18. Structure optimized coordinates (x,y,z) for $B(C_6F_4Cl)_3$.

Cl	0.118855000	6.109967000	0.000260000
Cl	-5.351405000	-2.951794000	0.000302000
Cl	5.232645000	-3.157433000	0.000127000
F	-1.734907000	4.370032000	-1.503022000
F	1.864041000	1.644496000	1.499408000
F	1.903577000	4.299003000	1.503217000
F	-1.798566000	1.716061000	-1.499762000
F	-4.675342000	-0.500369000	1.502662000
F	-0.587180000	-2.416375000	-1.499223000
F	-2.917585000	-3.687991000	-1.502404000
F	-2.356531000	0.792320000	1.498861000
F	4.652284000	-0.682069000	-1.502973000
F	0.492707000	-2.437045000	1.499264000
F	2.772022000	-3.798207000	1.502914000
F	2.385403000	0.699663000	-1.499650000
C	1.335722000	-0.806395000	-0.000236000
C	0.030355000	1.559606000	-0.000139000
C	-1.366127000	-0.753824000	-0.000086000
C	0.964842000	2.285529000	0.739850000
C	-0.875182000	2.321456000	-0.739999000
C	-0.853404000	3.704635000	-0.758654000
C	0.996862000	3.668507000	0.758752000
C	0.085538000	4.396835000	0.000115000
C	-1.573191000	-1.919126000	-0.739689000
C	-2.462100000	-0.307130000	0.739613000
C	-3.675899000	-0.970711000	0.758501000
C	-2.782031000	-2.591707000	-0.758315000
C	-3.851016000	-2.124299000	0.000168000
C	1.497343000	-1.978673000	0.739666000
C	2.448205000	-0.402806000	-0.740037000
C	3.635315000	-1.113032000	-0.758708000
C	2.679149000	-2.697690000	0.758522000
C	3.765496000	-2.272330000	-0.000037000
B	-0.000036000	-0.000262000	-0.000211000

$(C_6F_4Cl)_3BF^-$

anionic, diamagnetic, sum of electronic and zero-point energies= -3390.254739 Hartree

Table S19. Structure optimized coordinates (x,y,z) for $(C_6F_4Cl)_3BF^-$

Cl	5.738378000	-1.894905000	-0.648191000
Cl	-4.512846000	-4.016291000	-0.650220000
Cl	-1.229141000	5.914399000	-0.648684000
F	0.001628000	-0.001193000	2.290885000
F	3.204634000	-2.828675000	-1.872272000
F	0.757846000	-2.021192000	-1.313103000
F	2.506178000	0.977888000	1.921386000
F	4.956317000	0.147929000	1.352688000
F	-4.042161000	-1.361176000	-1.882602000
F	-2.116429000	0.350529000	-1.322470000
F	-0.414463000	-2.656154000	1.929574000
F	-2.361245000	-4.359227000	1.360061000
F	1.367146000	1.662723000	-1.317231000
F	0.842448000	4.185342000	-1.877083000
F	-2.602294000	4.217734000	1.357347000
F	-2.095589000	1.681215000	1.926477000
C	4.137077000	-1.371429000	-0.262348000
C	3.915624000	-0.403743000	0.708586000
C	3.035562000	-1.899308000	-0.918243000
C	1.757187000	-1.467414000	-0.593439000
C	2.624143000	0.001255000	1.004532000
C	-3.257124000	-2.893414000	-0.263691000
C	-3.157493000	-1.678275000	-0.923803000
C	-2.312901000	-3.184579000	0.711884000
C	-1.314875000	-2.270394000	1.008310000
C	-2.143041000	-0.788840000	-0.598320000
C	-1.193844000	-1.033577000	0.382621000
C	1.493176000	-0.518472000	0.382694000
C	-1.310775000	2.270631000	1.007234000
C	-0.297026000	1.550310000	0.383328000
C	0.390293000	2.252445000	-0.595272000
C	0.124684000	3.575255000	-0.920482000
C	-1.606107000	3.591505000	0.711030000
C	-0.881266000	4.266112000	-0.262390000
B	0.001084000	-0.000909000	0.868863000

$(C_6F_4Cl)_3BH^-$

anionic, diamagnetic, sum of electronic and zero-point energies= -3290.908655 Hartree

Table S20. Structure optimized coordinates (x,y,z) for $(C_6F_4Cl)_3BH^-$

Cl	-5.552771000	2.407322000	-0.545924000
Cl	4.862314000	3.601881000	-0.547529000
Cl	0.692324000	-6.011046000	-0.546181000
F	-2.989350000	2.991309000	-1.917206000
F	-0.613792000	1.955666000	-1.393753000
F	-2.517315000	-0.609114000	2.109514000
F	-4.900483000	0.425071000	1.562126000
F	4.079702000	1.094368000	-1.923079000
F	1.993497000	-0.442932000	-1.399154000
F	0.736129000	2.481411000	2.113918000
F	2.824454000	4.026397000	1.565645000
F	-1.389662000	-1.509091000	-1.391438000
F	-1.098216000	-4.084229000	-1.914969000
F	2.085033000	-4.454039000	1.559800000
F	1.788251000	-1.873257000	2.107499000
C	-3.999164000	1.739840000	-0.185094000
C	-3.838264000	0.814613000	0.837997000
C	-2.876077000	2.108647000	-0.911380000
C	-1.638107000	1.560746000	-0.608618000
C	-2.583925000	0.294414000	1.111539000
C	3.506569000	2.591467000	-0.186315000
C	3.261192000	1.436484000	-0.914812000
C	2.627445000	2.913741000	0.839275000
C	1.549034000	2.088558000	1.113121000
C	2.167067000	0.639387000	-0.611748000
C	1.273408000	0.918523000	0.413394000
C	-1.432630000	0.644530000	0.413966000
C	1.038123000	-2.383131000	1.110445000
C	0.157929000	-1.561704000	0.413952000
C	-0.533981000	-2.198234000	-0.607534000
C	-0.389252000	-3.544240000	-0.910288000
C	1.215188000	-3.729468000	0.836759000
C	0.493234000	-4.331903000	-0.185209000
B	-0.000487000	0.000600000	0.856950000
H	-0.000251000	0.000699000	2.062210000

$B(C_6F_5)_3$

neutral, diamagnetic, sum of electronic and zero-point energies= -2209.137780 Hartree

Table S21. Structure optimized coordinates (x,y,z) for $B(C_6F_5)_3$.

F	1.565224000	-1.936794000	-1.495627000
F	-2.449664000	-4.034444000	1.494148000
F	-2.067462000	-1.386713000	1.494870000
F	-0.853360000	-5.644225000	0.000668000
F	1.147460000	-4.579217000	-1.493518000
F	-0.167549000	2.484252000	1.494907000
F	-4.539568000	1.295408000	-1.493425000
F	-2.459869000	-0.387403000	-1.495257000
F	-4.461755000	3.560886000	0.000460000
F	-2.269570000	4.138839000	1.493930000
F	0.894851000	2.324013000	-1.495500000
F	4.718987000	-0.104320000	1.494074000
F	2.234851000	-1.097182000	1.494729000
F	3.392149000	3.283431000	-1.493411000
F	5.314925000	2.083130000	0.000719000
C	-0.654975000	-4.332444000	0.000328000
C	-1.472353000	-3.505695000	0.759283000
C	0.370254000	-3.784748000	-0.758999000
C	0.569193000	-2.415459000	-0.738446000
C	-1.257596000	-2.138816000	0.737932000
C	-3.424766000	2.733379000	0.000121000
C	-3.462911000	1.571556000	-0.759057000
C	-2.300131000	3.028082000	0.759062000
C	-1.223654000	2.158786000	0.737798000
C	-2.376425000	0.714770000	-0.738446000
C	4.079712000	1.599015000	0.000334000
C	3.772425000	0.477722000	0.759229000
C	3.092761000	2.213054000	-0.758968000
C	1.807450000	1.700701000	-0.738421000
C	2.481288000	-0.019721000	0.737858000
C	-1.219269000	0.973366000	-0.000566000
C	1.452616000	0.569383000	-0.000474000
C	-0.233145000	-1.542505000	-0.000480000
B	0.000060000	0.000099000	-0.000886000

$(C_6F_5)_3BF^-$

anionic, diamagnetic, sum of electronic and zero-point energies= -2309.190293 Hartree

Table S22. Structure optimized coordinates (x,y,z) for $(C_6F_5)_3BF^-$

F	-0.000104000	-0.000160000	2.223519000
F	-5.070506000	2.497130000	-0.632676000
F	-4.277929000	0.245757000	-1.951150000
F	-1.108516000	2.448888000	1.861501000
F	-3.456039000	3.577831000	1.279459000
F	-1.951586000	-0.907560000	-1.389143000
F	-1.376963000	-4.780015000	1.275395000
F	0.370986000	-5.640023000	-0.631356000
F	1.768041000	-1.238419000	-1.383518000
F	1.930820000	-3.829914000	-1.944989000
F	-1.571575000	-2.182160000	1.856746000
F	2.351763000	3.585176000	-1.947597000
F	4.698615000	3.142307000	-0.631770000
F	2.675886000	-0.267988000	1.858035000
F	4.827690000	1.200547000	1.277032000
F	0.189355000	2.147975000	-1.386007000
C	1.299180000	0.899991000	0.315234000
C	-1.428584000	0.675294000	0.314968000
C	0.129758000	-1.574735000	0.315219000
C	-1.872917000	1.836456000	0.940223000
C	-2.278000000	0.184777000	-0.665653000
C	-3.493127000	0.771503000	-0.994490000
C	-3.077875000	2.454994000	0.644312000
C	-3.899495000	1.914584000	-0.330814000
C	0.981792000	-2.066191000	-0.662569000
C	-0.656682000	-2.539183000	0.938229000
C	-0.590624000	-3.892061000	0.642646000
C	1.080587000	-3.412064000	-0.991037000
C	0.290638000	-4.334513000	-0.329806000
C	2.527414000	0.702345000	0.939052000
C	1.298933000	1.882378000	-0.663876000
C	2.414881000	2.641122000	-0.992456000
C	3.665752000	1.436379000	0.643432000
C	3.608376000	2.419738000	-0.330093000
B	0.000101000	0.000150000	0.800712000

$(C_6F_5)_3BH^-$

anionic, diamagnetic, sum of electronic and zero-point energies= -2209.843552 Hartree

Table S23. Structure optimized coordinates (x,y,z) for $(C_6F_5)_3BH^-$

F	1.425594000	3.969763000	-2.010293000
F	1.483987000	1.358706000	-1.478753000
F	-1.571784000	2.040271000	2.083783000
F	-1.630815000	4.653154000	1.514032000
F	2.731388000	-3.246405000	-1.986668000
F	0.438964000	-1.994994000	-1.460341000
F	2.563818000	0.360234000	2.066376000
F	4.857172000	-0.896418000	1.504156000
F	-1.897362000	0.590065000	-1.496540000
F	-4.138592000	-0.749701000	-2.028877000
F	-3.251864000	-3.715345000	1.527870000
F	-1.005474000	-2.376749000	2.095065000
F	-0.132928000	5.652589000	-0.534762000
F	4.971484000	-2.716398000	-0.524298000
F	-4.848325000	-2.918780000	-0.535259000
C	-0.096540000	4.340389000	-0.249235000
C	-0.854197000	3.827180000	0.789919000
C	0.696267000	3.482223000	-0.990928000
C	0.716288000	2.129418000	-0.680641000
C	-0.798220000	2.469810000	1.067318000
C	3.815969000	-2.092250000	-0.241431000
C	2.675020000	-2.359636000	-0.977144000
C	3.751600000	-1.167642000	0.787385000
C	2.547382000	-0.537610000	1.061489000
C	1.492447000	-1.700953000	-0.670000000
C	1.371061000	-0.776800000	0.359081000
C	-0.013685000	1.567015000	0.358213000
C	-1.756344000	-1.921773000	1.072515000
C	-1.352404000	-0.800459000	0.355701000
C	-2.194180000	-0.451793000	-0.691797000
C	-3.360828000	-1.136906000	-1.002280000
C	-2.909867000	-2.639692000	0.795914000
C	-3.724038000	-2.240698000	-0.250863000
B	0.001611000	-0.004696000	0.802832000
H	0.000187000	-0.005386000	2.007120000

$B(C_6F_4CF_3)_3$

neutral, diamagnetic, sum of electronic and zero-point energies= -2922.854773 Hartree

Table S24. Structure optimized coordinates (x,y,z) for $B(C_6F_4CF_3)_3$.

F	-1.8424640000	1.6326570000	-1.5425650000
F	1.6928790000	4.3639130000	1.5066920000
F	1.7674080000	1.7196680000	1.5083700000
F	-1.9121810000	4.2488160000	-1.5658420000
F	0.6055090000	-2.3947100000	1.5038470000
F	4.6374720000	-0.4652960000	-1.5634050000
F	2.3368020000	0.7811010000	-1.5401930000
F	2.9335010000	-3.6509490000	1.5023170000
F	-0.4932970000	-2.4143870000	-1.5408560000
F	-4.6276920000	-0.7133840000	1.5038880000
F	-2.3747620000	0.6729320000	1.5049850000
F	-2.7242490000	-3.7820440000	-1.5637800000
F	6.1238210000	-2.3709080000	-0.7479720000
F	4.9733670000	-4.1621680000	-0.3709300000
F	5.6460900000	-2.9301870000	1.2850760000
F	-5.1166650000	-4.1157000000	-0.7477330000
F	-6.0916710000	-2.2231120000	-0.3711800000
F	-5.3626400000	-3.4221120000	1.2852450000
F	-1.0046530000	6.4872530000	-0.7516540000
F	1.1206920000	6.3864280000	-0.3678340000
F	-0.2880100000	6.3542510000	1.2839050000
C	-0.0788180000	5.9226120000	0.0269580000
C	5.1702540000	-2.8923420000	0.0272850000
C	-5.0911430000	-3.0291760000	0.0275550000
C	-0.1050720000	4.4076180000	-0.0244980000
C	0.8201910000	3.7043980000	0.7464600000
C	-0.9976690000	3.6671950000	-0.7941010000
C	-0.9588160000	2.2797000000	-0.7720170000
C	0.8550690000	2.3228780000	0.7362200000
C	3.8711230000	-2.1124550000	-0.0247880000
C	3.6763760000	-0.9681280000	-0.7929200000
C	2.7988610000	-2.5639590000	0.7441550000
C	1.5846470000	-1.9040620000	0.7337540000
C	2.4549510000	-0.3087080000	-0.7710550000
C	-3.7656480000	-2.2949290000	-0.0240370000
C	-3.6195800000	-1.1409460000	0.7453480000
C	-2.6778040000	-2.6987950000	-0.7926880000
C	-1.4954700000	-1.9716530000	-0.7708950000
C	-2.4404270000	-0.4202370000	0.7348540000
C	1.3701930000	-0.7496860000	-0.0189530000
C	-1.3339090000	-0.8122700000	-0.0181820000
C	-0.0362120000	1.5604100000	-0.0182430000
B	0.0000170000	-0.0006670000	-0.0189380000

$(C_6F_4CF_3)_3BF^–$

anionic, diamagnetic, sum of electronic and zero-point energies= -3022.925895 Hartree

Table S25. Structure optimized coordinates (x,y,z) for $(C_6F_4CF_3)_3BF^–$

F	1.3403140000	1.6836680000	-1.2308400000
F	4.6567300000	-1.6599880000	1.4368240000
F	2.0803920000	-1.7055240000	1.9975850000
F	3.8761000000	1.7244430000	-1.7920090000
F	-2.5185770000	-0.9463690000	1.9949170000
F	-0.4430460000	-4.2211730000	-1.7890440000
F	0.7904000000	-2.0050650000	-1.2279280000
F	-3.7680350000	-3.2000590000	1.4344840000
F	-2.1281600000	0.3184800000	-1.2306290000
F	-0.8906290000	4.8624930000	1.4369130000
F	0.4368330000	2.6540520000	1.9978520000
F	-3.4313200000	2.4941990000	-1.7919160000
F	-2.3688600000	-5.8496130000	-1.3856730000
F	-4.1838130000	-4.7869390000	-0.8847590000
F	-3.0655930000	-5.8175590000	0.6602120000
F	-3.8806460000	4.9757900000	-1.3876030000
F	-2.0543140000	6.0170350000	-0.8830540000
F	-3.5080250000	5.5617970000	0.6593200000
F	6.2497990000	0.8729170000	-1.3877080000
F	6.2386260000	-1.2292960000	-0.8829450000
F	6.5709910000	0.2574950000	0.6592920000
F	0.0001760000	-0.0002280000	2.3662030000
C	5.8509150000	-0.0055470000	-0.4555450000
C	-2.9310350000	-5.0636170000	-0.4551370000
C	-2.9203540000	5.0695320000	-0.4555180000
C	4.3742420000	0.0410280000	-0.1806730000
C	3.8430870000	-0.8114200000	0.7871850000
C	3.4743480000	0.8763810000	-0.8339490000
C	2.1230230000	0.8526050000	-0.5116610000
C	2.4942280000	-0.8112840000	1.0852800000
C	-2.1518370000	-3.8084000000	-0.1802590000
C	-0.9773680000	-3.4480580000	-0.8323350000
C	-2.6252480000	-2.9210400000	0.7861850000
C	-1.9503860000	-1.7531170000	1.0841000000
C	-0.3219510000	-2.2660180000	-0.5102010000
C	-2.2225030000	3.7673430000	-0.1805710000
C	-1.2186860000	3.7335700000	0.7872780000
C	-2.4960160000	2.5703240000	-0.8338460000
C	-1.7997800000	1.4119280000	-0.5115340000
C	-0.5443730000	2.5653600000	1.0853830000
C	-0.7620600000	-1.3825770000	0.4609300000
C	-0.8159890000	1.3512410000	0.4609690000
C	1.5785620000	0.0310230000	0.4608910000
B	0.0001990000	-0.0001130000	0.9465440000

$(C_6F_4CF_3)_3BH^-$

anionic, diamagnetic, sum of electronic and zero-point energies= -2923.581192 Hartree

Table S26. Structure optimized coordinates (x,y,z) for $(C_6F_4CF_3)_3BH^-$

F	1.5222090000	1.4426660000	-1.2909010000
F	4.4350150000	-2.0968610000	1.5908230000
F	1.8601060000	-1.8214340000	2.1227740000
F	4.0603990000	1.1880090000	-1.8113140000
F	-2.5091290000	-0.6953510000	2.1185240000
F	-0.9999220000	-4.1142270000	-1.8067970000
F	0.4914590000	-2.0446270000	-1.2870660000
F	-4.0369430000	-2.7864100000	1.5870690000
F	-2.0043400000	0.5970230000	-1.2955240000
F	-0.4061850000	4.8871680000	1.5953450000
F	0.6424900000	2.5196110000	2.1278930000
F	-3.0528090000	2.9224160000	-1.8164450000
F	-3.0783140000	-5.5217520000	-1.3353800000
F	-4.7404650000	-4.2854250000	-0.7162150000
F	-3.6527670000	-5.4569190000	0.7476380000
F	-3.2378090000	5.4253800000	-1.3429740000
F	-1.3420710000	6.2490020000	-0.7087750000
F	-2.9096960000	5.8860920000	0.7435030000
F	6.3202630000	0.0959400000	-1.3381550000
F	6.0836290000	-1.9603800000	-0.7135290000
F	6.5530190000	-0.4282770000	0.7461180000
C	5.8200300000	-0.6795860000	-0.3642410000
C	-3.5006960000	-4.6985580000	-0.3637240000
C	-2.3203230000	5.3790810000	-0.3650940000
C	4.3545490000	-0.4634860000	-0.1184590000
C	3.7227050000	-1.2065480000	0.8794410000
C	3.5570000000	0.4342360000	-0.8223880000
C	2.2070810000	0.5682840000	-0.5254640000
C	2.3789880000	-1.0464490000	1.1523440000
C	-2.5797790000	-3.5382580000	-0.1180980000
C	-1.4018770000	-3.2991410000	-0.8200650000
C	-2.9081420000	-2.6173820000	0.8776110000
C	-2.0967330000	-1.5343750000	1.1502300000
C	-0.6099410000	-2.1976260000	-0.5234930000
C	-1.7747920000	4.0019050000	-0.1190650000
C	-0.8182830000	3.8256910000	0.8815230000
C	-2.1510480000	2.8628130000	-0.8250640000
C	-1.5924190000	1.6267440000	-0.5277500000
C	-0.2852590000	2.5819110000	1.1547660000
C	-0.9090820000	-1.2788830000	0.4705780000
C	-0.6521280000	1.4263220000	0.4709930000
C	1.5624570000	-0.1484950000	0.4707680000
B	0.0005060000	-0.0005780000	0.9087520000
H	0.0003770000	-0.0011130000	2.1146510000

$B(C_6F_4H)_3$

neutral, diamagnetic, sum of electronic and zero-point energies= -1911.313377 Hartree

Table S27. Structure optimized coordinates (x,y,z) for $B(C_6F_4H)_3$.

F	2.2333470000	1.0362370000	1.5310470000
F	-0.3720750000	4.6821660000	-1.5287030000
F	-1.2072270000	2.1463460000	-1.5316380000
F	3.0368900000	3.5822960000	1.5291640000
F	-1.2548310000	-2.1173200000	-1.5330680000
F	-4.6211240000	0.8369790000	1.5301320000
F	-2.0144780000	1.4147160000	1.5322960000
F	-3.8684700000	-2.6627150000	-1.5304620000
F	-0.2178760000	-2.4509840000	1.5333830000
F	4.2397440000	-2.0193130000	-1.5303170000
F	2.4608470000	-0.0283130000	-1.5331960000
F	1.5855990000	-4.4197450000	1.5315470000
C	1.3478590000	4.1802060000	0.0002700000
C	0.2688040000	3.7854640000	-0.7685620000
C	1.9929380000	3.2291890000	0.7688450000
C	1.5719350000	1.9100220000	0.7575930000
C	-0.1601630000	2.4688650000	-0.7578160000
C	-4.2941000000	-0.9234670000	-0.0001460000
C	-3.7932300000	0.1102150000	0.7692070000
C	-3.4125740000	-1.6597900000	-0.7695920000
C	-2.0579590000	-1.3726540000	-0.7586950000
C	-2.4403590000	0.4055330000	0.7581000000
C	2.9464320000	-3.2569630000	0.0005090000
C	3.1432880000	-2.1256630000	-0.7693430000
C	1.8009140000	-3.3397270000	0.7700770000
C	0.8687940000	-2.3157360000	0.7587430000
C	2.2173620000	-1.0960240000	-0.7586560000
C	-1.5264950000	-0.3280960000	-0.0003470000
C	1.0471310000	-1.1577560000	-0.0001740000
C	0.4789810000	1.4858870000	-0.0002090000
B	-0.0001760000	0.0000280000	-0.0003360000
H	1.6792230000	5.2077810000	0.0004610000
H	-5.3496310000	-1.1505490000	-0.0000720000
H	3.6708150000	-4.0575610000	0.0007980000

$(C_6F_4H)_3BF^-$

anionic, diamagnetic, sum of electronic and zero-point energies= -2011.357607 Hartree

Table S28. Structure optimized coordinates (x,y,z) for $(C_6F_4H)_3BF^-$

F	-0.1830440000	0.0675930000	2.2121830000
F	5.1836680000	-2.2733340000	-0.1982100000
F	4.3739110000	-0.1155380000	-1.6470520000
F	1.0479370000	-2.3522630000	2.0019620000
F	1.9643360000	0.9255980000	-1.2898900000
F	0.9116230000	4.8754750000	1.1227020000
F	-1.7328370000	0.9914580000	-1.5988010000
F	-2.0241310000	3.5262610000	-2.3129870000
F	1.2339970000	2.3400920000	1.8591060000
F	-2.1006590000	-3.8513390000	-1.8569850000
F	-2.8408340000	0.1982280000	1.6757480000
F	-4.8761540000	-1.4042840000	1.0675700000
F	-0.0524110000	-2.2785380000	-1.2689760000
C	-1.3228810000	-0.9910450000	0.2861890000
C	1.3890440000	-0.6211930000	0.4277340000
C	-0.2823630000	1.5359590000	0.2139940000
C	1.8613040000	-1.7379470000	1.1141680000
C	2.2863890000	-0.1239680000	-0.5055370000
C	3.5531070000	-0.6564210000	-0.7264180000
C	3.1084270000	-2.3156020000	0.9443670000
C	3.9554600000	-1.7523020000	0.0103630000
C	-1.0758580000	1.9142480000	-0.8608890000
C	0.3797600000	2.5849070000	0.8475510000
C	0.2372270000	3.9076900000	0.4611840000
C	-1.2280520000	3.2343500000	-1.2593770000
C	-0.5761300000	4.2564870000	-0.6001080000
C	-2.5948220000	-0.8177840000	0.8266950000
C	-1.2167940000	-2.0299240000	-0.6288320000
C	-2.2821900000	-2.8542000000	-0.9612340000
C	-3.6679060000	-1.6322310000	0.5041070000
C	-3.5276640000	-2.6716690000	-0.3956600000
B	-0.0962010000	-0.0025760000	0.7918410000
H	3.4056610000	-3.1839690000	1.5122650000
H	-0.6897170000	5.2848950000	-0.9064550000
H	-4.3598950000	-3.3088460000	-0.6521380000

$(C_6F_4H)_3BH^-$

anionic, diamagnetic, sum of electronic and zero-point energies= -1912.010966 Hartree

Table S29. Structure optimized coordinates (x,y,z) for $(C_6F_4H)_3BH^-$

F	5.1911860000	-2.2208460000	-0.0673670000
F	4.2303720000	-0.3368990000	-1.7822450000
F	1.1820300000	-2.0575260000	2.3621750000
F	1.7906500000	0.6694310000	-1.4557240000
F	0.8872790000	4.8550620000	1.2766600000
F	-1.6768550000	1.0230150000	-1.5959720000
F	-1.9297450000	3.5758280000	-2.2853580000
F	1.1422110000	2.3012930000	2.0037460000
F	-2.1640640000	-3.7918080000	-1.8430610000
F	-2.7844890000	0.1222340000	1.8648330000
F	-4.8379710000	-1.4763420000	1.2792070000
F	-0.1087510000	-2.2037930000	-1.2937020000
C	-1.3301080000	-0.9790090000	0.3448870000
C	1.3672700000	-0.6103190000	0.5016690000
C	-0.2813480000	1.5334290000	0.2649960000
C	1.9184780000	-1.5864730000	1.3267170000
C	2.2013220000	-0.2342760000	-0.5430320000
C	3.4756220000	-0.7523310000	-0.7464850000
C	3.1768650000	-2.1478900000	1.1796840000
C	3.9544360000	-1.7112200000	0.1253530000
C	-1.0400920000	1.9346240000	-0.8283390000
C	0.3565140000	2.5712920000	0.9398120000
C	0.2388070000	3.9005400000	0.5695460000
C	-1.1719890000	3.2614770000	-1.2094820000
C	-0.5340880000	4.2705130000	-0.5150890000
C	-2.5784080000	-0.8473280000	0.9484070000
C	-1.2504080000	-1.9937080000	-0.6018100000
C	-2.3180220000	-2.8236660000	-0.9103430000
C	-3.6552310000	-1.6644720000	0.6487460000
C	-3.5419210000	-2.6742130000	-0.2885510000
B	-0.1117170000	0.0033950000	0.8113200000
H	-0.1963330000	0.0773510000	2.0117410000
H	3.5390080000	-2.9019540000	1.8617930000
H	-0.6306880000	5.3038220000	-0.8102920000
H	-4.3762720000	-3.3148390000	-0.5286960000

Crystallographic Data

Table S30. Crystallographic data of [2($AgC_6F_4Br)_4$•7.46(toluene)].

Identification code	2469940
Empirical formula	$C_{12.51}H_{7.45}AgBrF_4$
Formula weight	421.59
Temperature/K	100(2)
Crystal system	triclinic
Space group	$P\bar{1}$
a/Å	14.0505(12)
b/Å	14.9314(15)
c/Å	27.051(3)
α/°	85.576(3)
β/°	75.456(3)
γ/°	65.540(3)
Volume/Å^3	4997.9(8)
Z	16
ρ_{calc} g/cm^3	2.241
μ/mm^{-1}	4.839
F(000)	3209.0
Crystal size/mm^3	0.164 × 0.145 × 0.048
Radiation	MoK$_\alpha$ (λ = 0.71073)
2Θ range for data collection/°	4.38 to 54.06
Index ranges	-18 ≤ h ≤ 16, -19 ≤ k ≤ 19, -34 ≤ l ≤ 34
Reflections collected	106659
Independent reflections	22031 [R_{int} = 0.0676, R_{sigma} = 0.0548]
Data/restraints/parameters	22031/471/1481
Goodness-of-fit on F^2	1.092
Final R indexes [I>=2σ (I)]	R_1 = 0.0674, wR_2 = 0.1757
Final R indexes [all data]	R_1 = 0.0899, wR_2 = 0.1907
Largest diff. peak/hole / e Å^{-3}	2.57/-1.91

The measured crystal was twinned with three domains, but with a low percentage of the other two domains, so that twin integration didn't improve the structure and wasn't performed. The co-crystallized toluene molecules were disordered and restraints RIGU/ISOR were used to obtain suitable ellipsoids. Also constraint AFIX 66 was used to get planar well-formed benzene rings for the toluene molecules. One toluene unit had an occupancy of 0.45 and the disorder couldn't be solved, therefor it was squeezed.

Table S31. Selected structural data of [2($AgC_6F_4Br)_4$•7.46(toluene)] (*d* in Å, angles in °).

		[2($AgC_6F_4Br)_4$•7.46(toluene)]
$(Ag)_4$-tetramers	*d*(Ag–Ag)	2.756(1) – 2.773(1)
	d(C_{Phenyl}–Ag)	2.209(9) – 2.294(7)
	Torsion angles Ag_4	28.2, 30.2
	Angles Ag_4	83.7 – 88.0
π-complexation	*d*($C_{toluene}$–Ag)	2.673(44) – 3.355(39)
Halogen contacts	*d*(F–F)	2.619(11) – 2.923(8)
	d(Br–F)	3.031(8) – 3.289(8)
π-stacking	*d*(C–C)	3.190(12) – 3.393(12)
Carbon–Halogen contacts	*d*(C–F)	2.97(3) – 3.158(29)
	d(C–Br)	3.4
Hydrogen Bonding	*d*(H–F)	2.261(5) – 2.669(8)

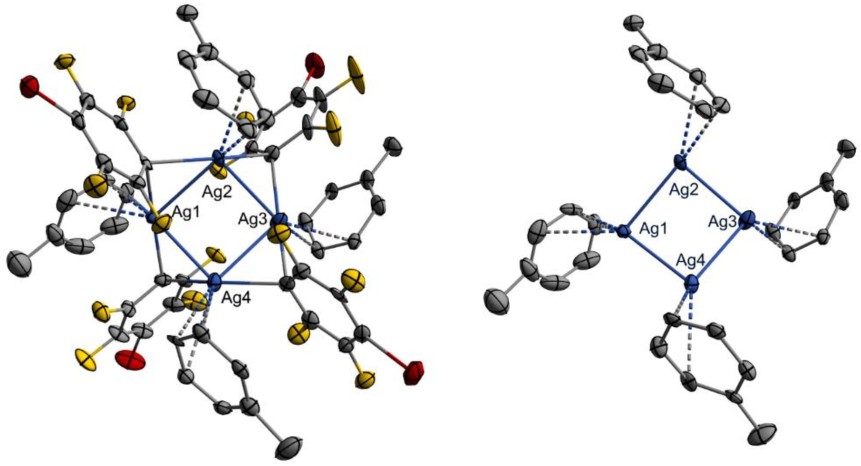

Figure S35. Tetramer A [$AgC_6F_4Br]_4$ with C_6F_4Br-ligands and all coordinating toluene molecules (left) and tetramer A without C_6F_4Br-ligands only showing π-π-complexation of the toluenes (right). Ellipsoids are depicted with 50% probability level and hydrogen atoms as well as disorder are omitted for clarity. Color code: blue – silver, grey – carbon, yellow – fluorine, red – bromine.

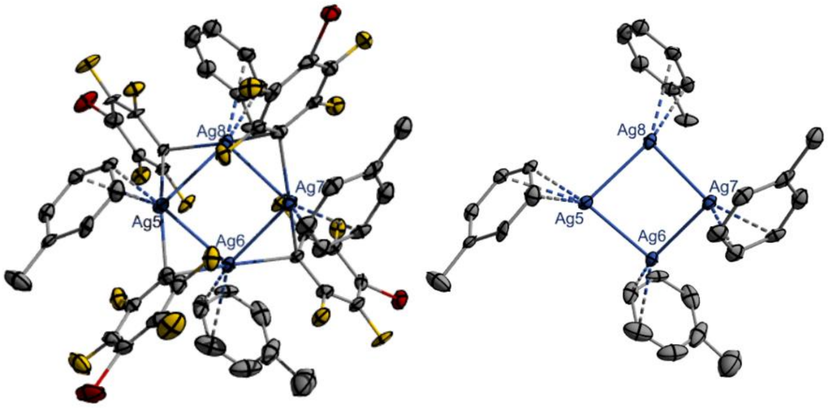

Figure S36. Tetramer B $[AgC_6F_4Br]_4$ with C_6F_4Br-ligands and all coordinating toluene molecules (left) and tetramer B without C_6F_4Br-ligands only showing π-π-complexation of the toluenes (right). Ellipsoids are depicted with 50% probability level and hydrogen atoms as well as disorder are omitted for clarity. Color code: blue – silver, grey – carbon, yellow – fluorine, red – bromine.

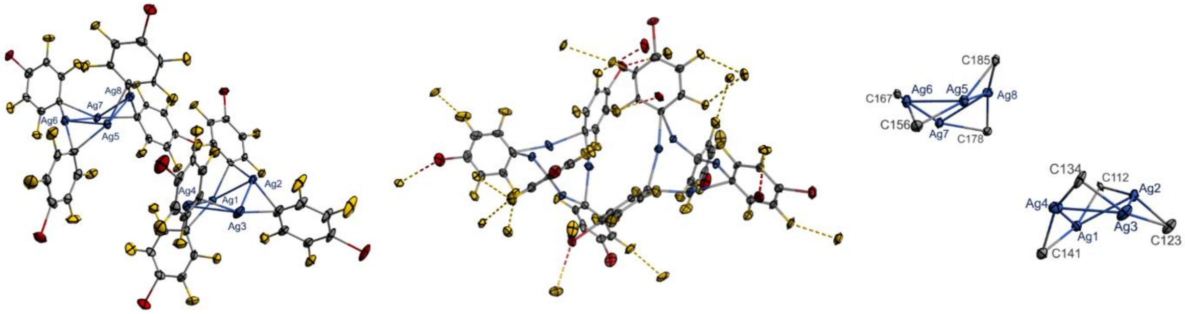

Figure S37. Both tetramers $[AgC_6F_4Br]_4$ A and B with C_6F_4Br-ligands (left, toluene molecules omitted), both tetramers shown with fluorine–fluorine and fluorine–bromine contacts (middle, toluene omitted) and the geometry of Ag_4C_4-units of both tetramers A and B (right, toluene molecules and remaining C_6F_4Br omitted for clarity). Ellipsoids are depicted with 50% probability level. Color code: blue – silver, grey – carbon, yellow – fluorine, red – bromine.

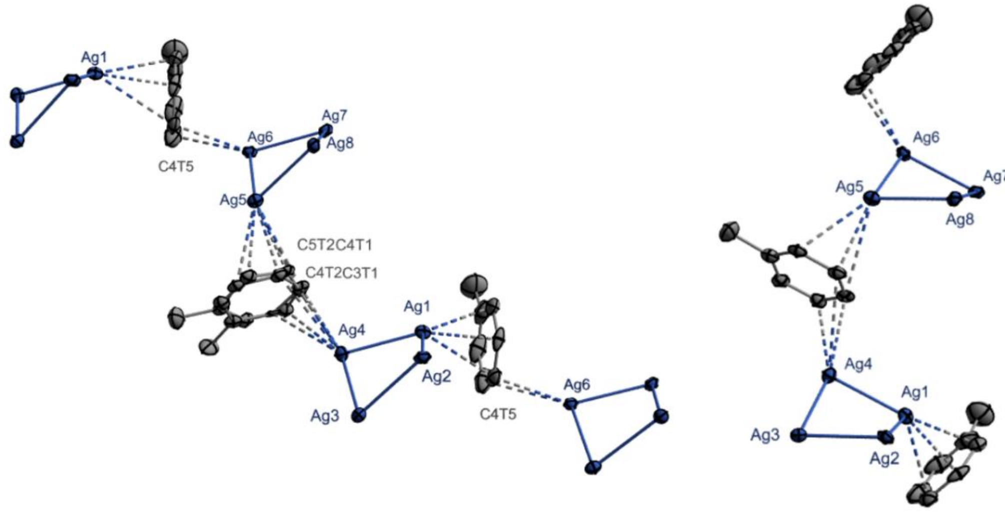

Figure S38. Chain structure of toluene bridged tetramers A and B $[AgC_6F_4Br]_4$ (left, hydrogen atoms, non-bridging toluene molecules and C_6F_4Br omitted for clarity) and bridged tetramers A and B with all coordinating toluene molecules (right; disorder, hydrogen atoms and C_6F_4Br omitted for clarity). Ellipsoids are depicted with 50% probability level. Color code: blue – silver, grey – carbon.

Table S32: Selected bond lengths of [2($AgC_6F_4Br)_4$•7.46(toluene)] in Å.

Ag–C_6F_4Br	
Ag1–C141	2.2088(91)
Ag8–C185	2.2108(79)
Ag6–C167	2.2150(83)
Ag3–C123	2.2198(138)
Ag4–C134	2.2256(95)
Ag5–C156	2.2306(100)
Ag8–C178	2.2328(81)
Ag2–C112	2.2423(88)
Ag7–C178	2.2429(113)
Ag1–C112	2.2559(74)
Ag6–C156	2.2599(90)
Ag4–C141	2.2775(86)
Ag7–C167	2.2796(113)
Ag3–C134	2.2807(131)
Ag2–C123	2.2874(114)
Ag5–C185	2.2937(73)

Ag–$C_{Toluene}$	
Ag1–C5T5	2.7475(72)
Ag1–C6T5	3.1604(68)
Ag1–C4T5	3.2421(76)
Ag2–C6T3	2.7869(96)
Ag2–C5T3	2.8277(85)
Ag3–C4T8	2.7574(225)
Ag3–C3T8	2.9030(207)
Ag3–C4T7	2.8592(220)
Ag3–C3T7	3.0431(200)
Ag4–C3T1	2.7681(441)
Ag4–C2T1	3.1488(345)
Ag4–C4T1	3.2583(291)
Ag4–C4T2	2.6730(437)
Ag4–C3T2	2.9759(346)
Ag4–C5T2	3.3131(414)
Ag5–C4T1	2.6753(318)
Ag5–C5T1	2.8206(278)
Ag5–C3T1	3.3549(386)
Ag5–C5T2	2.7635(411)
Ag5–C6T2	3.0432(265)
Ag5–C4T2	3.2894(400)
Ag6–C4T5	2.8160(89)
Ag6–C3T5	3.0992(93)
Ag7–C3T4	2.7306(89)
Ag7–C4T4	2.7757(81)
Ag8–C2T6	2.9551(74)
Ag8–C3T6	3.0746(90)

Ag–Ag	
Ag3–Ag4	2.7557(14)
Ag1–Ag4	2.7589(11)
Ag5–Ag6	2.7635(12)
Ag2–Ag3	2.7663(10)
Ag5–Ag8	2.7673(8)
Ag6–Ag7	2.768(1)
Ag8–Ag7	2.7701(11)
Ag2–Ag1	2.7730(11)

F–F	
F241–F323	2.6185(111)
F323–F241	2.6185(111)
F385–F267	2.7241(108)
F123–F441	2.777(10)
F123–F441	2.777(10)
F285–F412	2.7873(90)
F412–F285	2.7873(90)
F123–F156	2.8881(74)
F156–F123	2.8881(74)
F185–F412	2.9167(75)
F412–F185	2.9167(75)
F485–F212	2.9226(80)
F267–F385	2.7241(108)

Br–F	
Br23–F223	3.0313(79)
Br85–F285	3.0317(67)
Br78–F378	3.0333(57)
Br12–F212	3.0387(61)
Br56–F356	3.0428(85)
Br41–F341	3.0432(98)
Br67–F267	3.0433(51)
Br56–F256	3.0456(66)
Br34–F234	3.0459(53)
Br67–F367	3.0601(64)
Br12–F312	3.0620(71)
Br85–F385	3.0640(71)
Br41–F241	3.0660(74)
Br78–F278	3.0686(67)
Br23–F323	3.0723(88)
Br34–F334	3.0754(61)
Br23–F334	3.2219(76)
Br34–F234	3.2890(78)
Br56–F341	3.0584(74)
Br78–F367	3.2762(55)

Br–C_6F_4Br	
Br78–C478	1.866(9)
Br67–C467	1.871(9)
Br12–C412	1.886(8)
Br85–C485	1.867(10)
Br34–C434	1.872(9)
Br23–C423	1.867(10)
Br56–C456	1.875(10)
Br41–C441	1.883(10)

C–C contacts	
C323–C385	3.1904(116)
C578–C312	3.2714(125)
C656–C3T2	3.2756(345)
C578–C6T4	3.2831(140)
C678–C6T4	3.3065(116)
C312–C3T3	3.3097(139)
C1T8–C523	3.3137(258)
C6T8–C323	3.3150(242)
C5T8–C223	3.3218(256)
C5T1–C634	3.3282(331)
C556–C3T2	3.3303(364)
C641–C3T5	3.3352(131)
C567–C6T5	3.3354(115)
C585–C5T6	3.3384(152)
C656–C2T1	3.3515(336)
C556–C2T1	3.3553(354)
C412–C2T3	3.3618(134)
C7T8–C523	3.3671(267)
C534–C6T2	3.3672(285)
C656–C3T1	3.3751(436)
C541–C3T5	3.3765(143)
C2T9–C3T8	3.3774(260)
C434–C6T1	3.3929(283)
C634–C5T2	3.3929(384)
C5T5–C667	3.3930(122)

Table S33. Crystallographic data of $B(C_6F_4Br)_3$.

Identification code	2469941
Empirical formula	$C_{18}BF_{12}Br_3$
Formula weight	694.72
Temperature/K	100.00
Crystal system	trigonal
Space group	$R\bar{3}$
a/Å	42.5459(11)
b/Å	42.5459(11)
c/Å	5.6797(2)
α/°	90
β/°	90
γ/°	120
Volume/Å^3	8903.7(6)
Z	18
ρ_{calc} g/cm^3	2.332
μ/mm^{-1}	8.700
F(000)	5868.0
Crystal size/mm^3	0.184 × 0.106 × 0.096
Radiation	CuK_α (λ = 1.54178)
2Θ range for data collection/°	7.20 to 130.036
Index ranges	-49 ≤ h ≤ 23, 0 ≤ k ≤ 50, 0 ≤ l ≤ 6
Reflections collected	3489
Independent reflections	3489 [R_{int} = 0.0468, R_{sigma} = 0.0449]
Data/restraints/parameters	3489/0/308
Goodness-of-fit on F^2	1.108
Final R indexes [I>=2σ (I)]	R_1 = 0.0705, wR_2 = 0.1811
Final R indexes [all data]	R_1 = 0.0823, wR_2 = 0.1978
Largest diff. peak/hole / e Å^{-3}	1.45/-2.07

The measured crystal was twinned with a percentage of domain 1 of 58%, so that a twin integration was performed. The solved structure was then refined with the hkl5-file. The ellipsoids of the bromine atoms that are interacting with each other are a little bit distorted, but it was not possible to treat this as disorder. The phenyl substituents are named as phenyl **A**, **B** and **C**, according to the depicted bromine labels in Fig. 39.

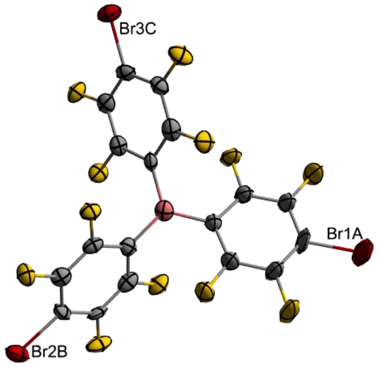

Figure S39. Molecular structure of $B(C_6F_4Br)_3$ in the solid state. Ellipsoids are depicted with 50% probability level. Color code: pink – boron, grey – carbon, yellow – fluorine, red –bromine.

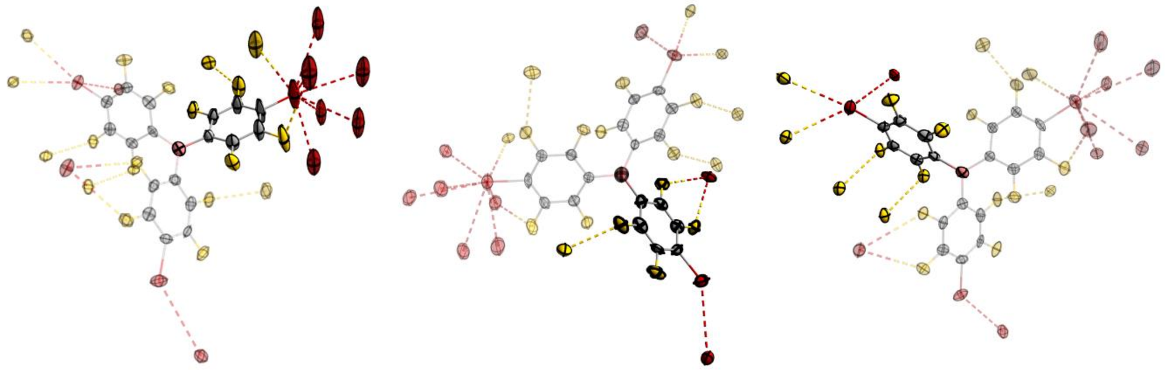

Figure S40. Intermolecular halogen-contacts between fluorine and bromine atoms of $B(C_6F_4Br)_3$ in the solid state. For better visibility the C_6F_4Br-rings **A**, **B** and **C** are each highlighted from left to right. Ellipsoids are depicted with 50% probability level. Color code: pink – boron, grey – carbon, yellow – fluorine, red – bromine.

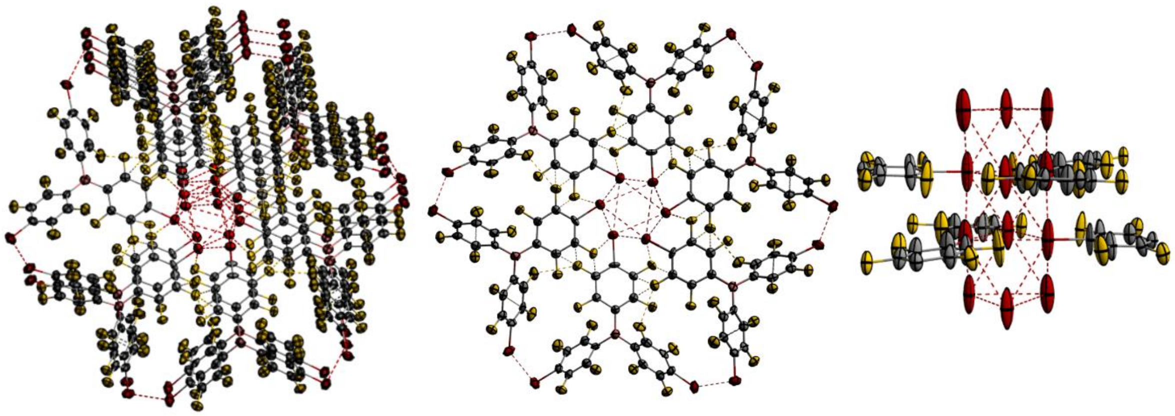

Figure S41. Intermolecular bromine-bromine contacts of $B(C_6F_4Br)_3$ in the solid state (left and middle) and intermolecular bromine-contacts of Br1A, forming a tunnel-like structure (right, remaining part of the Lewis acid is omitted). Ellipsoids are depicted with 50% probability level. Color code: pink – boron, grey – carbon, yellow – fluorine, red – bromine.

Table S34. Selected structural data of $B(C_6F_4Br)_3$ (*d* in Å, angles in °).

	$B(C_6F_4Br)_3$
d(B–C)	1.553(24) – 1.565(27)
d(C–F)	1.322(15) – 1.372(17)
d(C–Br)	1.871(18) – 1.889(18)
C–B–C	119.2(12), 119.7(12), 121.1(12)
d(F–F)	2.775(13) – 2.901(14)
d(Br–F)	3.238(20) – 3.291(8)
d(Br–Br)	3.515(6) – 3.690(3)

Table S35. Crystallographic data of $[B(C_6F_4Br)_3 \bullet (EtCN)]$.

Identification code	2469942
Empirical formula	$C_{21}H_5BBr_3F_{12}N$
Formula weight	749.80
Temperature/K	100.00
Crystal system	orthorhombic
Space group	Pbca
a/Å	14.5876(8)
b/Å	17.2488(10)
c/Å	18.4284(10)
α/°	90
β/°	90
γ/°	90
Volume/$Å^3$	4636.9(4)
Z	8
ρ_{calc} g/cm^3	2.148
μ/mm^{-1}	5.329
F(000)	2848.0
Crystal size/mm^3	0.15 × 0.07 × 0.05
Radiation	MoK_α (λ = 0.71073)
2Θ range for data collection/°	4.72 to 54.20
Index ranges	-18 ≤ h ≤ 18, -22 ≤ k ≤ 22, -23 ≤ l ≤ 23
Reflections collected	118029
Independent reflections	5124 [R_{int} = 0.0589, R_{sigma} = 0.0165]
Data/restraints/parameters	5124/0/353
Goodness-of-fit on F^2	1.089
Final R indexes [I>=2σ (I)]	R_1 = 0.0225, wR_2 = 0.0495
Final R indexes [all data]	R_1 = 0.0309, wR_2 = 0.0539
Largest diff. peak/hole / e $Å^{-3}$	0.48/-0.43

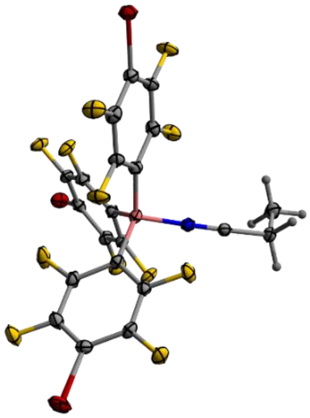

Figure S42. Molecular structure of [$B(C_6F_4Br)_3$•EtCN] in the solid state. Ellipsoids are depicted with 50% probability level. Color code: pink – boron, grey – carbon, yellow – fluorine, red –bromine, blue – nitrogen, light grey – hydrogen.

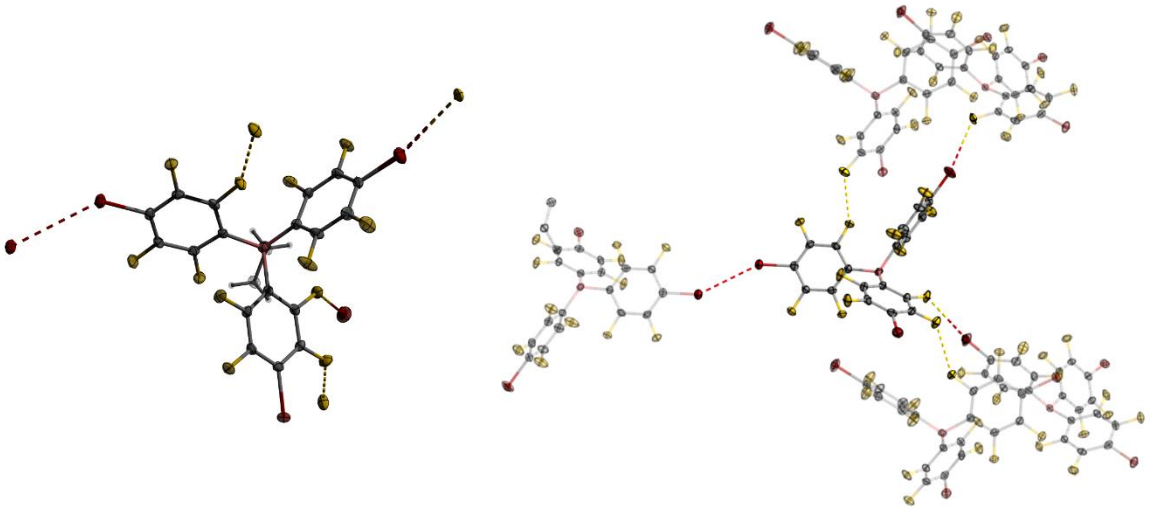

Figure S43. Intermolecular halogen contacts of fluorine and bromine atoms in the solid-state structure of [$B(C_6F_4Br)_3$•EtCN]. Ellipsoids are depicted with 50% probability level. The EtCN ligand was omitted for clarity (right). Color code: pink – boron, grey – carbon, yellow – fluorine, red –bromine, blue – nitrogen, light grey – hydrogen.

Table S36. Selected structural data of $(C_6F_4Br)_3B$•EtCN (*d* in Å, angles in °).

	$(C_6F_4Br)_3B$•EtCN
d(B–C)	1.626(3) – 1.640(3)
d(B–N)	1.596(3)
d(N–C)	1.136(3)
d(C–F)	1.342(3) – 1.359(3)
d(C–Br)	1.850(5) – 1.878(2)
C–B–C	111.6(2), 112.8(2),116.2(2)
C–B–N	103.8(2),104.1(2),107.1(2)
C-N=C/C-O-P	176.6(2)
d(F–F)	2.707(2)
d(Br–F)	3.052(4) – 3.106(5)
d(Br–Br)	3.628(1)

Table S37. Crystallographic data of $[B(C_6F_4Br)_3 \cdot (H_2O) \cdot (Et_2O)_2]$.

Identification code	2469943
Empirical formula	$C_{26}H_{22}BBr_3F_{12}O_3$
Formula weight	860.97
Temperature/K	100.00
Crystal system	monoclinic
Space group	P21/n
a/Å	10.0752(4)
b/Å	16.4661(6)
c/Å	18.3885(7)
α/°	90
β/°	91.837(2)
γ/°	90
Volume/Å^3	3049.1(2)
Z	4
ρ_{calc} g/cm^3	1.876
μ/mm^{-1}	4.071
F(000)	1680.0
Crystal size/mm^3	0.53 × 0.431 × 0.428
Radiation	MoK$_\alpha$ (λ = 0.71073)
2Θ range for data collection/°	4.54 to 52.45
Index ranges	-12 ≤ h ≤ 12, -18 ≤ k ≤ 20, -22 ≤ l ≤ 22
Reflections collected	32768
Independent reflections	6235 [R_{int} = 0.0465, R_{sigma} = 0.0341]
Data/restraints/parameters	6235/0/427
Goodness-of-fit on F^2	1.022
Final R indexes [I>=2σ (I)]	R_1 = 0.0278, wR_2 = 0.0583
Final R indexes [all data]	R_1 = 0.0404, wR_2 = 0.0623
Largest diff. peak/hole / e Å^{-3}	0.68/-0.71

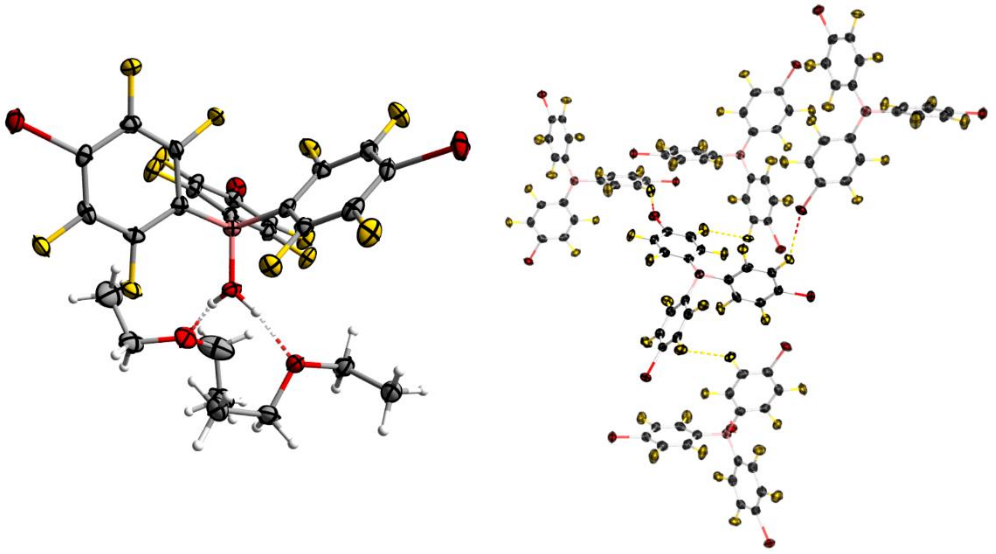

Figure S44. Molecular structure of [B(C_6F_4Br)$_3$•OH_2•2(Et_2O)] in the solid state (left) and intermolecular halogen contacts of fluorine and bromine atoms between Lewis acid units (right, Et_2O and H_2O omitted). Ellipsoids are depicted with 50% probability level and disorder of the Et_2O was omitted. Color code: pink – boron, grey – carbon, yellow – fluorine, darker red – bromine, red – oxygen, white – hydrogen.

Table S38. Selected structural data of (C_6F_4Br)$_3$B•H_2O•2Et_2O (*d* in Å, angles in °).

	(C_6F_4Br)$_3$B•H_2O•2Et_2O
d(B–C)	1.635(5) – 1.643(4)
d(B–O)	1.539(4)
d(O–H)	0.833(33) – 0.900(38)
d(C–F)	1.344(4) – 1.360(3)
d(C–Br)	1.866(3) – 1.875(3)
C–B–C	108.5(2), 113.4(2), 113.8(2)
C–B–O	104.1(2), 107.7(2), 109.1(2)
d(F–F)	2.927(2)
d(Br–F)	2.953(2)
d(Br–Br)	–

Table S39. Crystallographic data of [B(C_6F_4Br)$_3$•OPEt$_3$].

Identification code	2469944
Empirical formula	$C_{24}H_{15}BBr_3F_{12}OP$
Formula weight	828.87
Temperature/K	100.00
Crystal system	trigonal
Space group	$R\bar{3}$
a/Å	13.3560(3)
b/Å	13.3560(3)
c/Å	26.3488(9)
α/°	90
β/°	90
γ/°	120
Volume/Å^3	4070.5(2)
Z	6
ρ_{calc} g/cm^3	2.029
μ/mm^{-1}	4.621
F(000)	2400.0
Crystal size/mm^3	0.492 × 0.441 × 0.387
Radiation	MoK_α (λ = 0.71073)
2Θ range for data collection/°	4.68 to 54.76
Index ranges	-17 ≤ h ≤ 16, -17 ≤ k ≤ 16, -34 ≤ l ≤ 25
Reflections collected	10652
Independent reflections	2069 [R_{int} = 0.0301, R_{sigma} = 0.0247]
Data/restraints/parameters	2069/0/146
Goodness-of-fit on F^2	1.075
Final R indexes [I>=2σ (I)]	R_1 = 0.0279, wR_2 = 0.0594
Final R indexes [all data]	R_1 = 0.0336, wR_2 = 0.0612
Largest diff. peak/hole / e Å^{-3}	0.36/-0.48

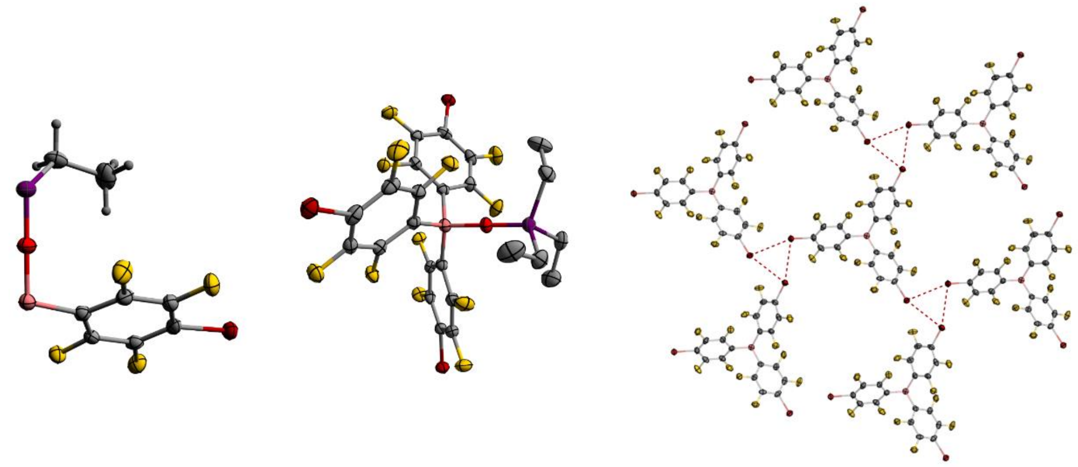

Figure S45. Asymmetric unit (left), the whole molecular structure (middle) of [B($C_6F_4Br)_3$•$OPEt_3$] and intermolecular bromine-bromine contacts (right, $POEt_3$ ligand is omitted) in the solid-state. Ellipsoids are depicted with 50% probability level. Disorder of the $POEt_3$ and hydrogen atoms were omitted. Color code: pink – boron, grey – carbon, yellow – fluorine, darker red – bromine, red – oxygen, violet – phosphorous.

Table S40. Selected structural data of $(C_6F_4Br)_3B$•$POEt_3$ (*d* in Å, angles in °).

	$(C_6F_4Br)_3B$•$OPEt_3$
d(B–C)	1.637(3)
d(B–P)	1.529(5)
d(O–P)	1.502(3)
d(C–F)	1.345(3) – 1.354(3)
d(C–Br)	113.0(2)
C–B–C	105.6(2)
C–B–P	180.0
d(F–F)	–
d(Br–F)	–
d(Br–Br)	3.637(6)

References

[28] V. V. Bardin, *Russ. Chem. Bull.* **1997***, 46,* 780.

[29] V. V. Bardin, L. S. Pressman, L. N. Rogoza, G. G. Furin, *J. Fluor. Chem.* **1991**, *53*, 213..

[38] P. Erdmann, J. Leitner, J. Schwarz, L. Greb, *ChemPhysChem* **2020**, *21*, 987.

[40] M. A. Beckett, G. Strickland, J. R. Holland, K. S. Varma, *Polymer* **1996**, *37*, 4629.

[45] G. R. Fulmer, A. J. M. Miller, N. H. Sherden, H. E. Gottlieb, B. M. Stoltz, J. E. Bercaw, K. I. Goldberg, *Organometallics* **2010**, *29*, 2176.

[46] R. K. Harris, E. D. Becker, S. M. Cabral de Menezes, P. Granger, R. E. Hoffman, K. W. Zilm, *Pure Appl. Chem.* **2008**, *80*, 59.

[47] MestReNova 14.3.0-30573, Mestrelab Reasearch S.L. **2022**.

[48] OriginPro, Version 2024. OriginLab Corporation, Northhampton, MA, USA.

[49] O. V. Dolomanov, L. J. Bourhis, R. J. Gildea, J. A. K. Howard, H. Puschmann, *J. Appl. Cryst.* **2009**, *42*, 339.

[50] G. M. Sheldrick, *Acta Cryst.* **2015**, *A71*, 3.

[51] G. M. Sheldrick, SHELXL Version 2014/7, Program for Crystal Structure Solution and Refinement; Göttingen, Germany, 2014.

[52] G. M. Sheldrick, *Acta Cryst.* **2008**, *A64*, 112.

[53] K. Brandenburg, Diamond: Crystal and Molecular Structure Visualization http://www.crystalimpact.com/diamond.

[54] Persistence of Vision Pty. Ltd. Persistence of Vision Raytracer. Ltd., Persistence of Vision Pty. 2004.

[55] Gaussian 16, Revision C.01, M. J. Frisch, G. W. Trucks, H. B. Schlegel, G. E. Scuseria, M. A. Robb, J. R. Cheeseman, G. Scalmani, V. Barone, G. A. Petersson, H. Nakatsuji, X. Li, M. Caricato, A. V. Marenich, J. Bloino, B. G. Janesko, R. Gomperts, B. Mennucci, H. P. Hratchian, J. V. Ortiz, A. F. Izmaylov, J. L. Sonnenberg, D. Williams-Young, F. Ding, F. Lipparini, F. Egidi, J. Goings, B. Peng, A. Petrone, T. Henderson, D. Ranasinghe, V. G. Zakrzewski, J. Gao, N. Rega, G. Zheng, W. Liang, M. Hada, M. Ehara, K. Toyota, R. Fukuda, J. Hasegawa, M. Ishida, T. Nakajima, Y. Honda, O. Kitao, H. Nakai, T. Vreven, K. Throssell, J. A. Montgomery, Jr., J. E. Peralta, F. Ogliaro, M. J. Bearpark, J. J. Heyd, E. N. Brothers, K. N. Kudin, V. N. Staroverov, T. A. Keith, R. Kobayashi, J. Normand, K. Raghavachari, A. P. Rendell, J. C. Burant, S. S. Iyengar, J. Tomasi, M. Cossi, J. M. Millam, M. Klene, C. Adamo, R. Cammi, J. W. Ochterski, R. L. Martin, K. Morokuma, O. Farkas, J. B. Foresman, and D. J. Fox, Gaussian, Inc., Wallingford CT, 2016.

[56] M. D. Hanwell, D. E. Curtis, D. C. Lonie, T. Vandermeersch, E. Zurek, G. R. Hutchison, *J. Cheminformatics* **2012**, *4*, 17.

[57] G. A. Zhurko, in ChemCraft, http://www.chemcraftprog.com.

[58] M. K. Manoj, B. Brauer, J. M. L. Martin, *J. Phys. Chem. A* **2015**, *119*, 1701.
